# Hepatoma derived growth factor binds DNA through the N-terminal PWWP domain

**DOI:** 10.1186/1471-2199-8-101

**Published:** 2007-10-31

**Authors:** Jun Yang, Allen D Everett

**Affiliations:** 1 Department of Pediatrics, Division of Pediatric Cardiology, Johns Hopkins University, Baltimore, MD 21205, USA

## Abstract

**Background:**

Hepatoma Derived Growth Factor (HDGF) is a nuclear protein with nuclear targeting required for mitogenic activity. Recently we demonstrated that HDGF is a transcriptional repressor, but whether HDGF binds DNA, the specificity of DNA binding and what protein domain is required are still unknown. In this study, we aimed to identify if HDGF is a DNA binding protein, map the functional DNA binding domain and DNA binding element for HDGF.

**Results:**

Using chromatin immunoprecipitation (ChIP) of human DNA, we isolated 10 DNA sequences sharing a conserved ~200 bp element. Homology analysis identified the binding sequences as a motif within the promoter of the SMYD1 gene, a HDGF target gene. Electrophoretic Mobility Shift Assays (EMSA) confirmed the binding of HDGF to this conserved sequence. As a result, an 80 bp conserved sequence located in the SMYD1 promoter bound GST-HDGF tightly. The binding core sequence for HDGF was narrowed down to 37 bp using a deletion mapping strategy from both the 5' and 3' ends. Moreover, ChIP and DNase I footprinting analysis revealed that HDGF binds this 80 bp DNA fragment specifically. Functionally overexpression of HDGF represses a reporter gene which is controlled by an SV-40 promoter containing the 80 bp DNA element. Using serial truncations of GST-HDGF, we mapped the DNA binding domain of HDGF to the N-terminal PWWP domain.

**Conclusion:**

HDGF is a DNA binding protein, binds DNA specifically, and prefers a minimum of 37 bp long DNA fragment. The N-terminal PWWP domain of HDGF is required for DNA binding. HDGF exerts its transcription repressive effect through binding to a conserved DNA element in the promoter of target genes.

## Background

Hepatoma derived growth factor (HDGF) is a nuclear protein with mitogenic activity [1,2]. It is highly expressed in developing heart and fetal gut [3]; and re-expressed in vascular smooth muscle cells in vivo after vascular injury [4,5], suggesting that it plays an important role in cardiovascular growth and differentiation. Recently by a number of investigators, HDGF was found to be tumorigenic and a prognostic factor for a number of cancers [6-11]. We have discovered that HDGF functions as a transcriptional repressor, suggesting that HDGF may physiologically regulate cellular proliferation and differentiation by repressing the genes governing terminal differentiation (submitted). However using NMR structural analyses, it is unclear if HDGF is a direct DNA binding protein [12] and controversial if the conserved N-terminal PWWP domain in HDGF is involved in DNA binding [13].

In the present study, using a modified chromatin immunoprecipitation (ChIP) assay, we found that HDGF bound a conserved ~200 base pair sequence common to an HDGF target gene promoter. EMSA and DNase I footprinting analysis revealed that HDGF-DNA interaction is specific. In EMSA mapping studies, we found HDGF bound a minimal 37 bp long oligonucleotide and DNA binding required the HDGF N-terminal PWWP domain. Furthermore, this DNA sequence is functionally significant as HDGF represses a reporter gene which was controlled by the identified DNA element.

## Results

### HDGF binds to DNA

**Figure 1:**
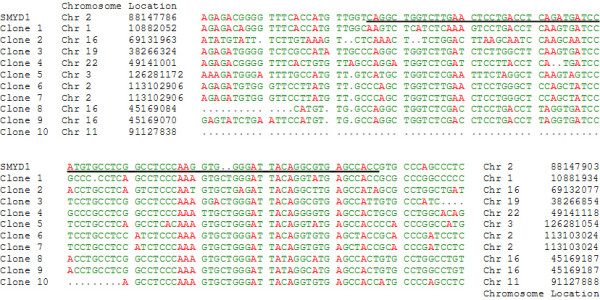
**Alignment of ChIP clones identify a HDGF binding motif consensus with the SMYD1 gene promoter**. ChIP was carried out as described in method. Totally 100 clones were sequenced, and 10 of them share a conserved region. The figure shows that these 10 clones were aligned with human SMYD1 gene promoter homologue region using ClustalW program. The genomic and chromosome location were indicated at the beginning and ending of each sequences. The solid line indicates the sequence of the 80 bp probe for EMSA.

**Figure 2:**
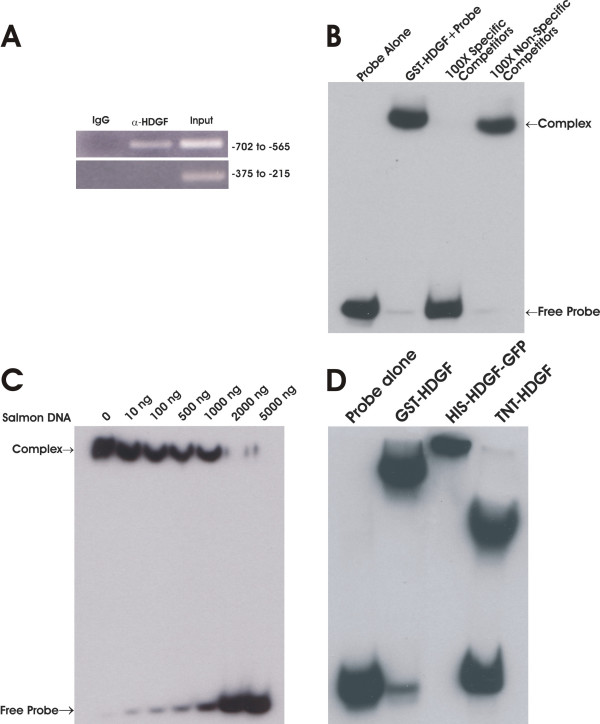
**HDGF binds DNA**. (A) HDGF binds DNA in vivo. ChIP was carried out as described in method. A specific HDGF polyclonal antibody was used to precipitate Hela chromatin, GFP antibody was used as negative control. Recovered DNA was amplified by PCR using specific primers. The PCR primers were derived from the human SMYD1 promoter as described in methods. The PCR products were analyzed by ethidium bromide staining of a 2% agarose gel. A primer set targeting a proximal site (-375 to -215) of the SMYD1 promoter was used as a negative control. The numbers on the right indict the location of amplicons in SMYD1 promoter. (B) HDGF binds DNA in vitro. 0.25 pmol 5' end biotin labeled probes for SMYD1 promoter (nucleotides -688 to -609) were incubated with 1 ug GST-HDGF fusion protein in the reaction buffer in the presence of either 100 × specific or non specific competitors (B) or various amount of sheared salmon sperm DNA (C) on ice for 30 min. The complex were resolved in 5% PAGE and transferred onto a nylon membrane. The biotin end-labeled DNA probe is detected using a streptavidin-horseradish peroxidase conjugate and chemiluminescent substrate. (D) Different sources of HDGF bind to DNA. 1 ug of bacterial expressed GST-HDGF, His-HDGF-GFP and in vitro translated HDGF (without tag) were used for EMSA as described above.

HDGF is a nuclear protein [1,2] and we recently have shown that it functions as a transcriptional repressor (submitted). However, whether HDGF binds DNA directly and if so, it's DNA binding specificity are both unknown. To functionally identify the HDGF DNA binding sequence we used a modified ChIP assay to identify homologous sequences, if any, as potential DNA binding motifs. After 100 clones were sequenced, sequence alignment analysis found that there was a group of 10 sequences that shared a ~200 bp highly conserved sequence (Fig. 1, Additional File 1 and 3). No homologous sequences were identified in 50 clones which were immunoprecipitated by a GFP control antibody. A highly conserved copy was found within the promoter of SMYD1, a HDGF target gene through database searching of the human genome (NCBI BLAST). We found this conserved element located at the promoter -600 to -880 bp proximal to the start codon. Alignments of the ChIP sequences with the SMYD1 promoter identified a highly conserved 80 bp motif (Fig. 1). By ChIP-PCR, we successfully amplified this specific region (-702 to -565) from immunoprecipitated chromatin DNA using a HDGF specific antibody (Fig. 2A). As a control, no amplification was observed for a proximal site (-375 to -215). We then verified HDGF DNA binding using an in vitro competition EMSA assay. As shown in Figure 2B, the labeled 80 bp probe bound tightly to GST-HDGF. The binding was specific as 100 fold of the non-labeled 80 bp probe totally abolished the binding; however 100 fold of a nonspecific oligonucleotide competitor had no effect on the binding. Salmon sperm DNA is known as a strong competitor for DNA binding. However, as shown in Figure 2C, the binding of the 80 bp probe to HDGF could be blocked only when the concentration of salmon sperm DNA reached 2 ug, which is approximately a 30,000 molar fold excess to the input labeled oligo (0.25 pmol). In addition, HDGF binding was not an artifact of using GST tagged HDGF as an EMSA using another tagged protein, His-HDGF, or non-tagged in vitro translated HDGF, all demonstrated equivalent tight binding to the EMSA probe (Fig. 2D). From these results, we conclude that HDGF binds an 80 bp DNA sequence conserved within the promoter of the HDGF target gene SMYD1.

### HDGF binds a 37 bp long DNA motif specifically

**Figure 3:**
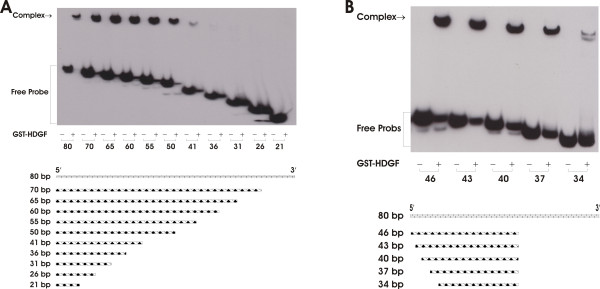
**HDGF requires a minimal 37 bp DNA sequence for binding**. Sequential 3'(A) or 5' (B) end serial deleted (3–5 bp) probes were synthesized and used in an EMSA to identify a minimum DNA binding sequence. The bottom schematic diagram elucidates the locations of deleted probes relative to the 80 bp long probe. A 37 bp probe was identified as the minimal DNA binding sequence.

To determine the HDGF core binding sequence, we made serial deletion oligonucleotides from the 3' end of the 80 bp probe, which we tested by EMSA for HDGF binding. As shown in Figure 3A, the binding was completely lost with a probe shorter than 50 bp. To further narrow down the DNA recognition motif, we first took out another 4 bp at the 3' end of this 50 bp probe in Figure 3A, then made another serial deletion set from the 5' end based on the 46 bp probe. As shown in Figure 3B, incremental deletion of the 5' end of the 46 bp probe, identified a 37 bp motif that is required for HDGF binding. A 37 bp DNA motif required for DNA binding is relatively long. In fact we could not detect any HDGF DNA binding when we used 5 short DNA probes which are all 15 bp long with a 5 bp overlap, covering the central part of the 80 bp oligo (Data not shown).

**Figure 4:**
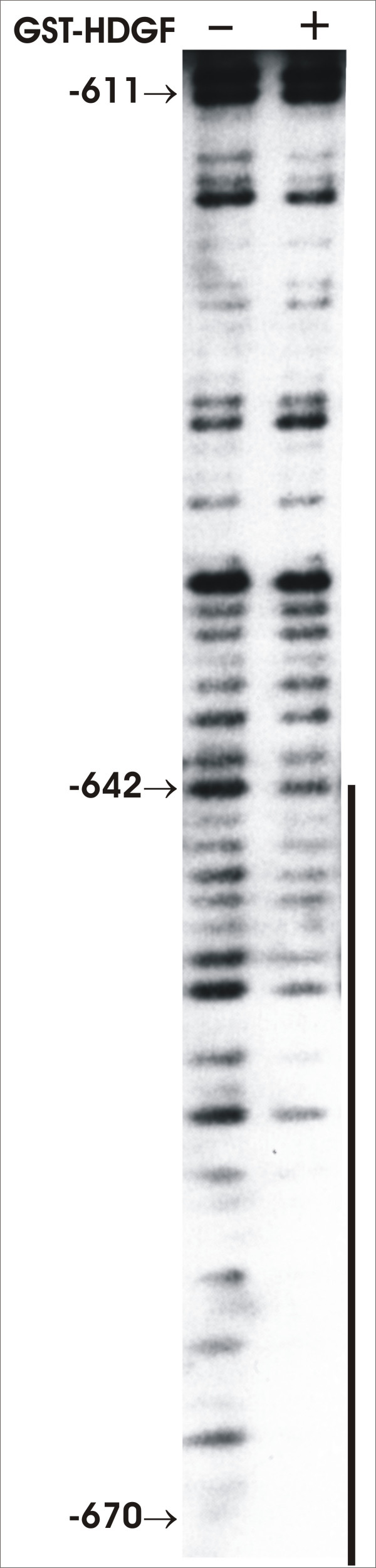
**HDGF protect DNA from DNase I digestion**. DNase I footprinting analysis of the 80 bp DNA fragment using GST-HDGF recombinant protein. The DNA fragment is the same as the probe used in EMSA corresponding to nucleotides -688 to -609 of the SMYD1 gene promoter. Numbers on the left side indicate the location in the SMYD1 promoter. Protected sequence is indicated with single line.

We also performed DNase I footprinting assay to determine the specificity of HDGF-DNA interaction. As shown in Figure 4, GST-HDGF can protect part of the 80 bp DNA fragment from DNase I digestion. The DNA fragment used in footprinting analysis is the same as the probe used in EMSA. The protected region starts from 34 bp from the 3' end (which correlates to the -642 bp in SMYD1 gene promoter) to 5' end. We could not get a clear footprint of 5' end because it is too close to the 5' of the DNA fragment we used. However, the result is consistent with the data of EMSA, that the 5' of the 80 bp DNA fragment is critical for HDGF-DNA interaction. These data indicated that HDGF binds DNA specifically, but the binding requires a large DNA molecule.

### Overexpression of HDGF represses a reporter gene when controlled by the HDGF binding element

**Figure 5:**
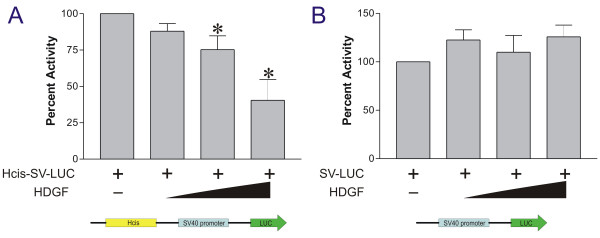
**HDGF represses the transcription of a reporter which is controlled by the HDGF DNA binding element**. The 80 bp oligo (Hcis) was cloned upstream of the SV40 promoter which controls a downstream luciferase reporter gene. 0.5 ug Hcis-SV40-LUC reporter construct (A) or SV40-LUC control reporter (B) were cotransfected with various amount (0.1, 0.3, 0.5 ug) of GFP-HDGF. 0.1 ug pRL-CMV were included as transfection control. The dual-luciferase reporter assay system was used to measure the luciferase activities 24 hours after transfection. Percentage of firefly luciferase activity was calculated when luciferase activity from the reporter gene alone was set at 100%. Firefly luciferase activity was normalized with constitutive renilla luciferase activity to correct for transfection efficiency. *Bars *indicate mean ± SEM from 3 separate transfection assays with duplicate plates, *P < 0.05 by one-way ANOVA control versus GFP-HDGF.

In order to study whether the HDGF binding element in SMYD1 gene promoter is functional, we cloned the 80 bp oligo described above upstream of the SV40 promoter, so that the HDGF binding element (Hcis)-SV40 controls the downstream luciferase gene. Co-transfection of HDGF with this reporter (Hcis-SV40-LUC) significantly repressed the reporter gene activity in a dose dependent manner (Fig 5A). As a control, overexpression of HDGF had no effect on the reporter without the HDGF binding element (Fig 5B). These experiments indicate that HDGF exerts its repressive effect through binding with the HDGF binding element directly.

### PWWP domain is required for DNA binding

**Figure 6:**
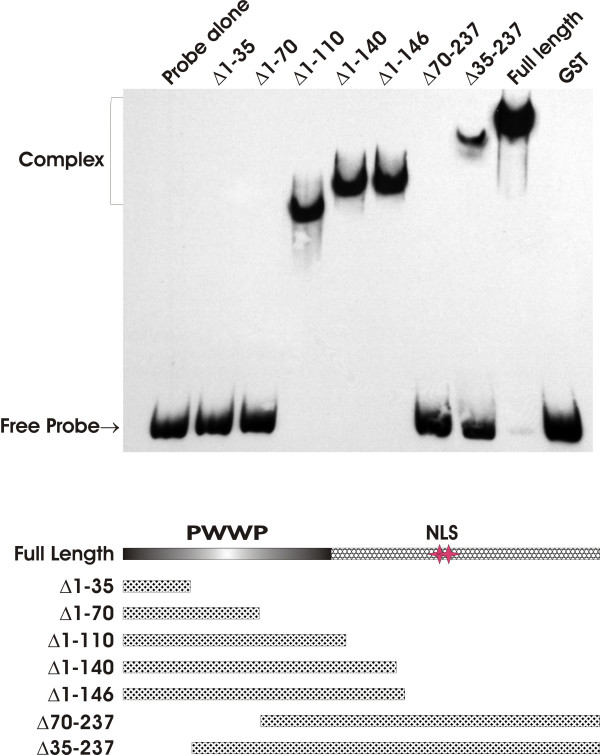
**PWWP domain is required for HDGF DNA binding**. EMSA was carried out as described above using truncated recombinant HDGF protein. The bottom panel is a schematic illustration of the GST-HDGF truncated polypeptides generated to determine the region(s) of HDGF that allow for binding with the 80 bp DNA probe. The PWWP domain is located at amino acids 1–90. NLS represents the location of the HDGF nuclear localization sequence.

A PWWP domain spanning approximately 90 AA is present in the N-terminal of HDGF [14]. Although controversial, the PWWP domain is reported to have the capability of binding DNA as has been shown for DNA methyltransferase 3B (DNMT3b) [13,15-17]. In order to study whether the HDGF PWWP domain is responsible for DNA binding, we made several truncated HDGF fusion proteins to test for DNA binding in EMSA experiments. As shown in Figure 6, only the truncated proteins which contain the whole PWWP domain can bind to DNA (Δ1–110, Δ1–140, Δ1–146), whereas proteins lacking the complete PWWP domain can not bind to DNA. Importantly the entire PWWP domain is required as fusions containing a portion of the PWWP domain could not bind (Δ70–237) or bound weakly (Δ35–237).

## Discussion

HDGF is nuclear target protein with nuclear localization necessary for its mitogenic function [1,2]. Because of these attributes, it has been hypothesized that HDGF, especially considering it contains a PWWP domain, is a DNA regulatory protein. In this study, we found that HDGF binds directly to DNA via a specific element contained in the promoter of a HDGF target gene, SMYD1. Functionally this DNA binding element also confers transcriptional repression activity. SMYD1 gene is essential for cardiomyocyte differentiation and cardiac morphogenesis [18], the regulation of SMYD1 by HDGF is in accord with the functional importance of HDGF in the development of cardiovascular system [3,4], likely playing a role in preventing terminal differentiation.

We found that HDGF prefers to bind a large DNA molecule, requiring a minimum 37 bp DNA fragment. It is surprising that HDGF binds such a long DNA fragment. In a recent structural study on the interaction of the HDGF PWWP domain with DNA, we identified unique alterations in the structure of the HDGF PWWP domain with the addition of DNA. However we could not confirm whether these interactions with DNA were specific [12]. This was due to the technical requirements of both SAAB and NMR for oligonucleotides shorter than 30 bp. In the present study, with EMSA and longer oligonucleotides we found that the length of DNA fragment is critical for HDGF binding. HDGF tightly binds DNA only when the DNA is ≥37 bp, suggesting that this HDGF/DNA interaction may require a unique DNA structure.

Transcription factor binding sites are usually short, highly degenerate, and often require specific tertiary structures [19]. For example estrogen receptor response element (ERE) is a palindromic motif. However when we analyzed the 37 bp core sequence for HDGF binding using EXTRACTFEAT program [20], we could not identify any special structure such as palindromes or repeated structures within this 37 bp core HDGF binding sequence. It is of interest that the 37 bp HDGF DNA binding element is a component of an Alu DNA repeat. The Alu DNA family is the most abundant members of the short interspersed repeated DNA elements (SINEs) which are found in the genomes of primates exclusively [21,22]. Alu sequences are approximately 300 bp in length, have a bipartite structure with left and right arm monomers which are derived from the 7SL RNA gene. The overall function of these repeats is unclear, but of interest to the present study, these repeats containing the HDGF binding sequence are found within the promoters of all HDGF target genes identified so far [see Additional File 2].

HDGF is evolutionary relative new. Besides mammals, HDGF homologs also exist in other organisms, such as xenopus and rodents. Although Alu DNA is found exclusively in primates, there is homologous SINE DNA in rodents such as the B1 and B2 DNA elements of the mouse. More interestingly, the mouse genome is estimated to contain 10,000 copies of a retroposon family that is closely related to human Alu repeats [23]. Comparative study of the DNA binding in both human and mouse will give us more information on the conservation of this binding element.

The N-terminus of HDGF contains a PWWP domain, a weakly conserved 90 amino acid motif, originally identified in the WHSC1 gene [24] and as the HATH (homologous to the amino terminus of HDGF) region in the HDGF family of proteins [14], it has been found in more than 60 eukaryotic proteins [24]. Functionally, most of the PWWP family proteins are involved in chromatin remodeling [25]. However the role of the PWWP domain in this function is unknown. We demonstrate the first functional significance of the PWWP domain as it is necessary and sufficient for binding to the HDGF DNA binding element. The PWWP domain was hypothesized to be a site for protein-protein interactions [24]. However, the PWWP domain of DNMT3b was shown to interact with DNA [15-17]. In a more detailed investigation, the PWWP domain of DNMT3b was found to bind major satellite DNA nonspecifically [17]. However, the PWWP in its closest homologue gene, DNMT3a (60.3% identity) had no DNA binding activity [17]. This suggests that small changes in the PWWP domain may have a significant impact on its DNA binding ability. As the PWWP domain in HDGF is only 25.9% homologous with DNMT3a, it is not surprising that HDGF could have a different DNA binding behavior.

A recent structural study found that under physiological conditions, both PWWP modules and full length human HDGF could form dimers [26]. Using the SPR method, the authors found that PWWP dimer binds to heparin with higher affinity than that of a monomeric PWWP module. Several sequence specific DNA binding protein such as C/EBP, c-Myc, c-Jun and fos form hetero/homo dimers. Their leucine-zipper motifs do not face with the DNA. Rather, they form three-dimensional "scaf-carboxylfolds" that match the contour of DNA [27]. Whether HDGF dimerizes on DNA and utilizes the similar mechanism of DNA recognition remains to be determined.

## Conclusion

We have shown that HDGF is a DNA binding protein; specifically binds a DNA element in the promoter of SMYD1 gene. This DNA/HDGF interaction is unique in that the minimum required binding element was 37 bp in length. Importantly the poorly understood N-terminal PWWP domain of HDGF was responsible for DNA binding. Taken together HDGF functions in the nucleus as a direct DNA binding protein to repress the expression of specific target genes to likely regulate cell proliferation and differentiation.

## Methods

### Plasmids Construction

HDGF expression construct (pK7-GFP-HDGF) was described before [1]. Human HDGF was PCR amplified from IMAGE clone 5587366 (ATCC) and subcloned in frame with GST in pGEX-4T2 vector (Amersham Pharmacia Biotech) by introducing appropriate restriction sites. Truncated GST-HDGF constructs were generated by PCR cloning method. Hcis-SV40-LUC reporter was generated by inserting the 80 bp oligo which was used in EMSA into the upstream of SV40 promoter in pGL3 control plasmid (Promega). All new constructs were confirmed by sequencing.

### ChIP Assay

The manufacturer's (Upstate) protocol was used for formaldehyde cross-linking and chromatin immunoprecipitation. A rabbit anti-HDGF polyclonal antibody [1] or a rabbit anti-GFP antibody (Santa Cruz) as a control, were used to precipitate chromatin from 2 × 10^7 ^Hela cells. After reverse crosslinking and purification, DNA was recovered and divided into two fractions. For the cloning experiment, recovered DNA was ligated with a PCR linker [28], the linker sequence is 5'-GCGGTGACCCGGGAGATCTGAATTC-3'. The ligation product was subjected to PCR using linker specific primers (the same oligo used as linker above). PCR products were cloned into pCRScript vector according to manufacturer's instruction (Stratagene). 100 clones were picked up and sequenced. For ChIP-PCR experiment, recovered DNA from each sample was amplified by PCR using specific primers. PCR was carried out as follows: 1 ul of DNA sample, 0.5 uM each primer, 1.5 mM MgCl_2_, 0.2 mM each dNTPs, 1 × *Taq *buffer (Bio-Rad), 1.25 units of *Taq *DNA polymerase (Bio-Rad) in a total volume of 25 ul. After 40 cycles of amplification, the PCR products were analyzed by ethidium bromide staining of a 2% agarose gel. Primers used for PCR of ChIP samples were designed directly from human SMYD1 promoter sequence obtained from the public data base (The UCSC Genome Browser). Primer sequences are: Forward 5'-TCACCATGTTGGTCAGGCTGGTCT-3' (-702 to -679 of start codon), Reverse 5'-AGGGTGGACTGTTTAGCAGC-3' (-584 to -565 of start codon). The second primer set which located at -375 to -215 was used as control. The sequences are: 5'-AGTGCAAGCCTGACAGCTGAAGG-3' and 5'-GGAAGAGTTTCATTCATCACCCAGC-3'.

### Electrophoretic Mobility Shift (EMSA) Assay

GST-HDGF and truncated protein were expressed in the BL21 (DE3) strain of *Escherichia coli *using the pGEX-4T2 vector (Amersham Pharmacia Biotech), His-HDGF-GFP was expressed in pG7-HDGF [1] was also expressed in BL21 strain, the purification of GST and His recombinant proteins were followed the manufacture's instructions. TNT-HDGF protein was translated in vitro using the TNT kit (Promega) and a pcDNA3.1 based expression plasmid encoding full-length HDGF without tag. EMSA was performed according to manufacture's instruction (Pierce, LightShift Chemiluminescent EMSA Kit). Briefly, 1 ug purified recombinant HDGF or truncated HDGF protein was incubated with 0.25 pmol 5' end biotin labeled double-stranded oligonucleotide in the reaction buffer (10 mM Tris pH 7.5, 50 mM KCl, 1 mM DTT, 5% glycerol, 0.05% NP-40, 0.1 ug sheared salmon sperm DNA) for 30 min on ice. The reaction mixture was loaded onto a 5% non-denaturing polyacrylamide gel. The gel was run in 0.1 × TBE buffer at 200 V for 2 hours. DNA-protein complex were transferred to a nylon membrane in 0.5 × TBE buffer and UV crosslinked. The biotin end-labeled DNA probe was detected using a streptavidin-horseradish peroxidase conjugate and the chemiluminescent substrate. The 80 bp double stranded oligo probe is located at -688 to -609 of SMYD1 promoter (+1 is the start codon), the sequence of biotin labeled sense strand is 5'-Bio-CAGGCTGGTCTTGAACTCCTGACCTCAGATGATCCATGTGCCTCGGCCTCCCAAGGTGGGGATTACAGGCGTGAGCCACC-3'. The sequence of sense strand of non-specific competitor is 5'-TGCTGTTGACAGTGAGCGCGGCCAGCTTATAGTCATATATTAGTGAAGCCACAGATGTAATATATGACTATAAGCTGGCCTTGCCTACTGCCTCGGA. All of the DNA probes were synthesized by IDT DNA Inc.

### DNase I footprinting analysis

We used a non-radioactive detection method for DNase I footprinting analysis. DNA fragment covers -688 to -609 of SMYD1 promoter (+1 is the start codon) was used in this experiment, the sequence is the same as the 80 bp oligo probe used in EMSA. Binding reactions (100 μL) comprised 1 ug purified recombinant HDGF protein, 0.25 pmol double-stranded oligonucleotide (5' end of sense strand was labeled by biotin), 10 mM Tris pH 7.5, 50 mM KCl, 1 mM DTT, 5% glycerol, 0.05% NP-40, 0.1 ug sheared salmon sperm DNA, the reaction mixture was incubated on ice for 30 min. One volume of 10 mM MgCl_2 _5 mM CaCl_2 _was added. Each reaction mixture was then treated with increasing amounts of DNase I, from 0.01 to 0.1 Kunitz units (Worthington) for 120 seconds and stopped by adding 200 μL stop solution (1% sodium dodecyl sulphate, 200 mM NaCl, 20 mM EDTA pH 8.0, 40 mg/mL yeast tRNA). Reactions were extracted once with one volume of phenol-chloroform, and precipitated with two volumes of ethanol at -80°C for 60 minutes. Reactions were centrifuged for 15 minutes at 13,200 g. DNA pellets were washed with 80% ethanol and air dried. Pellets were resuspended in 5 μL of loading buffer (95% formamide, 20 mM EDTA, 0.05% Bromophenol Blue, 0.05% Xylene Cyanol FF). Reactions were loaded on a 6% (19:1 acrylamide:bis-acrylamide) polyacrylamide sequencing gel containing 7 M urea and 1 × TBE (89 mM Tris base, 89 mM boric acid, 2.5 mM EDTA). The gel was run at 50 watts in 1 × TBE buffer. The DNA fragments were transferred to a charged Nylon membrane and detected using Chemiluminescent Nucleic Acid Detection Module (Pierce, 89880).

### Cell Culture and Transient Transfection

G7 myoblast cells were purchased from ATCC and maintained in Dulbecco's Modified Eagle's medium (DMEM) with 10% fetal calf serum and 10% horse serum (ATCC). Transient transfection assays were performed using FuGENE6 (Roche) according to the manufacturer's instruction. G7 cells in 6 well plates were transfected with 0.5 ug of Hcis-SV40-LUC reporter construct and 0.1–0.5 ug of pK7-GFP-HDGF or pK7-GFP vector as control, 0.1 ug of pRL-CMV was cotransfected as internal control, the total DNA concentration was held constant by adding empty vector. 24 hours after transfection, Luciferase assays were performed using a Dual-Luciferase Reporter Assay Systems (Promega). 20 ul of cell lysate was measured in a Monolight 2010 luminometer (Analytical Luminescence Laboratories) as described in the manufacturer's manual. Firefly luciferase activity was normalized for Renilla luciferase activity. All transfection data are represented as the mean ± SEM of at least 3 independent experiments performed in duplicate.

## Authors' contributions

AE and JY participated in the design of the study, drafting and revising the manuscript. AE carried out the ChIP assay and cloning of DNA fragments from ChIP. JY carried out sequences alignment, EMSA, ChIP-PCR, DNase I Footprinting and reporter gene assay. All authors read and approved the final manuscript.

## Supplementary Material

Additional file 1 Location of ChIP sequences within human genome. The data shows the chromosome location and alignment of ChIP clones 1–10 to human genome. Click here for file

Additional file 3 Genomic location of ChIP clones. The data shows the chromosome location and alignment of non-specific ChIP clones 11–100 to human genome. Click here for file

Additional file 2 The alignment of binding core in 5 candidate gene promoters. The data shows the location of binding core in the promoters of 5 candidate genes, and their alignment. Click here for file
