# Supplementary material for: Hepatoma derived growth factor binds DNA through the N-terminal PWWP domain
Source: BMC Mol Biol. 2007 Oct 31;8:101. doi: 10.1186/1471-2199-8-101 (PMC2176068; doi:10.1186/1471-2199-8-101)
Supplement: Additional file 1 — Location of ChIP sequences within human genome. The data shows the chromosome location and alignment of ChIP clones 1–10 to human genome. [file 1471-2199-8-101-S1.pdf]

Additional Data 1

Location of ChIP sequences within human genome:

Blat Program UCSC Genome Browser (http://genome.ucsc.edu/) was used to search for the homology region in human genome. The alignments of each ChIP sequences and human genome show the exact location of the sequences in human genome.

Alignment of Clone 1 and chr1:10881867-10882187

Click on links in the frame to the left to navigate through the alignment. Matching bases in cDNA and genomic sequences are colored blue and capitalized. Light blue bases mark the boundaries of gaps in either sequence (often splice sites).

Clone 1

```
aggctGGAGT GCAGTGgcat GATCTtgGcT CACTGCAACC TCTGCCTCCt  50
GGgTTCAaGc GATTCTCCTg CCTCAGCCTC CTGAGTAGCT GgGAcTACAG  100
GtGcccGCCA cCaCaCcTGG CTAATTTTTG TATTTTTAGT AGAGACAGGG  150
TTTCACCATG TTGGCAAGTC TCATCTCAAA GTCCTGACCT CAAGTGATCC  200
GCCCCCTCAG CCTCCCAAAG TGCTGGGATT ACAGGTATGA GCCACCGCGC  250
CCGGCCCCCT ACAGGGTATT TTCTATGTCC TAAAGCCTTC TTTGAGTGTG  300
AGGCTTCAAT TATTGTTTTT ACGAAG
```

Genomic chr1 (reverse strand):

```
caggaccctt ccttccttcc ttcctttctt ccttcctccc tcccttcctc  10882238
cctttctctc tctctctctt tgagacagtc ttgctcttgt cacccagacc  10882188
GGAGTGCAGT GatgcGATCT caGtTCACTG CAACCTCTGC CTCCcGGaTT  10882138
CAgGgGATTC TCCTaCCTCA GCCTCCTGAG TAGCTGtGAt TACAGGcGtg  10882088
tGCCAtCgCg CaTGGCTAAT TTTTGTATTT TTAGTAGAGA CAGGGTTTCA  10882038
CCATGTTGGC AAGTCTCATC TCAAAGTCCT GACCTCAAGT GATCCGCCCC  10881988
CTCAGCCTCC CAAAGTGCTG GGATTACAGG TATGAGCCAC CGCGCCCGGC  10881938
CCCCTACAGG GTATTTTCTA TGTCCTAAAG CCTTCTTTGA GTGTGAGGCT  10881888
TCAATTATTG TTTTTACGAA Gagtcatggt ggcagcagag ggtaccagag  10881838
acctggcagc tcctggtctg ttccccagcc atggggcatg ttcatgtgga  10881788
cagaaggaga gcgggagggc c
```

Side by Side Alignment

```
00000006 ggagtgcagtggcatgatcttggctcactgcaacctctgcctcctgggtt 00000055
<<<<<<<< |||||||||||    |||||  | |||||||||||||||||||| || || <<<<<<<<
10882187 ggagtgcagtgatgcgatctcagttcactgcaacctctgcctcccggatt 10882138

00000056 caagcgattctcctgcctcagcctcctgagtagctgggactacaggtgcc 00000105
<<<<<<<< || | ||||||||| ||||||||||||||||||||| || |||||| |   <<<<<<<<
10882137 caggggattctcctacctcagcctcctgagtagctgtgattacaggcgtg 10882088

00000106 cgccaccacacctggctaatttttgtatttttagtagagacagggtttca 00000155
<<<<<<<<  |||| | | | |||||||||||||||||||||||||||||||||||||| <<<<<<<<
10882087 tgccatcgcgcatggctaatttttgtatttttagtagagacagggtttca 10882038

00000156 ccatgttggcaagtctcatctcaaagtcctgacctcaagtgatccgcccc 00000205
<<<<<<<< |||||||||||||||||||||||||||||||||||||||||||||||||| <<<<<<<<
10882037 ccatgttggcaagtctcatctcaaagtcctgacctcaagtgatccgcccc 10881988

00000206 ctcagcctcccaaagtgctgggattacaggtatgagccaccgcgcccggc 00000255
```

```
<<<<<<<< |||||||||||||||||||||||||||||||||||||||||||||||||| <<<<<<<<
10881987 ctcagcctcccaaagtgctgggattacaggtatgagccaccgcgcccggc 10881938

00000256 cccctacagggtattttctatgtcctaaagccttctttgagtgtgaggct 00000305
<<<<<<<< |||||||||||||||||||||||||||||||||||||||||||||||||| <<<<<<<<
10881937 cccctacagggtattttctatgtcctaaagccttctttgagtgtgaggct 10881888

00000306 tcaattattgtttttacgaag 00000326
<<<<<<<< ||||||||||||||||||||| <<<<<<<<
10881887 tcaattattgtttttacgaag 10881867
```

Alignment of Clone 2 and chr16:69131743-69132092

Click on links in the frame to the left to navigate through the alignment. Matching bases in cDNA and genomic sequences are colored blue and capitalized. Light blue bases mark the boundaries of gaps in either sequence (often splice sites).

Clone 2

```
GGCGTGTGCC ACCGTGCCCA GCCGAGCCTT CTTAGCTTTT TGGGGTTTTT  50
TTTGGTTGTT TGTTTGTTTA AGACAGAGTC TTACTCTGTT TCCCAGGATG  100
CTGGAGTGCA GGGGTGCAAT CTCGGCTCAC TGCATCCTCG ACCTCCCAGG  150
CTCAAGGTGA TCCTTCCATC TCAGCCTCCC AAGTAGCTGG GACTATTAGC  200
AAGGTGCATG CCTGGCTAAT ATATGTATTT CTTGTAAAGT CTCAAACTTC  250
TGGACTTAAG CAATCCAAGC AATCCACCTG CCTCAGTCTC CCAATGTGCT  300
GAGATTACAG GCTTGAGCCA TAGCGCCTGG CTGATATTTG TCCCTTTGTG  350
```

Genomic chr16 :

```
acaaaatgag ctggtctcaa accagctggc caagctggtc tcaaactcct  69131692
gacctcaagt gatccatcca ccttggcctt cccgaagtgt tgggactaca  69131742
GGCGTGTGCC ACCGTGCCCA GCCGAGCCTT CTTAGCTTTT TGGGGTTTTT  69131792
TTTGGTTGTT TGTTTGTTTA AGACAGAGTC TTACTCTGTT TCCCAGGATG  69131842
CTGGAGTGCA GGGGTGCAAT CTCGGCTCAC TGCATCCTCG ACCTCCCAGG  69131892
CTCAAGGTGA TCCTTCCATC TCAGCCTCCC AAGTAGCTGG GACTATTAGC  69131942
AAGGTGCATG CCTGGCTAAT ATATGTATTT CTTGTAAAGT CTCAAACTTC  69131992
TGGACTTAAG CAATCCAAGC AATCCACCTG CCTCAGTCTC CCAATGTGCT  69132042
GAGATTACAG GCTTGAGCCA TAGCGCCTGG CTGATATTTG TCCCTTTGTG  69132092
tctagctcat tttacttggc ataatattta gaaaattcat gtttaaaaaa  69132142
aaaaagaaag aaaaagattc atgttttggt agcctgtatc agagcttcgt  69132192
```

Side by Side Alignment

```
00000001 ggcgtgtgccaccgtgcccagccgagccttcttagcttttgggg tttttt 00000050
```

```
00000001 ggcgtgtgccaccgtgcccagccgagccttcttagcttttggggtttttt 00000050
>>>>>>>> |||||||||||||||||||||||||||||||||||||||||||||||||| >>>>>>>>
69131743 ggcgtgtgccaccgtgcccagccgagccttcttagcttttggggtttttt 69131792

00000051 tttggttgtttgtttgtttaagacagagtcttactctgtttcccaggatg 00000100
>>>>>>>> |||||||||||||||||||||||||||||||||||||||||||||||||| >>>>>>>>
69131793 tttggttgtttgtttgtttaagacagagtcttactctgtttcccaggatg 69131842

00000101 ctggagtgcaggggtgcaatctcggctcactgcatcctcgacctcccagg 00000150
>>>>>>>> |||||||||||||||||||||||||||||||||||||||||||||||||| >>>>>>>>
69131843 ctggagtgcaggggtgcaatctcggctcactgcatcctcgacctcccagg 69131892

00000151 ctcaaggtgatccttccatctcagcctcccaagtagctgggactattagc 00000200
>>>>>>>> |||||||||||||||||||||||||||||||||||||||||||||||||| >>>>>>>>
69131893 ctcaaggtgatccttccatctcagcctcccaagtagctgggactattagc 69131942
```

```
00000201 aaggtgcatgcctggctaatatatgtatttcttgtaaagtctcaaacttc 00000250
>>>>>>>> |||||||||||||||||||||||||||||||||||||||||||||||||| >>>>>>>>
69131943 aaggtgcatgcctggctaatatatgtatttcttgtaaagtctcaaacttc 69131992

00000251 tggacttaagcaatccaagcaatccacctgcctcagtctcccaatgtgct 00000300
>>>>>>>> |||||||||||||||||||||||||||||||||||||||||||||||||| >>>>>>>>
69131993 tggacttaagcaatccaagcaatccacctgcctcagtctcccaatgtgct 69132042

00000301 gagattacaggcttgagccatagcgcctggctgatatttgtccctttgtg 00000350
>>>>>>>> |||||||||||||||||||||||||||||||||||||||||||||||||| >>>>>>>>
69132043 gagattacaggcttgagccatagcgcctggctgatatttgtccctttgtg 69132092
```

Alignment of Clone 3 and chr19:38266193-38266854

Click on links in the frame to the left to navigate through the alignment. Matching bases in cDNA and genomic sequences are colored blue and capitalized. Light blue bases mark the boundaries of gaps in either sequence (often splice sites).

Clone 3

```
TGCAGTGttG tGATCcCGGC TCACTGCAgc cttgACCTCC tgaGTTCAAG  50
tGgTcCTCCT GCCTaAGTCT CAAGagttcc tgggaccgta ggtgttaatt  100
aaaaaTAATT TTTTTTTtGT AGAGAtgGGG TcTCGCCATA TTGcCCAGGC  150
TGGTCTTGAt ctcttggctt caaGTGATCc tCCTGCCTCG GCCTCCCAAA  200
GgaCTGGGAT TACAGGCGTG AGCCAttgTG CCCAtC
```

Genomic chr19 :

```
gttggaatta taggcgtgac tctctacgcc tggccccaac catgttttg  38266142
tttttttttt ttttctgtga cagagtcacg ctctgttgtc caggctggag  38266192
TGCAGTGgcG cGATCtCGGC TCACTGCAAC CTCCgccttc cagGTTCAAG  38266242
cGaTtCTCCT GCCTcAGccT CTCAAGtagc tgggattata ggtacattcc  38266292
actacgcctg gcTAATTTTT gtatTTTTaG TAGAGAcaGG GTtTCGCCAT  38266342
ATTGgCCAGG CTGGTCTTGA agtccttagg tcaagcaatc catctgcctt  38266392
cacctcccaa agtgttggaa ttacaggcgt gagccattgc atgtggcccc  38266442
caaccttgct tttgaagtgg aaggaaagga catggatttg tagactttgt  38266492
ttttctggac taaaaacatt ctttaacttc tcagtaatac aagtttttga  38266542
ggattgattc attgtcttct aagcactttt ttttgagagt ttcgctcttg  38266592
tcacccaggc tggcatgcaa tggtgcgatc tcggctcact acaacctctg  38266642
cctcccaggt tcaagagatt ctccagcctc agcctcccga gtggctggga  38266692
ttataggtgc ccaccaccat gcccggctaa tttttatatt tttagtagac  38266742
acagggtttt accacgttgg ccaggctgga ctcgaacttc tgacctcagG  38266792
TGATCtgCCT GCCTCGGCCT CCCAAAGtgC TGGGATTACA GGCGTGAGCC  38266842
AccaTGCCCA gCcagttttc taagcacttt gaaatttaaa tgcactctgt  38266892
ctgtatttta cgatataaca cactagacta ataattattt ttactccctg  38266942
ctttgaagtt tt
```

Side by Side Alignment

```
00000001 tgcagtgttgtgatcccggctcactgcagccttgacctcc 00000040
>>>>>>>> |||||||  | |||| ||||||||||||      |||||| >>>>>>>>
38266193 tgcagtggcgcgatctcggctcactgca......acctcc 38266226
```

```
00000044 gttcaagtggtcctcctgcctaag..tctcaag 00000074
>>>>>>>> ||||||| | | ||||||||| ||  ||||||| >>>>>>>>
38266236 gttcaagcgattctcctgcctcagcctctcaag 38266268
```

```
00000106 taatttt....tttttgtagagatggggtctcgccatattgcccaggct 00000151
```

```
>>>>>>>> ||||||||    |||| |||||||  |||| ||||||||||| ||||||| >>>>>>>>
38266305 taattttgtatttttagtagagacagggtttcgccatattggccaggct 38266354

00000152 ggtcttga 00000159
>>>>>>>> |||||||| >>>>>>>>
38266355 ggtcttga 38266362
```

```
00000174 gtgatcctcctgcctcggcctcccaaaggactgggattacaggcgtgagc 00000223
>>>>>>>> ||||||  ||||||||||||||||||||  |||||||||||||||||||| >>>>>>>>
38266792 gtgatctgcctgcctcggcctcccaaagtgctgggattacaggcgtgagc 38266841

00000224 cattgtgcccatc 00000236
>>>>>>>> ||   |||||| | >>>>>>>>
38266842 caccatgcccagc 38266854
```

Alignment of Clone 4 and chr22:49140788-49141189

Click on links in the frame to the left to navigate through the alignment. Matching bases in cDNA and genomic sequences are colored blue and capitalized. Light blue bases mark the boundaries of gaps in either sequence (often splice sites).

Clone 4

```
TGCTGGGATT ACAccCcTGA GCCACTGCGC CCGGCCTTTT TTTTTTGAGA  50
TAGAGTCTCG CTCTGTCGCC CAGGCTGGAG TGCAGTGGTc CGATCTTGGC  100
TCACTGCAAG CTCCGCCTCC TGGATTCACG CCATTCTCCT GCCTCAGCCT  150
CCTGAGTACC TGGGACTACA GGTGCCCGCC ACCACACCCA GCTAATTTTT  200
TTCTATTTTT AGTAGAGACG GGGTTTCACT GTGTTAGCCA GGATGGTCTC  250
GATCTCCTTA CCTCATGATC CGCCCGCCTC GGCCTCCCAA ATTGCTGGGA  300
TTACAGGGGT GAGCCACTGC GCCTGGCACA GCCTTATTCT TTAAAATGGA  350
AAAAAAAAAA aCAAAAAAAA AGCTTTTAAA AATTTTTCTC TTTTAAAAAG  400
TG
```

Genomic chr22 :

```
cacgcccggc taattttttg tatttttagt agagaagggg tttcactgtg  49140737
gtctcgatct cctaacctcg tgatcggccg cccgccttgg ccttccaaag  49140787
TGCTGGGATT ACAagCgTGA GCCACTGCGC CCGGCCTTTT TTTTTTGAGA  49140837
TAGAGTCTCG CTCTGTCGCC CAGGCTGGAG TGCAGTGGTg CGATCTTGGC  49140887
TCACTGCAAG CTCCGCCTCC TGGATTCACG CCATTCTCCT GCCTCAGCCT  49140937
CCTGAGTACC TGGGACTACA GGTGCCCGCC ACCACACCCA GCTAATTTTT  49140987
TTCTATTTTT AGTAGAGACG GGGTTTCACT GTGTTAGCCA GGATGGTCTC  49141037
GATCTCCTTA CCTCATGATC CGCCCGCCTC GGCCTCCCAA ATTGCTGGGA  49141087
TTACAGGGGT GAGCCACTGC GCCTGGCACA GCCTTATTCT TTAAAATGGA  49141137
AAAAAAAAAA cCAAAAAAAA AGCTTTTAAA AATTTTTCTC TTTTAAAAAG  49141187
TGtacacata acacataatt gtatatattt atggggtaca tagtgatgtt  49141237
ttaataccta tagtgtattt ccctcttttt tttttttga gacggagtct  49141287
cg
```

Side by Side Alignment

```
00000001 tgctgggattacacccctgagccactgcgcccggcctttttttttttgaga 00000050
>>>>>>>> |||||||||||||  | ||||||||||||||||||||||||||||||||| >>>>>>>>
49140788 tgctgggattacaagcgtgagccactgcgcccggcctttttttttttgaga 49140837

00000051 tagagtctcgctctgtcgcccaggctggagtgcagtggtccgatcttggc 00000100
>>>>>>>> ||||||||||||||||||||||||||||||||||||||| |||||||||| >>>>>>>>
49140838 tagagtctcgctctgtcgcccaggctggagtgcagtggtgcgatcttggc 49140887

00000101 tcactgcaagctccgcctcctggattcacgccattctcctgcctcagcct 00000150
>>>>>>>> |||||||||||||||||||||||||||||||||||||||||||||||||| >>>>>>>>
49140888 tcactgcaagctccgcctcctggattcacgccattctcctgcctcagcct 49140937
```

```
00000151 cctgagtacctgggactacaggtgcccgccaccacacccagctaattttt 00000200
>>>>>>>> |||||||||||||||||||||||||||||||||||||||||||||||||| >>>>>>>>
49140938 cctgagtacctgggactacaggtgcccgccaccacacccagctaattttt 49140987

00000201 ttctatttttagtagagacggggtttcactgtgttagccaggatggtctc 00000250
>>>>>>>> |||||||||||||||||||||||||||||||||||||||||||||||||| >>>>>>>>
49140988 ttctatttttagtagagacggggtttcactgtgttagccaggatggtctc 49141037

00000251 gatctccttacctcatgatccgcccgcctcggcctcccaaattgctggga 00000300
>>>>>>>> |||||||||||||||||||||||||||||||||||||||||||||||||| >>>>>>>>
49141038 gatctccttacctcatgatccgcccgcctcggcctcccaaattgctggga 49141087

00000301 ttacaggggtgagccactgcgcctggcacagccttattctttaaaatgga 00000350
>>>>>>>> |||||||||||||||||||||||||||||||||||||||||||||||||| >>>>>>>>
49141088 ttacaggggtgagccactgcgcctggcacagccttattctttaaaatgga 49141137

00000351 aaaaaaaaaaacaaaaaaaaagcttttaaaaatttttctcttttaaaaag 00000400
>>>>>>>> |||||||||| ||||||||||||||||||||||||||||||||||||||| >>>>>>>>
49141138 aaaaaaaaaaccaaaaaaaaagcttttaaaaatttttctcttttaaaaag 49141187

00000401 tg 00000402
>>>>>>>> || >>>>>>>>
49141188 tg 49141189
```

Alignment of Clone 5 and chr3:126280985-126281230

Click on links in the frame to the left to navigate through the alignment. Matching bases in cDNA and genomic sequences are colored blue and capitalized. Light blue bases mark the boundaries of gaps in either sequence (often splice sites).

Clone 5

```
AGCCTCCCAA GTAGCTGGGA GTGCACCAGC GTACCCAGCT AATCTTTGTA  50
ATTTTTGTAA AGATGGGATT TTGCCATGTT GTCATGCTGG TCTCGAATTT  100
CTAGGCTCAA GTAGTCCTCC TGCCTCAGCC TCACAAAGTG CTGGGATTAC  150
AGGTATGAGC CACCCCACCC GGCCATGACT AATCTTAATA TGTCTATTTT  200
TGCTCATTGT CACTGAGTGG ACCTGGAGGC TGAGGGCTGG AAGAGC
```

Genomic chr3 (reverse strand):

```
tgaaacatga tcttactctg tcacccaggc tggagtacag tggtgcaatc  126281281
acagctcact gcagcctcaa cctcctgggc tccagccatc ctcccacctc  126281231
AGCCTCCCAA GTAGCTGGGA GTGCACCAGC GTACCCAGCT AATCTTTGTA  126281181
ATTTTTGTAA AGATGGGATT TTGCCATGTT GTCATGCTGG TCTCGAATTT  126281131
CTAGGCTCAA GTAGTCCTCC TGCCTCAGCC TCACAAAGTG CTGGGATTAC  126281081
AGGTATGAGC CACCCCACCC GGCCATGACT AATCTTAATA TGTCTATTTT  126281031
TGCTCATTGT CACTGAGTGG ACCTGGAGGC TGAGGGCTGG AAGAGCaact  126280981
ggaattgctg tagtgccaaa tatatcccct ttcatgttga tgccaccctg  126280931
gccaaggtat catttttgtg tggcagccag agaaatcttt tcaaaa
```

Side by Side Alignment

```
000000001 agcctcccaagtagctgggagtgcaccagcgtacccagctaatctttgta 000000050
<<<<<<<<< |||||||||||||||||||||||||||||||||||||||||||||||||| <<<<<<<<<
126281230 agcctcccaagtagctgggagtgcaccagcgtacccagctaatctttgta 126281181

000000051 atttttgtaaagatgggattttgccatgttgtcatgctggtctcgaattt 000000100
<<<<<<<<< |||||||||||||||||||||||||||||||||||||||||||||||||| <<<<<<<<<
126281180 atttttgtaaagatgggattttgccatgttgtcatgctggtctcgaattt 126281131

000000101 ctaggctcaagtagtcctcctgcctcagcctcacaaagtgctgggattac 000000150
<<<<<<<<< |||||||||||||||||||||||||||||||||||||||||||||||||| <<<<<<<<<
126281130 ctaggctcaagtagtcctcctgcctcagcctcacaaagtgctgggattac 126281081

000000151 aggtatgagccaccccacccggccatgactaatcttaatatgtctatttt 000000200
<<<<<<<<< |||||||||||||||||||||||||||||||||||||||||||||||||| <<<<<<<<<
126281080 aggtatgagccaccccacccggccatgactaatcttaatatgtctatttt 126281031

000000201 tgctcattgtcactgagtggacctggaggctgagggctggaagagc 000000246
<<<<<<<<< |||||||||||||||||||||||||||||||||||||||||||||| <<<<<<<<<
126281030 tgctcattgtcactgagtggacctggaggctgagggctggaagagc 126280985
```

Alignment of Clone 6 and chr2:113102773-113103105

Click on links in the frame to the left to navigate through the alignment. Matching bases in cDNA and genomic sequences are colored blue and capitalized. Light blue bases mark the boundaries of gaps in either sequence (often splice sites).

Clone 6

```
AAAAGAAAAA AACAGAGATG GATGAGATTA GCTACCTGCA GGGCATGGTA  50
GGAACAGGAT GGAAATAAAG GGAATGAGGG GGAATGAGAC TTCTAGGAAT  100
ATCTCTTTTT GTTTATTTTA TTTTATTTTT AGCAGAGATG TGGGTTCCTT  150
ATGTTGCCCA GCTGGTCTTG AACTCCTGGG CTCCAGCTAT CCTCCTGCCT  200
CCATCTCCCA AAGTGCTGGG ATTACAGGTG TGAGCTACCG CACCCGATCC  250
TCTTTTTGTT CTGAACTGAC TTTTAAAATC ATTTTAATAT TTCACATCTC  300
AAAAATAAAT ACAAATAAAA CTCACAAGGA CAGaattcag at
```

Genomic chr2 :

```
tcgtgatcca cctgccttgg cctcccaaaa tgctgggatt acaggcgtga  113102722
gccaccaggc cggccacatt tacctatttg ttcaaggaaa aagaaacaga  113102772
AAAAGAAAAA AACAGAGATG GATGAGATTA GCTACCTGCA GGGCATGGTA  113102822
GGAACAGGAT GGAAATAAAG GGAATGAGGG GGAATGAGAC TTCTAGGAAT  113102872
ATCTCTTTTT GTTTATTTTA TTTTATTTTT AGCAGAGATG TGGGTTCCTT  113102922
ATGTTGCCCA GCTGGTCTTG AACTCCTGGG CTCCAGCTAT CCTCCTGCCT  113102972
CCATCTCCCA AAGTGCTGGG ATTACAGGTG TGAGCTACCG CACCCGATCC  113103022
TCTTTTTGTT CTGAACTGAC TTTTAAAATC ATTTTAATAT TTCACATCTC  113103072
AAAAATAAAT ACAAATAAAA CTCACAAGGA CAGggaaaac gctaaagttg  113103122
aacacaagca gaaacaagca aataaaatgt accctttgac ttctatctta  113103172
ccactttgta gtaaaaataa ctaatcccag taa
```

Side by Side Alignment

```
000000001 aaaagaaaaaaacagagatggatgagattagctacctgcagggcatggta 000000050
>>>>>>>>> |||||||||||||||||||||||||||||||||||||||||||||||||| >>>>>>>>>
113102773 aaaagaaaaaaacagagatggatgagattagctacctgcagggcatggta 113102822

000000051 ggaacaggatggaaataaagggaatgagggggaatgagacttctaggaat 000000100
>>>>>>>>> |||||||||||||||||||||||||||||||||||||||||||||||||| >>>>>>>>>
113102823 ggaacaggatggaaataaagggaatgagggggaatgagacttctaggaat 113102872

000000101 atctctttttgtttattttattttatttttagcagagatgtgggttcctt 000000150
>>>>>>>>> |||||||||||||||||||||||||||||||||||||||||||||||||| >>>>>>>>>
113102873 atctctttttgtttattttattttatttttagcagagatgtgggttcctt 113102922

000000151 atgttgcccagctggtcttgaactcctgggctccagctatcctcctgcct 000000200
>>>>>>>>> |||||||||||||||||||||||||||||||||||||||||||||||||| >>>>>>>>>
113102923 atgttgcccagctggtcttgaactcctgggctccagctatcctcctgcct 113102972
```

```
000000201 ccatctcccaaagtgctgggattacaggtgtgagctaccgcacccgatcc 000000250
>>>>>>>>> |||||||||||||||||||||||||||||||||||||||||||||||||| >>>>>>>>>
113102973 ccatctcccaaagtgctgggattacaggtgtgagctaccgcacccgatcc 113103022

000000251 tctttttgttctgaactgacttttaaaatcattttaatatttcacatctc 000000300
>>>>>>>>> |||||||||||||||||||||||||||||||||||||||||||||||||| >>>>>>>>>
113103023 tctttttgttctgaactgacttttaaaatcattttaatatttcacatctc 113103072

000000301 aaaaataaatacaaataaaactcacaaggacag 000000333
>>>>>>>>> ||||||||||||||||||||||||||||||||| >>>>>>>>>
113103073 aaaaataaatacaaataaaactcacaaggacag 113103105
```

Alignment of Clone 7 and chr2:113102883-113103072

Click on links in the frame to the left to navigate through the alignment. Matching bases in cDNA and genomic sequences are colored blue and capitalized. Light blue bases mark the boundaries of gaps in either sequence (often splice sites).

Clone 7

```
GTTTATTTTA TTTTATTTTT AGCAGAGATG TGGGTTCCTT ATGTTGCCCA  50
GCTGGTCTTG AACTCCTGGG CTCCAGCTAT CCTCCTGCCT CCATCTCCCA  100
AAGTGCTGGG ATTACAGGTG TGAGCTACCG CACCCGATCC TCTTTTTGTT  150
CTGAACTGAC TTTTAAAATC ATTTTAATAT TTCACATCTC
```

Genomic chr2 :

```
aacagagatg gatgagatta gctacctgca gggcatggta ggaacaggat  113102832
ggaaataaag ggaatgaggg ggaatgagac ttctaggaat atctcttttt  113102882
GTTTATTTTA TTTTATTTTT AGCAGAGATG TGGGTTCCTT ATGTTGCCCA  113102932
GCTGGTCTTG AACTCCTGGG CTCCAGCTAT CCTCCTGCCT CCATCTCCCA  113102982
AAGTGCTGGG ATTACAGGTG TGAGCTACCG CACCCGATCC TCTTTTTGTT  113103032
CTGAACTGAC TTTTAAAATC ATTTTAATAT TTCACATCTC aaaaataaat  113103082
acaaataaaa ctcacaagga cagggaaaac gctaaagttg aacacaagca  113103132
gaaacaagca aataaaatgt accctttgac ttctatctta
```

Side by Side Alignment

```
000000001 gtttattttattttatttttagcagagatgtgggttccttatgttgccca 000000050
>>>>>>>>> |||||||||||||||||||||||||||||||||||||||||||||||||| >>>>>>>>>
113102883 gtttattttattttatttttagcagagatgtgggttccttatgttgccca 113102932

000000051 gctggtcttgaactcctgggctccagctatcctcctgcctccatctccca 000000100
>>>>>>>>> |||||||||||||||||||||||||||||||||||||||||||||||||| >>>>>>>>>
113102933 gctggtcttgaactcctgggctccagctatcctcctgcctccatctccca 113102982

000000101 aagtgctgggattacaggtgtgagctaccgcacccgatcctctttttgtt 000000150
>>>>>>>>> |||||||||||||||||||||||||||||||||||||||||||||||||| >>>>>>>>>
113102983 aagtgctgggattacaggtgtgagctaccgcacccgatcctctttttgtt 113103032

000000151 ctgaactgacttttaaaatcattttaatatttcacatctc 000000190
>>>>>>>>> |||||||||||||||||||||||||||||||||||||||| >>>>>>>>>
113103033 ctgaactgacttttaaaatcattttaatatttcacatctc 113103072
```

Alignment of Clone 8 and chr16:45169084-45169213

Click on links in the frame to the left to navigate through the alignment. Matching bases in cDNA and genomic sequences are colored blue and capitalized. Light blue bases mark the boundaries of gaps in either sequence (often splice sites).

Clone 8

```
CATGTTGGCC AGGCTGGTCT CGACCTCCTG ACCTTAGGTG ATCCACCTGC  50
CTCGGCCTCC CAAAGTGCTG GGATTATAGG CATGAGCCAC TGTGCCTGGC  100
CTGTGATTTT TTTTTTTAAG AGACAGGGCC
```

Genomic chr16 :

```
aagtgattct cctgccacag cctccctact agctgggatt acaggcgcct  45169033
gccaccatgc ctggctaatt tttgtatttt tagtagagac agggtttcac  45169083
CATGTTGGCC AGGCTGGTCT CGACCTCCTG ACCTTAGGTG ATCCACCTGC  45169133
CTCGGCCTCC CAAAGTGCTG GGATTATAGG CATGAGCCAC TGTGCCTGGC  45169183
CTGTGATTTT TTTTTTTAAG AGACAGGGCC tcactctgtc acccaggctg  45169233
gagtgcagtg gtgcaatcat agctcactgc agcctgtaac tcctgggttc  45169283
tagcaatcct cctacttcta cctcctgagt
```

Side by Side Alignment

```
00000001 catgttggccaggctggtctcgacctcctgaccttaggtgatccacctgc 00000050
>>>>>>>> |||||||||||||||||||||||||||||||||||||||||||||||||| >>>>>>>>
45169084 catgttggccaggctggtctcgacctcctgaccttaggtgatccacctgc 45169133

00000051 ctcggcctcccaaagtgctgggattataggcatgagccactgtgcctggc 00000100
>>>>>>>> |||||||||||||||||||||||||||||||||||||||||||||||||| >>>>>>>>
45169134 ctcggcctcccaaagtgctgggattataggcatgagccactgtgcctggc 45169183

00000101 ctgtgattttttttttttaagagacagggcc 00000130
>>>>>>>> |||||||||||||||||||||||||||||| >>>>>>>>
45169184 ctgtgattttttttttttaagagacagggcc 45169213
```

Alignment of Clone 9 and chr16:45169083-45169213

Click on links in the frame to the left to navigate through the alignment. Matching bases in cDNA and genomic sequences are colored blue and capitalized. Light blue bases mark the boundaries of gaps in either sequence (often splice sites).

Clone 9

```
aagctggaan ctccaccggc gggtgggcgg gcccgcttct taagccccgg  50
cgggtgaccc gggagtatct gaattCCATG TTGGCCAGGC TGGTCTCGAC  100
CTCCTGACCT TAGGTGATCC ACCTGCCTCG GCCTCCCAAA GTGCTGGGAT  150
TATAGGCATG AGCCACTGTG CCTGGCCTGT GATTTTTTTT TTTAAGAGAC  200
AGGGCC
```

Genomic chr16 :

```
caagtgattc tcctgccaca gcctccctac tagctgggat tacaggcgcc  45169032
tgccaccatg cctggctaat ttttgtattt ttagtagaga cagggtttca  45169082
CCATGTTGGC CAGGCTGGTC TCGACCTCCT GACCTTAGGT GATCCACCTG  45169132
CCTCGGCCTC CCAAAGTGCT GGGATTATAG GCATGAGCCA CTGTGCCTGG  45169182
CCTGTGATTT TTTTTTTTAA GAGACAGGGC Ctcactctgt cacccaggct  45169232
ggagtgcagt ggtgcaatca tagctcactg cagcctgtaa ctcctgggtt  45169282
ctagcaatcc tcctacttct acctcctgag t
```

Side by Side Alignment

```
00000076 ccatgttggccaggctggtctcgacctcctgaccttaggtgatccacctg 00000125
>>>>>>>> |||||||||||||||||||||||||||||||||||||||||||||||||| >>>>>>>>
45169083 ccatgttggccaggctggtctcgacctcctgaccttaggtgatccacctg 45169132

00000126 cctcggcctcccaaagtgctgggattataggcatgagccactgtgcctgg 00000175
>>>>>>>> |||||||||||||||||||||||||||||||||||||||||||||||||| >>>>>>>>
45169133 cctcggcctcccaaagtgctgggattataggcatgagccactgtgcctgg 45169182

00000176 cctgtgatttttttttttaagagacagggcc 00000206
>>>>>>>> ||||||||||||||||||||||||||||||| >>>>>>>>
45169183 cctgtgatttttttttttaagagacagggcc 45169213
```

Alignment of Clone 10 and chr11:91127838-91128028

Click on links in the frame to the left to navigate through the alignment. Matching bases in cDNA and genomic sequences are colored blue and capitalized. Light blue bases mark the boundaries of gaps in either sequence (often splice sites).

Clone 10

```
AGCCTCCCAA AGTGCTGGGA TTACAGGTGT GAGCCACCAT GCCCCAGCCT  50
CCTTTCCTTT TGAATACTAC AAAATACCCT TTTCCAAGCT ATCTCTTTTG  100
AGCCCTAAAC TTGTATTCCA ACTTACCTCC TTGACAAATC AGTGGCATCT  150
CAAATTCAAC ATGATGAGCA CTGAATTTGT CTTCCACTTA A
```

Genomic chr11 :

```
ccaccatgcc cagctaattt ttgtattttt tgtagagatg gggttttacc  91127787
atgttggcca ggatggcctc gatcacttga cctcgtgatc cgcctgcctc  91127837
AGCCTCCCAA AGTGCTGGGA TTACAGGTGT GAGCCACCAT GCCCCAGCCT  91127887
CCTTTCCTTT TGAATACTAC AAAATACCCT TTTCCAAGCT ATCTCTTTTG  91127937
AGCCCTAAAC TTGTATTCCA ACTTACCTCC TTGACAAATC AGTGGCATCT  91127987
CAAATTCAAC ATGATGAGCA CTGAATTTGT CTTCCACTTA Aaacctagtt  91128037
ctcattattt tccatctcag aaaggtcagg attatctatc tatcccattg  91128087
cccaagtcat gaatctagaa gttttttaa tttacctttc t
```

Side by Side Alignment

```
00000001 agcctcccaaagtgctgggattacaggtgtgagccaccatgccccagcct 00000050
>>>>>>>> |||||||||||||||||||||||||||||||||||||||||||||||||| >>>>>>>>
91127838 agcctcccaaagtgctgggattacaggtgtgagccaccatgccccagcct 91127887

00000051 cctttccttttgaatactacaaaatacccttttccaagctatctcttttg 00000100
>>>>>>>> |||||||||||||||||||||||||||||||||||||||||||||||||| >>>>>>>>
91127888 cctttccttttgaatactacaaaatacccttttccaagctatctcttttg 91127937

00000101 agccctaaacttgtattccaacttacctccttgacaaatcagtggcatct 00000150
>>>>>>>> |||||||||||||||||||||||||||||||||||||||||||||||||| >>>>>>>>
91127938 agccctaaacttgtattccaacttacctccttgacaaatcagtggcatct 91127987

00000151 caaattcaacatgatgagcactgaatttgtcttccacttaa 00000191
>>>>>>>> ||||||||||||||||||||||||||||||||||||||||| >>>>>>>>
91127988 caaattcaacatgatgagcactgaatttgtcttccacttaa 91128028
```
