# Supplementary material for: Hepatoma derived growth factor binds DNA through the N-terminal PWWP domain
Source: BMC Mol Biol. 2007 Oct 31;8:101. doi: 10.1186/1471-2199-8-101 (PMC2176068; doi:10.1186/1471-2199-8-101)
Supplement: Additional file 3 — Genomic location of ChIP clones. The data shows the chromosome location and alignment of non-specific ChIP clones 11–100 to human genome. [file 1471-2199-8-101-S3.pdf]

Additional Data 3

Genomic location of ChIP clones. Blat program (http://genome.ucsc.edu/) was used to map the location for 100 ChIP clones in human genome (90 non specific clones are shown below; specific clones are shown in Additional Date 2). Matching bases in input DNA and genomic sequences are colored blue and capitalized. Light blue bases mark the boundaries of gaps in either sequence.

Alignment of YourSeq and chr4:48969215-48969486

Clone 11 YourSeq

```
CTCTCCTAGC TTAAATCAGG AAGAATTAGA TACCCCAAGC aGACCAATAA  50
AGCAAGCAGC AAGATTGAAA TGGTAATTTT AAAATTACCA GCAAAAAAAG  100
CTGAGGGCCA GACAGATTCA CAGCAGAGTT CTACCAGACA TTCAAAGAAT  150
GTCTTCTTTC ATTCAAGGAA GAAATGATAC CAATCTTTTC ATACTATTCC  200
ACAAGACAGA GAAAGAAGAA ACCCTCCCTT ATTCATTCTA TGAAGCCAGC  250
ATCACCCTAA TACCAAAACC AT
```

Genomic chr4 :

```
ccactgaaat attaaagatt attcaagggt actatgaaca ccttttggca  48969164
cataaactac aaagcctaga agagttggat aaattcctgg aaaaatacaa  48969214
CTCTCCTAGC TTAAATCAGG AAGAATTAGA TACCCCAAGC cGACCAATAA  48969264
AGCAAGCAGC AAGATTGAAA TGGTAATTTT AAAATTACCA GCAAAAAAAG  48969314
CTGAGGGCCA GACAGATTCA CAGCAGAGTT CTACCAGACA TTCAAAGAAT  48969364
GTCTTCTTTC ATTCAAGGAA GAAATGATAC CAATCTTTTC ATACTATTCC  48969414
ACAAGACAGA GAAAGAAGAA ACCCTCCCTT ATTCATTCTA TGAAGCCAGC  48969464
ATCACCCTAA TACCAAAACC ATggaaggac ataaccaaaa aagaaaacta  48969514
cagaccaata tccttgatga acgcagatgc caaaatcctt aacaaaatac  48969564
tatctaactg aatccgacaa ca
```

Side by Side Alignment

```
00000001 ctctcctagcttaaatcaggaagaattagataccccaagcagaccaataa 00000050
>>>>>>>> ||||||||||||||||||||||||||||||||||||||||| ||||||||| >>>>>>>>
48969215 ctctcctagcttaaatcaggaagaattagataccccaagccgaccaataa 48969264

00000051 agcaagcagcaagattgaaatggtaattttaaaattaccagcaaaaaaag 00000100
>>>>>>>> |||||||||||||||||||||||||||||||||||||||||||||||||| >>>>>>>>
48969265 agcaagcagcaagattgaaatggtaattttaaaattaccagcaaaaaaag 48969314

00000101 ctgagggccagacagattcacagcagagttctaccagacattcaaagaat 00000150
>>>>>>>> |||||||||||||||||||||||||||||||||||||||||||||||||| >>>>>>>>
48969315 ctgagggccagacagattcacagcagagttctaccagacattcaaagaat 48969364

00000151 gtcttctttcattcaaggaagaaatgataccaatcttttcatactattcc 00000200
>>>>>>>> |||||||||||||||||||||||||||||||||||||||||||||||||| >>>>>>>>
48969365 gtcttctttcattcaaggaagaaatgataccaatcttttcatactattcc 48969414

00000201 acaagacagagaaagaagaaaccctcccttattcattctatgaagccagc 00000250
>>>>>>>> |||||||||||||||||||||||||||||||||||||||||||||||||| >>>>>>>>
48969415 acaagacagagaaagaagaaaccctcccttattcattctatgaagccagc 48969464

00000251 atcaccctaataccaaaaccat 00000272
>>>>>>>> |||||||||||||||||||||| >>>>>>>>
48969465 atcaccctaataccaaaaccat 48969486
```

Alignment of YourSeq and chr13:26617176-26617195

Clone 12 YourSeq

```
ccgcgatcca cgcgcgcatg gcccgcggtg aacgtgctga tcggggcggc  50
ggtatcggtc tggacctgat cgcgcggctg tgcgagcaCC TGGGCTGGAC  100
CCTGCAGCtg cagccctgcg acccgcgtgg cacgcgggtc accctggact  150
tcggcgcctc gcgaccgggc tgaccgacat gccaatcgct ccgcgctgcg  200
gcatgacccg gcgtgaagac aatgttaatc tcgacctgcg ccgctttatg  250
cggcg
```

Genomic chr13 :

```
tgcctgtaat cccagctact cagaaggcag aggtgggaga atcacctaag  26617125
cccaggatgt cgaggctgca gtgagccacg atcgcaccac tgcattccag  26617175
CCTGGGCTGG ACCCTGCAGC agagtgagac cctgcagcag aggggacttg  26617225
aacacaagtc ttgggctctg tatcaaatgt agccatccca ctcttcactg  26617275
aacctcctcc tttccttcca
```

Side by Side Alignment

```
00000089 cctgggctggaccctgcagc 00000108
>>>>>>>> |||||||||||||||||||| >>>>>>>>
26617176 cctgggctggaccctgcagc 26617195
```

Alignment of YourSeq and chr15:53425196-53425231

Clone 13 YourSeq

```
cggtgacccg ggagatctga attccancag ggccggatct cgctcgccaa  50
gaccggtgat gtcgatcacc ccgcagaccc ggccgtcgag atcgtgcagt  100
ggctcgctca ggcagaacac cggcgcgaac cgctgcaggt agtgttcgtt  150
gccgcgcacc agtgccggca cgtcGTCAgC CAGGCTGacc gccggcgcgG  200
TGGTGCCGAT CTCGGtctcg cccagctgtc gcccgacctg aattcagatc  250
tcccgg
```

Genomic chr15 (reverse strand):

```
ctgggaaaga gagactttga actactaacc ataatgaggg tactgggcac  53425282
agatgaaggt atcaagaaga attttttttt tttagacaga atctcgctct  53425232
GTCAcCCAGG CTGgagtgca GTGGTGCCGA TCTCGGctca atgcaacctc  53425182
cacctccagg gttcaagtga ttctcctgtc tcagcctccc aagtagctgg  53425132
cattacaggt gcgtgccacc acacccagct aatttt
```

Side by Side Alignment

```
00000175 gtcagccaggctg 00000187
<<<<<<<< |||| |||||||| <<<<<<<<
53425231 gtcacccaggctg 53425219
```

```
00000200 gtggtgccgatctcgg 00000215
<<<<<<<< |||||||||||||||| <<<<<<<<
53425211 gtggtgccgatctcgg 53425196
```

Alignment of YourSeq and chr22:48603526-48603551

Clone 14 YourSeq

```
cgatgagacg atcggcctgc ttgacgaagc tctctggggt gtgaatcggg  50
gaagtggtgt agcagatggt gacctgggcg tgcttgccgg tcttcttgac  100
ggctgccatc gcgtgctcaa ggttgcgagg atcgttcaga gcgtcgaaca  150
cccggaacac gtccatgccg ttCTCGGCCG ACTTCTCGAc aAACTTGTcg  200
acgacctcgt cgttgtagtg gcggtagccc agaaggtttt ggc
```

Genomic chr22 :

```
acacggcggg gacgcagtcg cccttgcgct tctcattgac gatctccagc  48603475
caggcatagt cctcctcgtc catgtcatac tccacctcgt tgtccagttc  48603525
CTCGGCCGAC TTCTCGAtgA ACTTGTagta cacaggaggc ctcctggggg  48603575
cggacggagg gctgtactcc acgatgcgca ccttgggctc cgggagggca  48603625
ctggccgagg ccggcgtgcc gtgggc
```

Side by Side Alignment

```
00000173 ctcggccgacttctcgacaaacttgt 00000198
>>>>>>>> ||||||||||||||||  ||||||| >>>>>>>>
48603526 ctcggccgacttctcgatgaacttgt 48603551
```

Alignment of YourSeq and chr5:53384849-53385118

Clone 15 YourSeq

```
cggtgacccg ggagatctga aTTCCACTCT AGAGCCACTC TTTTTTATTT  50
TTATTTTTTT TCTTTTTTCA TACTCTACTG TTTGGAAACA GGTCATACTT  100
AGAGAGCCAT ACTTAGAGAG TAGTTATGCT TTACTTTCTT TTGGATAGAA  150
TACCTACACA AATTATTTGG AATTATTTGG AATTTTTCTA CATAGTAGGT  200
TTATCTCTTC TACCCCATTT ATTTGTTGAT TTATTCAATT ATTTACTTAT  250
GTCAGTAGAG ACTCATGGAT ATTTATTTTA TACTTTGTTT Tgaattcaga  300
tctcccgggt caccg
```

Genomic chr5 (reverse strand):

```
cacatcatat caagggtaaa cactgtcaat gtgacttatc actgttgata  53385169
taaactttta tcacctgctt gaggtagtgt ttgtcaggtt tctccacttt  53385119
TTCCACTCTA GAGCCACTCT TTTTTATTTT TATTTTTTTT CTTTTTTCAT  53385069
ACTCTACTGT TTGGAAACAG GTCATACTTA GAGAGCCATA CTTAGAGAGT  53385019
AGTTATGCTT TACTTTCTTT TGGATAGAAT ACCTACACAA ATTATTTGGA  53384969
ATTATTTGGA ATTTTTCTAC ATAGTAGGTT TATCTCTTCT ACCCCATTTA  53384919
TTTGTTGATT TATTCAATTA TTTACTTATG TCAGTAGAGA CTCATGGATA  53384869
TTTATTTTAT ACTTTGTTTT ttgtttgttt tgttgttgtt tgttgttgtt  53384819
gttgtttttg gagatgcagt tttgccatat tgctcctagt ctccagggat  53384769
cctcccgggc cccagggatc
```

Side by Side Alignment

```
00000022 ttccactctagagccactctttttttatttttatttttttttctttttttcat 00000071
<<<<<<<< |||||||||||||||||||||||||||||||||||||||||||||||||| <<<<<<<<
53385118 ttccactctagagccactctttttttatttttatttttttttctttttttcat 53385069

00000072 actctactgtttggaaacaggtcatacttagagagccatacttagagagt 00000121
<<<<<<<< |||||||||||||||||||||||||||||||||||||||||||||||||| <<<<<<<<
53385068 actctactgtttggaaacaggtcatacttagagagccatacttagagagt 53385019

00000122 agttatgctttactttcttttggatagaatacctacacaaattatttgga 00000171
<<<<<<<< |||||||||||||||||||||||||||||||||||||||||||||||||| <<<<<<<<
53385018 agttatgctttactttcttttggatagaatacctacacaaattatttgga 53384969

00000172 attatttggaatttttctacatagtaggtttatctcttctaccccattta 00000221
<<<<<<<< |||||||||||||||||||||||||||||||||||||||||||||||||| <<<<<<<<
53384968 attatttggaatttttctacatagtaggtttatctcttctaccccattta 53384919

00000222 tttgttgatttattcaattatttacttatgtcagtagagactcatggata 00000271
<<<<<<<< |||||||||||||||||||||||||||||||||||||||||||||||||| <<<<<<<<
53384918 tttgttgatttattcaattatttacttatgtcagtagagactcatggata 53384869

00000272 tttattttatactttgtttt 00000291
<<<<<<<< |||||||||||||||||||| <<<<<<<<
53384868 tttattttatactttgtttt 53384849
```

Alignment of YourSeq and chr4:49219295-49219566

Clone 16 YourSeq

```
ATGGTTTTGG TATaTAGGGT nATGCTaGGC TTCATAGcaa tagacatata  50
gagaGAGGGT TTCTTCnTTC TCTGgCTTGT GnAATAaTAT GAAAAGATTG  100
GTATCATTTC TTCCTTGAAT GAAAGAAGAC ATTCTTTGAA TGTCTGGTAG  150
AacTCTGCTG TGAATCTGTC nGGCnCTCna CTTTTTTTGC TGGTAAnTTT  200
AAAATTACCA TTTCAATCnT GnTGCTTGCT TTAaTGnTCT GCTTgnGGTA  250
TCTAATTCTT CCTGATTTAA GCTAGGAGAG
```

Genomic chr4 :

```
tgttgtcgga ttcagttaga tagtattttg ttaaggattt tggcatctgc  49219244
gttcatcaaa gatattggtc tgtagttttc ttttttggtt atgtccttcc  49219294
ATGGTTTTGG TATTAGGGTg ATGCTGGCTT CATAGaatga ataaggGAGG  49219344
GTTTCTTCtT TCTCTGtCTT GTGgAATAgT ATGAAAAGAT TGGTATCATT  49219394
TCTTCCTTGA ATGAAAGAAG ACATTCTTTG AATGTCTGGT AGAttTCTGC  49219444
TGTGAATCTG TCtGGCcCTC agCTTTTTTT GCTGGTAAtT TTAAAATTAC  49219494
CATTTCAATC tTGcTGCTTG CTTTAtTGgT CTGCTTagGG TATCTAATTC  49219544
TTCCTGATTT AAGCTAGGAG AGttgtattt ttccaggaat ttatccaact  49219594
cttctaggtt ttgtagttta tgtgccaaaa ggtgttcaca gtacccttga  49219644
ataatcttta atatttcagt gg
```

Side by Side Alignment

```
00000001 atggttttggtatatagggtnatgctaggcttcatag 00000037
>>>>>>>> ||||||||||||| |||||| ||||| |||||||||| >>>>>>>>
49219295 atggttttggtat.tagggtgatgct.ggcttcatag 49219329
```

```
00000055 gagggtttcttcnttctctggcttgtgnaataatatgaaaagattggtat 00000104
>>>>>>>> |||||||||||| ||||||| |||||| |||| |||||||||||||||| >>>>>>>>
49219341 gagggtttcttctttctctgtcttgtggaatagtatgaaaagattggtat 49219390

00000105 catttcttccttgaatgaaagaagacattctttgaatgtctggtagaact 00000154
>>>>>>>> |||||||||||||||||||||||||||||||||||||||||||||||  | >>>>>>>>
49219391 catttcttccttgaatgaaagaagacattctttgaatgtctggtagattt 49219440

00000155 ctgctgtgaatctgtcnggcnctcnactttttttgctggtaantttaaaa 00000204
>>>>>>>> |||||||||||||||| ||| |||  |||||||||||||||| ||||||| >>>>>>>>
49219441 ctgctgtgaatctgtctggccctcagctttttttgctggtaattttaaaa 49219490

00000205 ttaccatttcaatcntgntgcttgctttaatgntctgcttgnggtatcta 00000254
>>>>>>>> |||||||||||||| || ||||||||||| || |||||||  |||||||| >>>>>>>>
49219491 ttaccatttcaatcttgctgcttgctttattggtctgcttagggtatcta 49219540

00000255 attcttcctgatttaagctaggagag 00000280
>>>>>>>> |||||||||||||||||||||||||| >>>>>>>>
49219541 attcttcctgatttaagctaggagag 49219566
```

Alignment of YourSeq and chr7:8110887-8111075

Clone 17 YourSeq

```
AAGACTGGGT AATTTATAAA GAAATGAGGT TTAATTGACT CACAGTTCCT  50
CATGGCTGGG GAGGCCTCAG GAAACTTACA ATCACGGGGT AAGGCACCCC  100
TTCTCACAAG GCAGCAGGAG AGAGAATGAG TGCCAGCAGG GGAAATGCCA  150
GATGCTTATA AAACCCTCAG ATCTTGTGAG CACTCACTA
```

Genomic chr7 :

```
gcattcctaa aattagttgc ctcctcactg gctggcagag gggggcctgg  8110836
gtgtaagacc ctgtcttagt ctgttgtcac atttctataa ggaaataccc  8110886
AAGACTGGGT AATTTATAAA GAAATGAGGT TTAATTGACT CACAGTTCCT  8110936
CATGGCTGGG GAGGCCTCAG GAAACTTACA ATCACGGGGT AAGGCACCCC  8110986
TTCTCACAAG GCAGCAGGAG AGAGAATGAG TGCCAGCAGG GGAAATGCCA  8111036
GATGCTTATA AAACCCTCAG ATCTTGTGAG CACTCACTAt cacgagaaca  8111086
gcatgggggg aactgcccca tgattcaatt agctcttact aggtccctcc  8111136
caccacatgt ggggattatg gggattagaa ctcaagatg
```

Side by Side Alignment

```
0000001 aagactgggtaatttataaagaaatgaggtttaattgactcacagttcct 0000050
>>>>>>> |||||||||||||||||||||||||||||||||||||||||||||||||| >>>>>>>
8110887 aagactgggtaatttataaagaaatgaggtttaattgactcacagttcct 8110936

0000051 catggctggggaggcctcaggaaacttacaatcacggggtaaggcacccc 0000100
>>>>>>> |||||||||||||||||||||||||||||||||||||||||||||||||| >>>>>>>
8110937 catggctggggaggcctcaggaaacttacaatcacggggtaaggcacccc 8110986

0000101 ttctcacaaggcagcaggagagagaatgagtgccagcaggggaaatgcca 0000150
>>>>>>> |||||||||||||||||||||||||||||||||||||||||||||||||| >>>>>>>
8110987 ttctcacaaggcagcaggagagagaatgagtgccagcaggggaaatgcca 8111036

0000151 gatgcttataaaaccctcagatcttgtgagcactcacta 0000189
>>>>>>> ||||||||||||||||||||||||||||||||||||||| >>>>>>>
8111037 gatgcttataaaaccctcagatcttgtgagcactcacta 8111075
```

Alignment of YourSeq and chr7:20814366-20814385

Clone 18 YourSeq

```
agtagtgcag aatcacagcc actgagtaat acagtgcccg caaataatga  50
caaccatccc aaacttttat tgtatttcct gagTCTCATT TAACGACCTC  100
AATtccacgg gagtctggtg gcgtttaaag tgtgggggca ttttacggga  150
aggttacatt actgtaaaca tcattaggtc tgtgtcagga agg
```

Genomic chr7 :

```
ataacataaa aataaaaact tggttttaat atattgttat aaaaattcag  20814315
aatgaggcca aagtaaatag gattacaaga cctgtagtgt aaatgtcttt  20814365
TCTCATTTAA CGACCTCAAT atcctgtact ccatgtgtgt tactgcagtc  20814415
ttagattgtc aactcttaag acaatctaca ttatttcttt gtatgattcc  20814465
atgtacctat aagaacttgg
```

Side by Side Alignment

```
00000084 tctcatttaacgacctcaat 00000103
>>>>>>>> |||||||||||||||||||| >>>>>>>>
20814366 tctcatttaacgacctcaat 20814385
```

Alignment of YourSeq and chr17:2706902-2707021

Clone 19 YourSeq

```
ACTTAACCTA CCCAAGGAGA AAGAGTGATT GATTTCATCA TGGTGCTAAG  50
TGACCTTTGC TTCCCTCTCT GTATCTGGAG TTTCTTTGTC TCTTTAAGCG  100
TCTGAAATGA GTGCAGCAAG
```

Genomic chr17 :

```
ttaagagcct gcaatgcagg cagacattgg tggccttcct agcccctggc  2706851
ttcatcccgt tcttctgtcg ctccaaccat cctggtggct ttggccatgg  2706901
ACTTAACCTA CCCAAGGAGA AAGAGTGATT GATTTCATCA TGGTGCTAAG  2706951
TGACCTTTGC TTCCCTCTCT GTATCTGGAG TTTCTTTGTC TCTTTAAGCG  2707001
TCTGAAATGA GTGCAGCAAG cactattgac ccttgctctg caggtgctgg  2707051
tggccacggg aggcctggag caggctgcag ctgccagcct ctcttaagca  2707101
tcccccatac tccatgctgg
```

Side by Side Alignment

```
0000001 acttaacctacccaaggagaaagagtgattgatttcatcatggtgctaag 0000050
>>>>>>> |||||||||||||||||||||||||||||||||||||||||||||||||| >>>>>>>
2706902 acttaacctacccaaggagaaagagtgattgatttcatcatggtgctaag 2706951

0000051 tgacctttgcttccctctctgtatctggagtttctttgtctctttaagcg 0000100
>>>>>>> |||||||||||||||||||||||||||||||||||||||||||||||||| >>>>>>>
2706952 tgacctttgcttccctctctgtatctggagtttctttgtctctttaagcg 2707001

0000101 tctgaaatgagtgcagcaag 0000120
>>>>>>> |||||||||||||||||||| >>>>>>>
2707002 tctgaaatgagtgcagcaag 2707021
```

Alignment of YourSeq and chr2:47043372-47043407

Clone 20 YourSeq

```
aggctaaggg cctttggaag ccgttgagcg aacggggcgt tgcgatcaac  50
accctcctaa ctgcagaact GCCTTTTCTG GTGGCCAAAg GGCCCAAGGC  100
taagcagctc ttcctggagc cactgactga ccaaccgatc accgatgcgc  150
taattcgccg gctccaagca ttgggcgcgc agatcgggct tgggattcgg  200
gtctgcacgc tgaccgaggc attcg
```

Genomic chr2 (reverse strand):

```
ccttgcactg ttcaacctat caggtaggct caagtgttcc actcccatca  47043458
gggggtgggc tgcccagaag cagcagccca gaggggactc ttcacaaatg  47043408
GCCTTTTCTG GTGGCCAAAa GGCCaagagt CAAGGCagag cttcccatgg  47043358
gacagaacta gagacagcca tatggccgag acaccatggc caggcaggaa  47043308
gtctgccata ctgccccaat gagccatggc aggagc
```

Side by Side Alignment

```
00000071 gccttttctggtggccaaagggcc......caaggc 00000100
<<<<<<<< |||||||||||||||||| ||||      |||||| <<<<<<<<
47043407 gccttttctggtggccaaaaggccaagagtcaaggc 47043372
```

Alignment of YourSeq and chr1:208814512-208814586

Clone 21 YourSeq

```
cgaatgcctc ggtcagcgtg cagacccgaa tcccaagccc gatctgcgcg  50
cccaatgctt ggagccggcg aattagcgca tcggtgaTCG GTTGGTCAGT  100
CAGtggctcc aggaagagct gcttagcctt gggccctttg gccaccAGAA  150
AAGGCAGTTC Tgcagttagg agggtgttga tcgcaacgcc ccgttcgctc  200
aacggcttcc aaaggccctt agcctgaatt cagatctccc gggtcaccgc  250
gggcggatcc cccgggctgc ag
```

Genomic chr1 (reverse strand):

```
catggagagg gcctcagagg cagggcccat ggcccatggc cctgataggt  208814637
gtatactcta ggttttcaaa gcaacccagt actgcacaca caaagtctgg  208814587
TCGGTTGGTC AGTCAGcaaa aacaaaaatc aacagatgac aagggtaata  208814537
taaaggaaaA GAAAAGGCAa GTTCTttgtt gtcaggaagc atttagtgta  208814487
acaagggtgg gcaagtccct accttcaggg ggtcccactt gcagtggatt  208814437
cccattgtgt tatggcctaa attaa
```

Side by Side Alignment

```
000000088 tcggttggtcagtcag 000000103
<<<<<<<<< |||||||||||||||| <<<<<<<<<
208814586 tcggttggtcagtcag 208814571
```

```
000000147 agaaaaggca.gttct 000000161
<<<<<<<<< |||||||||| ||||| <<<<<<<<<
208814527 agaaaaggcaagttct 208814512
```

Alignment of YourSeq and chrX:23802458-23802689

Clone 22 YourSeq

```
AGTAATTTGC AGTGAAGGGA AGATTATAAT AGGAAAAACA AACAAAAAGA  50
CCCAGACAGA CAGTGTATCC CTTTGTTCAA AAAGCGTTAA TAACATGATA  100
ATTTATTCAG TAAAAACAAG TATATTTATT CTGTTAACTT TCTAATCAGC  150
AGATCCTGTG AATAGTTTCA TTCAGTTTCT TTTTACCTTT TTAGGAAACG  200
TACTCCCAAA CTAAGCCCAA GATGCAAAGT TT
```

Genomic chrX (reverse strand):

```
gtctattttt tccattttaa tggaagagtg aaatggctgg attgttgaaa  23802740
ccactctgtt tggaatctgc ttcctgtggc agtagtaagc cattctgctg  23802690
AGTAATTTGC AGTGAAGGGA AGATTATAAT AGGAAAAACA AACAAAAAGA  23802640
CCCAGACAGA CAGTGTATCC CTTTGTTCAA AAAGCGTTAA TAACATGATA  23802590
ATTTATTCAG TAAAAACAAG TATATTTATT CTGTTAACTT TCTAATCAGC  23802540
AGATCCTGTG AATAGTTTCA TTCAGTTTCT TTTTACCTTT TTAGGAAACG  23802490
TACTCCCAAA CTAAGCCCAA GATGCAAAGT TTggttcaat gggggttagg  23802440
taagttgatt ttaaattagg cataatcagc tacttcatta acattgaact  23802390
ctttaatcgg agttccatga atgagctcca ag
```

Side by Side Alignment

```
00000001 agtaatttgcagtgaagggaagattataataggaaaaacaaacaaaaaga 00000050
<<<<<<<< |||||||||||||||||||||||||||||||||||||||||||||||||| <<<<<<<<
23802689 agtaatttgcagtgaagggaagattataataggaaaaacaaacaaaaaga 23802640

00000051 cccagacagacagtgtatccctttgttcaaaaagcgttaataacatgata 00000100
<<<<<<<< |||||||||||||||||||||||||||||||||||||||||||||||||| <<<<<<<<
23802639 cccagacagacagtgtatccctttgttcaaaaagcgttaataacatgata 23802590

00000101 atttattcagtaaaaacaagtatatttattctgttaactttctaatcagc 00000150
<<<<<<<< |||||||||||||||||||||||||||||||||||||||||||||||||| <<<<<<<<
23802589 atttattcagtaaaaacaagtatatttattctgttaactttctaatcagc 23802540

00000151 agatcctgtgaatagtttcattcagtttctttttacctttttaggaaacg 00000200
<<<<<<<< |||||||||||||||||||||||||||||||||||||||||||||||||| <<<<<<<<
23802539 agatcctgtgaatagtttcattcagtttctttttacctttttaggaaacg 23802490

00000201 tactcccaaactaagcccaagatgcaaagttt 00000232
<<<<<<<< |||||||||||||||||||||||||||||||| <<<<<<<<
23802489 tactcccaaactaagcccaagatgcaaagttt 23802458
```

Alignment of YourSeq and chr7:29823294-29823513

Clone 23 YourSeq

```
AATACATATC TATATACATA AAACCACATT AAATAAAcCC CAaTCATACT  50
ACAAATTaCA accAAAaTAC AcTAAAATAA GTCATATTAT AAACTGaGGc  100
AAATTACAcT AAAATAAAAA TTATAGTAAA ATGcCTGTTT TATCTTCTCT  150
ATAActTcTT CagggAATAA AAATCAATAA CCCGATAGAA AATACACAAA  200
TAGaCCTAAA CAaTTCACAC
```

Genomic chr7 (reverse strand):

```
aaattataat catactaaaa atatgagaga atttttaaaa tctacaagtc  29823564
agaaagacct ttctaaaatg tatagaagaa aaggtttctg caaagcaata  29823514
AATACATATC TATATACATA AAACCACATT AAATAAAgCC CAgTCATACT  29823464
ACAAATTgCA gggAAAtTAC AtTAAAATAA GTCATATTAT AAACTGgGGg  29823414
AAATTACAgT AAAATAAAAA TTATAGTAAA ATGgCTGTTT TATCTTCTCT  29823364
ATAAggTgTT CctacAATAA AAATCAATAA CCCGATAGAA AATACACAAA  29823314
TAGgCCTAAA CAgTTCACAC ataaggaaac atatatggca attaaacaca  29823264
ttcaaagacc ctcaatgtta ttcctaatga gaaaatgcaa gtcaaaacca  29823214
caatcagata ccattttcac
```

Side by Side Alignment

```
00000001 aatacatatctatatacataaaaccacattaaataaaccccaatcatact 00000050
<<<<<<<< ||||||||||||||||||||||||||||||||||||| |||| ||||||| <<<<<<<<
29823513 aatacatatctatatacataaaaccacattaaataaagcccagtcatact 29823464

00000051 acaaattacaaccaaaatacactaaaataagtcatattataaactgaggc 00000100
<<<<<<<< ||||||| ||   ||| |||| |||||||||||||||||||||||| ||  <<<<<<<<
29823463 acaaattgcagggaaattacattaaaataagtcatattataaactggggg 29823414

00000101 aaattacactaaaataaaaattatagtaaaatgcctgttttatcttctct 00000150
<<<<<<<< |||||||| |||||||||||||||||||||||| |||||||||||||||| <<<<<<<<
29823413 aaattacagtaaaataaaaattatagtaaaatggctgttttatcttctct 29823364

00000151 ataacttcttcagggaataaaaatcaataacccgatagaaaatacacaaa 00000200
<<<<<<<< ||||  | |||    ||||||||||||||||||||||||||||||||||| <<<<<<<<
29823363 ataaggtgttcctacaataaaaatcaataacccgatagaaaatacacaaa 29823314

00000201 tagacctaaacaattcacac 00000220
<<<<<<<< ||| |||||||| ||||||| <<<<<<<<
29823313 taggcctaaacagttcacac 29823294
```

Alignment of YourSeq and chr21:40443401-40443559

Clone 24 YourSeq

```
cggtgacccg ggagatctga attccgatgg acctcgggga gcgccgcaac  50
tcggggggca ATGGAGCGAT GGAactcggg gagcgatgga actcggggag  100
cgatggaact cggggagcga tggaactcgg ggagcgatgg aactcgggga  150
gcgatggaac tcggggagcg atggaactcg gggagcgatg gagcccgggg  200
agcgatggaa ctcggggagc GATGGAGCGA TGGAaactcg gggagcgatg  250
gaactcgggg agcaATGGAG CGATGGAact cggggagcga tggaactcgg  300
ggagccaat
```

Genomic chr21 (reverse strand):

```
gatggagtga tggatgttgt ggtgacagtg gtgttggtgt tgggattgtg  40443610
atggagtgat ggatggtggt ggtgacagtg gtgttggtgt tgggatagtg  40443560
ATGGAGCGAT GGAtgttgtg acagtggtgt tggtgttggg atagtgaaga  40443510
agtaatggat ggtggtggtg atagtgatgg tggtgttggg atagtGATGG  40443460
AGCGATGGAt ggtggtggtg atagtgacgg tggtgttggg atagtgATGG  40443410
AGCGATGGAt ggtggtggtg acagtggtgt tggtgttggg atagtgatgg  40443360
agtgatggat gttgtggtga cagtggtgtt ggtgttggga tagtgatgga  40443310
gtgatggat
```

Side by Side Alignment

```
00000061 atggagcgatgga 00000073
<<<<<<<< ||||||||||||| <<<<<<<<
40443559 atggagcgatgga 40443547
```

```
00000221 gatggagcgatgga 00000234
<<<<<<<< |||||||||||||| <<<<<<<<
40443464 gatggagcgatgga 40443451
```

```
00000265 atggagcgatgga 00000277
<<<<<<<< ||||||||||||| <<<<<<<<
40443413 atggagcgatgga 40443401
```

Alignment of YourSeq and chr5:53384849-53385115

Clone 25 YourSeq

```
CACTCTAGAG CCACTCTTTT TTATTTTTAT TTTTTTTCTT TTTTCATACT  50
CTACTGTTTG GAAACAGGTC ATACTTAGAG AGCCATACTT AGAGAGTAGT  100
TATGCTTTAC TTTCTTTTGG ATAGAATACC TACACAAATT ATTTGGAATT  150
ATTTGGAATT TTTCTACATA GTAGGTTTAT CTCTTCTACC CCATTTATTT  200
GTTGATTTAT TCAATTATTT ACTTATGTCA GTAGAGACTC ATGGATATTT  250
ATTTTATACT TTGTTTT
```

Genomic chr5 (reverse strand):

```
atcatatcaa gggtaaacac tgtcaatgtg acttatcact gttgatataa  53385166
acttttatca cctgcttgag gtagtgtttg tcaggtttct ccacttttc  53385116
CACTCTAGAG CCACTCTTTT TTATTTTTAT TTTTTTTCTT TTTTCATACT  53385066
CTACTGTTTG GAAACAGGTC ATACTTAGAG AGCCATACTT AGAGAGTAGT  53385016
TATGCTTTAC TTTCTTTTGG ATAGAATACC TACACAAATT ATTTGGAATT  53384966
ATTTGGAATT TTTCTACATA GTAGGTTTAT CTCTTCTACC CCATTTATTT  53384916
GTTGATTTAT TCAATTATTT ACTTATGTCA GTAGAGACTC ATGGATATTT  53384866
ATTTTATACT TTGTTTTttg tttgttttgt tgttgtttgt tgttgttgtt  53384816
gtttttggag atgcagtttt gccatattgc tcctagtctc cagggatcct  53384766
cccgggcccc agggatc
```

Side by Side Alignment

```
00000001 cactctagagccactcttttttatttttatttttttcttttttcatact 00000050
<<<<<<<< |||||||||||||||||||||||||||||||||||||||||||||||||| <<<<<<<<
53385115 cactctagagccactcttttttatttttatttttttcttttttcatact 53385066

00000051 ctactgtttggaaacaggtcatacttagagagccatacttagagagtagt 00000100
<<<<<<<< |||||||||||||||||||||||||||||||||||||||||||||||||| <<<<<<<<
53385065 ctactgtttggaaacaggtcatacttagagagccatacttagagagtagt 53385016

00000101 tatgctttactttcttttggatagaatacctacacaaattatttggaatt 00000150
<<<<<<<< |||||||||||||||||||||||||||||||||||||||||||||||||| <<<<<<<<
53385015 tatgctttactttcttttggatagaatacctacacaaattatttggaatt 53384966

00000151 atttggaatttttctacatagtaggtttatctcttctaccccatttattt 00000200
<<<<<<<< |||||||||||||||||||||||||||||||||||||||||||||||||| <<<<<<<<
53384965 atttggaatttttctacatagtaggtttatctcttctaccccatttattt 53384916

00000201 gttgatttattcaattatttacttatgtcagtagagactcatggatattt 00000250
<<<<<<<< |||||||||||||||||||||||||||||||||||||||||||||||||| <<<<<<<<
53384915 gttgatttattcaattatttacttatgtcagtagagactcatggatattt 53384866

00000251 attttatactttgtttt 00000267
<<<<<<<< ||||||||||||||||| <<<<<<<<
53384865 attttatactttgtttt 53384849
```

Alignment of YourSeq and chr17:38529244-38529641

Clone 26 YourSeq

```
GAAGAAAAGC CAAAATGCCt gtataatata acaggGATTC CTGCCCCAGg  50
GCAGAgCCAA TCAAGACAGA GTCCtTGTCT TTggCCGGAC CACAGGATca  100
GTGTTGAAAA tGgGAGGAGg GGtAGAGGtA GAGcGGATGG AGAACAcGGA  150
ATCATTTTCT ATATTTgTAA AGTTaTTCAG gTAAGAAAAT CAGCAATTAC  200
cAgAGCCTAA TgTTAtTAGA CATGTCTTTT CTTCCgTAGT ATGaAAGGTC  250
AATTCTGTTC ATTgaCcTtG GcGATAATCA TAGtAATCgg AAAagAATAC  300
AaTCcTGgGa cGAgTTACCA GAgGGGACAC Taccgatctt gTGCATAGCA  350
TaAATGACAc cagcgAacTt TTtAAataGA AGAGCAcAcA AATCCATTTg  400
tctcggatcc aaagaactgt
```

Genomic chr17 :

```
gcctcccaaa gtgctgggat tacaggcata agccaccgcc ctcggcctca  38529193
tccatgattt tattttgcca tttcaagtga tggagcttgt tttagagctg  38529243
GAAGAAAAGC CAAAATGCCa gttaatctaa actaGATTCC TGCCCCAGtG  38529293
CAGAaCCAAT CAAGACAGAG TCCcTGTCTT TcCCGGACCA CAGGATttGT  38529343
GTTGAAAAgG aGAGGAGtGG gAGAGGcAGA GtGGATGGAG AACAaGGAAT  38529393
CATTTTCTAT ATTTtTAAAG TTcTTCAGtT AAGAAAATCA GCAATTACaA  38529443
tAGCCTAATc TTAcTAGACA TGTCTTTTCT TCCcTAGTAT GtAAGGTCAA  38529493
TTCTGTTCAT TtgCaTaGGa GATAATCATA GgAATCccAA AttAATACAc  38529543
TCtTGtGctG AcTTACCAGA tGGGACACTc taagattttc TGCATAGCAT  38529593
tAATGACAtt ttgtActTcT TcAAcgcGAA GAGCAgAtAA ATCCATTTct  38529643
ttctgttcca atgaacttta acacattaga aaaacatata tatatatctt  38529693
tttaaaaggt ttataaaatg acaacttcat tttatcattt taaaataa
```

Side by Side Alignment

```
00000001 gaagaaaagccaaaatgcc 00000019
>>>>>>>> |||||||||||||||||| >>>>>>>>
38529244 gaagaaaagccaaaatgcc 38529262
```

```
00000036 gattcctgccccagggcagagccaatcaagacagagtccttgtcttt 00000082
>>>>>>>> |||||||||||||| ||||| |||||||||||||||||| ||||||| >>>>>>>>
38529278 gattcctgccccagtgcagaaccaatcaagacagagtccctgtcttt 38529324
```

```
00000085 ccggaccacaggatcagtgttgaaaatgggaggaggggtagaggtagagc 00000134
>>>>>>>> ||||||||||||||  |||||||||| | |||||| || ||||| ||||  >>>>>>>>
38529326 ccggaccacaggatttgtgttgaaaaggagaggagtgggagaggcagagt 38529375

00000135 ggatggagaacacggaatcattttctatatttgtaaagttattcaggtaa 00000184
>>>>>>>> |||||||||||| |||||||||||||||||| ||||||| ||||| ||| >>>>>>>>
38529376 ggatggagaacaaggaatcattttctatatttttaaagttcttcagttaa 38529425

00000185 gaaaatcagcaattaccagagcctaatgttattagacatgtcttttcttc 00000234
>>>>>>>> ||||||||||||||| | |||||||| ||| ||||||||||||||||| >>>>>>>>
38529426 gaaaatcagcaattacaatagcctaatcttactagacatgtcttttcttc 38529475

00000235 cgtagtatgaaaggtcaattctgttcattgaccttggcgataatcatagt 00000284
>>>>>>>> | ||||||| ||||||||||||||||||  | | || |||||||||||  >>>>>>>>
38529476 cctagtatgtaaggtcaattctgttcatttgcataggagataatcatagg 38529525

00000285 aatcggaaaagaatacaatcctgggacgagttaccagaggggacact 00000331
>>>>>>>> ||||  |||  |||||| || || |  || |||||||| |||||||| >>>>>>>>
38529526 aatcccaaattaatacactcttgtgctgacttaccagatgggacact 38529572
```

```
00000342 tgcatagcataaatgacaccagcgaactttttaaatagaagagcacacaa 00000391
>>>>>>>> |||||||||| |||||||      |  | || ||   |||||||| | || >>>>>>>>
```

```
38529584 tgcatagcattaatgacattttgtacttcttcaacgcgaagagcagataa 38529633

00000392 atccattt 00000399
>>>>>>>> |||||||| >>>>>>>>
38529634 atccattt 38529641
```

Alignment of YourSeq and chr4:57025056-57025303

Clone 27 YourSeq

```
TCACAATACC CTATTGTTAA TTCCATATTA AGACTATATA CGGTGTTTTG  50
AAATTGTCTG CCTCTGGAGA CTTCCAAGAT TCATTGAGAG CAGAAGGTTA  100
ATTTTGTTCA TCTTTGCATT CCCAGGGTCT TATATATACA TAATAGGCTG  150
TATTTGTTCT CTAAAAAATA CAGAACCAAT AGGATATGTA CAGATTATGA  200
GAAATTGGCT CTGCAAAATG GAGGTGAAGT CCCACAGTCT GCCTTCTG
```

Genomic chr4 (reverse strand):

```
aggtaagaga gattgagtat agactgtgga gaattagtct ttttcattca  57025354
cagtatacaa ttgttttcta gagatcagag tccttcctac ttttgtgtat  57025304
TCACAATACC CTATTGTTAA TTCCATATTA AGACTATATA CGGTGTTTTG  57025254
AAATTGTCTG CCTCTGGAGA CTTCCAAGAT TCATTGAGAG CAGAAGGTTA  57025204
ATTTTGTTCA TCTTTGCATT CCCAGGGTCT TATATATACA TAATAGGCTG  57025154
TATTTGTTCT CTAAAAAATA CAGAACCAAT AGGATATGTA CAGATTATGA  57025104
GAAATTGGCT CTGCAAAATG GAGGTGAAGT CCCACAGTCT GCCTTCTGca  57025054
ggctgaaggc ccaggaaagc cagtgtagtt ccaaacccga gggcttgaga  57025004
atcaggggag gcaataacgt taagtcctag tctgaaggcc tgaggacc
```

Side by Side Alignment

```
00000001 tcacaataccctattgttaattccatattaagactatatacggtgttttg 00000050
<<<<<<<< |||||||||||||||||||||||||||||||||||||||||||||||||| <<<<<<<<
57025303 tcacaataccctattgttaattccatattaagactatatacggtgttttg 57025254

00000051 aaattgtctgcctctggagacttccaagattcattgagagcagaaggtta 00000100
<<<<<<<< |||||||||||||||||||||||||||||||||||||||||||||||||| <<<<<<<<
57025253 aaattgtctgcctctggagacttccaagattcattgagagcagaaggtta 57025204

00000101 attttgttcatctttgcattcccagggtcttatatatacataataggctg 00000150
<<<<<<<< |||||||||||||||||||||||||||||||||||||||||||||||||| <<<<<<<<
57025203 attttgttcatctttgcattcccagggtcttatatatacataataggctg 57025154

00000151 tatttgttctctaaaaaatacagaaccaataggatatgtacagattatga 00000200
<<<<<<<< |||||||||||||||||||||||||||||||||||||||||||||||||| <<<<<<<<
57025153 tatttgttctctaaaaaatacagaaccaataggatatgtacagattatga 57025104

00000201 gaaattggctctgcaaaatggaggtgaagtcccacagtctgccttctg 00000248
<<<<<<<< |||||||||||||||||||||||||||||||||||||||||||||||| <<<<<<<<
57025103 gaaattggctctgcaaaatggaggtgaagtcccacagtctgccttctg 57025056
```

Alignment of YourSeq and chr16:24411988-24412236

Clone 28 YourSeq

```
cggccgctct agcccgcggt gacccgggag atctgaattC CTTGGCATCC  50
gtcgcaatga acTGAAGGTG GAGGAGATcc cacgggatga catcatacgg  100
gcagcggcat atttcagaga cagtccggaa aaagcacgaa agcgatttgg  150
tgaagaggga tggtacgtca ataaggtgta tgacgcgccg caggccgtaa  200
tggcgctcct gtgccacgga aaaaacctcg gctgggacaa atcgctaagg  250
gaggtgctgc acgtgtttta gaattcagat ctcccgggtc accg
```

Genomic chr16 :

```
cgggcttgag ccaccatgcc cagctattta tttatttttg tggagacagg  24411937
gtctcaccat tttgcctagg ctcatcttaa acggctcaag caatctccca  24411987
CCTTGGCATC Ccaaagtgct gagattacag gcatgtccca ccacgccggc  24412037
ctctctttac ctttctaatg tggctagcag aaagtttaaa attacatgtg  24412087
tgggtcagat gacatttctc ttgggcagca cttccctgga caggcagtgt  24412137
atcccagaaa ggcagacctg ggctagccac agaatcttca accccatccc  24412187
caagacttga ggctgacaag aagtaaaaca aagTGAAGGT GGAGGAGATg  24412237
aaaaatggga tgcaaagaaa cagatgtgat atagtccatc tttctctctc  24412287
ttttttaaaa tatagactgg gtctcactgt gttgcccagg ctggtctca
```

Side by Side Alignment

```
00000040 ccttggcatcc 00000050
>>>>>>>> ||||||||||| >>>>>>>>
24411988 ccttggcatcc 24411998
```

```
00000063 tgaaggtggaggagat 00000078
>>>>>>>> |||||||||||||||| >>>>>>>>
24412221 tgaaggtggaggagat 24412236
```

Alignment of YourSeq and chr1:39189091-39189451

Clone 29 YourSeq

```
ACATGTGCTG TGATTTTATA AGGCAGCGTT ATGATATACA GCATTTTCTA  50
TAGTTACTCA AACAAGAACC TTGCTTTCAA AGATTAGCTA ATGAGACTAG  100
AATTTGTGGA ATATACTTGG ACTACTTGAA CTAAGAGAAA TGACCCATGG  150
AGTAAAATCT TCCCAGTGCA GAATAATTCC CTAGTACCCC ATCAGTCTTA  200
TCACTCAGTG ATAATAATCT CTGCTCCCCA CTTAGAACAA ACAGAATAAA  250
ACATTGTATC AAATTGAGGC TGGTGTGAGT GAAGTGGTGT TCACAACTAA  300
TTGATCACAA CCAGTTATAG ATTTACTTGT CCTTTTCCAC TCCCACCGCT  350
TCACTTCACT A
```

Genomic chr1 (reverse strand):

```
aaaagaagtc agacaagttt gggaagtgct aggtgaagca acattgaaca  39189502
gacttgtttg ttgaaggact tcttagaagg ttagagactt catgacgctg  39189452
ACATGTGCTG TGATTTTATA AGGCAGCGTT ATGATATACA GCATTTTCTA  39189402
TAGTTACTCA AACAAGAACC TTGCTTTCAA AGATTAGCTA ATGAGACTAG  39189352
AATTTGTGGA ATATACTTGG ACTACTTGAA CTAAGAGAAA TGACCCATGG  39189302
AGTAAAATCT TCCCAGTGCA GAATAATTCC CTAGTACCCC ATCAGTCTTA  39189252
TCACTCAGTG ATAATAATCT CTGCTCCCCA CTTAGAACAA ACAGAATAAA  39189202
ACATTGTATC AAATTGAGGC TGGTGTGAGT GAAGTGGTGT TCACAACTAA  39189152
TTGATCACAA CCAGTTATAG ATTTACTTGT CCTTTTCCAC TCCCACCGCT  39189102
TCACTTCACT Agccttacac aaatttgaaa aatcaagttg taaatcaaag  39189052
gcaggtataa acataaaatt gtgaaaactt gtaatggctt gcaaccattt  39189002
gaggaggtgt t
```

Side by Side Alignment

```
00000001 acatgtgctgtgattttataaggcagcgttatgatatacagcattttcta 00000050
<<<<<<<< |||||||||||||||||||||||||||||||||||||||||||||||||| <<<<<<<<
39189451 acatgtgctgtgattttataaggcagcgttatgatatacagcattttcta 39189402

00000051 tagttactcaaacaagaaccttgctttcaaagattagctaatgagactag 00000100
<<<<<<<< |||||||||||||||||||||||||||||||||||||||||||||||||| <<<<<<<<
39189401 tagttactcaaacaagaaccttgctttcaaagattagctaatgagactag 39189352

00000101 aatttgtggaatatacttggactacttgaactaagagaaatgacccatgg 00000150
<<<<<<<< |||||||||||||||||||||||||||||||||||||||||||||||||| <<<<<<<<
39189351 aatttgtggaatatacttggactacttgaactaagagaaatgacccatgg 39189302

00000151 agtaaaatcttcccagtgcagaataattccctagtaccccatcagtctta 00000200
<<<<<<<< |||||||||||||||||||||||||||||||||||||||||||||||||| <<<<<<<<
39189301 agtaaaatcttcccagtgcagaataattccctagtaccccatcagtctta 39189252

00000201 tcactcagtgataataatctctgctccccacttagaacaaacagaataaa 00000250
<<<<<<<< |||||||||||||||||||||||||||||||||||||||||||||||||| <<<<<<<<
39189251 tcactcagtgataataatctctgctccccacttagaacaaacagaataaa 39189202

00000251 acattgtatcaaattgaggctggtgtgagtgaagtggtgttcacaactaa 00000300
<<<<<<<< |||||||||||||||||||||||||||||||||||||||||||||||||| <<<<<<<<
39189201 acattgtatcaaattgaggctggtgtgagtgaagtggtgttcacaactaa 39189152

00000301 ttgatcacaaccagttatagatttacttgtccttttccactcccaccgct 00000350
<<<<<<<< |||||||||||||||||||||||||||||||||||||||||||||||||| <<<<<<<<
39189151 ttgatcacaaccagttatagatttacttgtccttttccactcccaccgct 39189102

00000351 tcacttcacta 00000361
<<<<<<<< ||||||||||| <<<<<<<<
39189101 tcacttcacta 39189091
```

Alignment of YourSeq and chr18:52432512-52432915

Clone 30 YourSeq

```
TTATCCCATT AAtCgTTTTA gTCATTTCAg ACTGAGTGAA TtAATACCAC  50
TTaAAAGAAT CACTTAtACT TACCTAGGAT ACTGAcAATA GcATTGTTAC  100
TCACAAACCT ATTAAATACA TATGAACGAA ATACTACTTA CAGTTACgCT  150
GTTAACATTC TGAAACTTAA CATAACGAAc TGGAACAATG CCATCTTCTT  200
TAATATCATC CTCTGTCAGT TCCAGAGCTc GAGTTGGTTC ACTTCTTTCT  250
GCCTCTTCAA AATCCATAGA TCGGGGTAGG TTGATAAAAA TTTTTACATA  300
TTTAGGGCCC TGACCTATAC AATGGTAGAA TAAAATAAAA TGcTTAAAAA  350
CCTCAAATAT ACTATACTCC CTTTACCACT ACACTCACAC TATACACAGA  400
CACA
```

Genomic chr18 :

```
acttaccata tacatataaa ataactgaca aaaccccaca aatattcaaa  52432461
aatcaatctg tccttcctct ttagggtaca taaattaaaa agaagaaaat  52432511
TTATCCCATT AAaCaTTTTA cTCATTTCAa ACTGAGTGAA TaAATACCAC  52432561
TTtAAAGAAT CACTTAgACT TACCTAGGAT ACTGAaAATA GtATTGTTAC  52432611
TCACAAACCT ATTAAATACA TATGAACGAA ATACTACTTA CAGTTACaCT  52432661
GTTAACATTC TGAAACTTAA CATAACGAAg TGGAACAATG CCATCTTCTT  52432711
TAATATCATC CTCTGTCAGT TCCAGAGCTt GAGTTGGTTC ACTTCTTTCT  52432761
GCCTCTTCAA AATCCATAGA TCGGGGTAGG TTGATAAAAA TTTTTACATA  52432811
TTTAGGGCCC TGACCTATAC AATGGTAGAA TAAAATAAAA TGtTTAAAAA  52432861
CCTCAAATAT ACTATACTCC CTTTACCACT ACACTCACAC TATACACAGA  52432911
CACAcacaaa tcaatacaca tatacttcaa ataactccat tattagaact  52432961
tgtacaaaag tctgagtatt caggtagcca atcctttatt ttccaaaaca  52433011
catt
```

Side by Side Alignment

```
00000001 ttatcccattaatcgttttagtcatttcagactgagtgaattaataccac 00000050
>>>>>>>> |||||||||||| | ||||| |||||||| ||||||||||| |||||||| >>>>>>>>
52432512 ttatcccattaaacattttactcatttcaaactgagtgaataaataccac 52432561

00000051 ttaaaagaatcacttatacttacctaggatactgacaatagcattgttac 00000100
>>>>>>>> || ||||||||||||| ||||||||||||||||| ||||| |||||||| >>>>>>>>
52432562 tttaaagaatcacttagacttacctaggatactgaaaatagtattgttac 52432611

00000101 tcacaaacctattaaatacatatgaacgaaatactacttacagttacgct 00000150
>>>>>>>> ||||||||||||||||||||||||||||||||||||||||||||||| || >>>>>>>>
52432612 tcacaaacctattaaatacatatgaacgaaatactacttacagttacact 52432661

00000151 gttaacattctgaaacttaacataacgaactggaacaatgccatcttctt 00000200
>>>>>>>> |||||||||||||||||||||||||||| ||||||||||||||||||||| >>>>>>>>
52432662 gttaacattctgaaacttaacataacgaagtggaacaatgccatcttctt 52432711

00000201 taatatcatcctctgtcagttccagagctcgagttggttcacttctttct 00000250
>>>>>>>> |||||||||||||||||||||||||||| ||||||||||||||||||||| >>>>>>>>
52432712 taatatcatcctctgtcagttccagagcttgagttggttcacttctttct 52432761

00000251 gcctcttcaaaatccatagatcggggtaggttgataaaaatttttacata 00000300
>>>>>>>> |||||||||||||||||||||||||||||||||||||||||||||||||| >>>>>>>>
52432762 gcctcttcaaaatccatagatcggggtaggttgataaaaatttttacata 52432811

00000301 tttagggccctgacctatacaatggtagaataaaataaaatgcttaaaaa 00000350
>>>>>>>> |||||||||||||||||||||||||||||||||||||||||| ||||||| >>>>>>>>
52432812 tttagggccctgacctatacaatggtagaataaaataaaatgtttaaaaa 52432861

00000351 cctcaaatatactatactccctttaccactacactcacactatacacaga 00000400
>>>>>>>> |||||||||||||||||||||||||||||||||||||||||||||||||| >>>>>>>>
52432862 cctcaaatatactatactccctttaccactacactcacactatacacaga 52432911
```

```
00000401 caca 00000404
>>>>>>>> |||| >>>>>>>>
52432912 caca 52432915
```

Alignment of YourSeq and chr6:30573215-30573572

Clone 31 YourSeq

```
GAGAAAGAAA GAAAGAGAGA GAGAAAGAAA GAAAGGAAAG AAGGAAAGAA  50
GGAAAGAAAG GAAGAAAGGA AGAAAGAAAG AAAGGCAAAT GATCACTTAG  100
AGGATTTTGT TTGGTAGTTA AAACCATTTT GAAACAGAGG GAGGGAAGAA  150
ATCACCTATG CTTCCTCAGT GGTAAAGAGA CTGGGAACCA CCACGCCAGA  200
GTTAGAAAAT ATGAGGCAAC AGAAGGGCTG TTATATGTAG TGAAAATTTC  250
CAAACCCGGT CCCCTGGAGG GAATACTTGG TGACTGGGCC TTAGAGGAAA  300
GAGATGCTTG TCCAGCCCAT TGCCTGTGTG TCCAGGAGAG ACTGTGCCCA  350
CCTTGAGA
```

Genomic chr6 :

```
gatcacacca ttgcactcca gcctgagcaa gaagagtgaa actgcgcttc  30573164
gaaaagaaag aaagagagag agggagggag ggaggaagga aggaaggaga  30573214
GAGAAAGAAA GAAAGAGAGA GAGAAAGAAA GAAAGGAAAG AAGGAAAGAA  30573264
GGAAAGAAAG GAAGAAAGGA AGAAAGAAAG AAAGGCAAAT GATCACTTAG  30573314
AGGATTTTGT TTGGTAGTTA AAACCATTTT GAAACAGAGG GAGGGAAGAA  30573364
ATCACCTATG CTTCCTCAGT GGTAAAGAGA CTGGGAACCA CCACGCCAGA  30573414
GTTAGAAAAT ATGAGGCAAC AGAAGGGCTG TTATATGTAG TGAAAATTTC  30573464
CAAACCCGGT CCCCTGGAGG GAATACTTGG TGACTGGGCC TTAGAGGAAA  30573514
GAGATGCTTG TCCAGCCCAT TGCCTGTGTG TCCAGGAGAG ACTGTGCCCA  30573564
CCTTGAGAga ctgagagaag accctagtga ggagaagccc ccaggccagc  30573614
cgtcagcaca gggcattgga ggtccccaac cagctccaag tcctgaacag  30573664
agcacagc
```

Side by Side Alignment

```
00000001 gagaaagaaagaaagagagagagaaagaaagaaaggaaagaaggaaagaa 00000050
>>>>>>>> |||||||||||||||||||||||||||||||||||||||||||||||||| >>>>>>>>
30573215 gagaaagaaagaaagagagagagaaagaaagaaaggaaagaaggaaagaa 30573264

00000051 ggaaagaaaggaagaaaggaagaaagaaagaaaggcaaatgatcacttag 00000100
>>>>>>>> |||||||||||||||||||||||||||||||||||||||||||||||||| >>>>>>>>
30573265 ggaaagaaaggaagaaaggaagaaagaaagaaaggcaaatgatcacttag 30573314

00000101 aggattttgtttggtagttaaaaccattttgaaacagagggagggaagaa 00000150
>>>>>>>> |||||||||||||||||||||||||||||||||||||||||||||||||| >>>>>>>>
30573315 aggattttgtttggtagttaaaaccattttgaaacagagggagggaagaa 30573364

00000151 atcacctatgcttcctcagtggtaaagagactgggaaccaccacgccaga 00000200
>>>>>>>> |||||||||||||||||||||||||||||||||||||||||||||||||| >>>>>>>>
30573365 atcacctatgcttcctcagtggtaaagagactgggaaccaccacgccaga 30573414

00000201 gttagaaaatatgaggcaacagaagggctgttatatgtagtgaaaatttc 00000250
>>>>>>>> |||||||||||||||||||||||||||||||||||||||||||||||||| >>>>>>>>
30573415 gttagaaaatatgaggcaacagaagggctgttatatgtagtgaaaatttc 30573464

00000251 caaacccggtcccctggagggaatacttggtgactgggccttagaggaaa 00000300
>>>>>>>> |||||||||||||||||||||||||||||||||||||||||||||||||| >>>>>>>>
30573465 caaacccggtcccctggagggaatacttggtgactgggccttagaggaaa 30573514

00000301 gagatgcttgtccagcccattgcctgtgtgtccaggagagactgtgccca 00000350
>>>>>>>> |||||||||||||||||||||||||||||||||||||||||||||||||| >>>>>>>>
30573515 gagatgcttgtccagcccattgcctgtgtgtccaggagagactgtgccca 30573564

00000351 ccttgaga 00000358
>>>>>>>> |||||||| >>>>>>>>
30573565 ccttgaga 30573572
```

Alignment of YourSeq and chr18:52432382-52432890

Clone 32 YourSeq

```
TATAAAGTTG TAGAAcAAAC TTCCAGCAAA ACTTACCATA TACcTATAAA  50
ATAACTGACA AAACCCCACA AATATTCAAA AATCAATCTG TCCTTCCTCT  100
TTAGGGTACA TAgATTAAAA AGAAGAAAAT TTATCCCATT AAACATTTTA  150
CTCATTTCAA ACTGAGTGAA TAAATACCAC TTTAAAGAAT CACTTAGACT  200
TACCTAGGAT ACTGAAAATA GgATTGTTAC TCACAAACCT ATTAAATACA  250
TATGAACGAA ATACTACTTA CAGTTACACT GTTAACATTC TGAAACTTAA  300
CATAACGAAG gGcAAaAATG CCATCTTCTT TAATATCATC CTCTGTCAaT  350
TCaCAGAGCT TGAGTTGGTT CACTTCTTTC TGCCTCTTCA AAATCCATAG  400
ATCGGGGTAG GTTGATAAAt ATTTTTACAT ATTaAGGGCC CTGACCTATA  450
CAATGGTAGA ATAAAATAAA ATGTaTAAAA ACCTCAtATA TACTATACTC  500
CCTTTACCAC
```

Genomic chr18 :

```
acttaaaaac tccatattca agaaaagtaa ataagatttt acaaacacat  52432331
cactactata ccaggtataa agaataaagc acaaatatct tcagttttag  52432381
TATAAAGTTG TAGAAtAAAC TTCCAGCAAA ACTTACCATA TACaTATAAA  52432431
ATAACTGACA AAACCCCACA AATATTCAAA AATCAATCTG TCCTTCCTCT  52432481
TTAGGGTACA TAaATTAAAA AGAAGAAAAT TTATCCCATT AAACATTTTA  52432531
CTCATTTCAA ACTGAGTGAA TAAATACCAC TTTAAAGAAT CACTTAGACT  52432581
TACCTAGGAT ACTGAAAATA GtATTGTTAC TCACAAACCT ATTAAATACA  52432631
TATGAACGAA ATACTACTTA CAGTTACACT GTTAACATTC TGAAACTTAA  52432681
CATAACGAAG tGgAAcAATG CCATCTTCTT TAATATCATC CTCTGTCAgT  52432731
TCCAGAGCTT GAGTTGGTTC ACTTCTTTCT GCCTCTTCAA AATCCATAGA  52432781
TCGGGGTAGG TTGATAAAaA TTTTTACATA TTtAGGGCCC TGACCTATAC  52432831
AATGGTAGAA TAAAATAAAA TGTtTAAAAA CCTCAaATAT ACTATACTCC  52432881
CTTTACCACt acactcacac tatacacaga cacacacaaa tcaatacaca  52432931
tatacttcaa ataactccat tattagaact tgtacaaaag tctgagtatt  52432981
caggtagcc
```

Side by Side Alignment

```
00000001 tataaagttgtagaacaaacttccagcaaaacttaccatatacctataaa 00000050
>>>>>>>> ||||||||||||||| ||||||||||||||||||||||||| |||||| >>>>>>>>
52432382 tataaagttgtagaataaacttccagcaaaacttaccatatacatataaa 52432431

00000051 ataactgacaaaaccccacaaatattcaaaaatcaatctgtccttcctct 00000100
>>>>>>>> |||||||||||||||||||||||||||||||||||||||||||||||||| >>>>>>>>
52432432 ataactgacaaaaccccacaaatattcaaaaatcaatctgtccttcctct 52432481

00000101 ttagggtacatagattaaaaagaagaaaatttatcccattaaacatttta 00000150
>>>>>>>> |||||||||||| ||||||||||||||||||||||||||||||||||||| >>>>>>>>
52432482 ttagggtacataaattaaaaagaagaaaatttatcccattaaacatttta 52432531

00000151 ctcatttcaaactgagtgaataaataccactttaaagaatcacttagact 00000200
>>>>>>>> |||||||||||||||||||||||||||||||||||||||||||||||||| >>>>>>>>
52432532 ctcatttcaaactgagtgaataaataccactttaaagaatcacttagact 52432581

00000201 tacctaggatactgaaaataggattgttactcacaaacctattaaataca 00000250
>>>>>>>> ||||||||||||||||||||| ||||||||||||||||||||||||||||| >>>>>>>>
52432582 tacctaggatactgaaaatagtattgttactcacaaacctattaaataca 52432631

00000251 tatgaacgaaatactacttacagttacactgttaacattctgaaacttaa 00000300
>>>>>>>> |||||||||||||||||||||||||||||||||||||||||||||||||| >>>>>>>>
52432632 tatgaacgaaatactacttacagttacactgttaacattctgaaacttaa 52432681

00000301 cataacgaagggcaaaaatgccatcttctttaatatcatcctctgtcaat 00000350
>>>>>>>> |||||||||| | || ||||||||||||||||||||||||||||||| | >>>>>>>>
52432682 cataacgaagtggaacaatgccatcttctttaatatcatcctctgtcagt 52432731
```

```
00000351 tcacagagcttgagttggttcacttctttctgcctcttcaaaatccatag 00000400
>>>>>>>> || ||||||||||||||||||||||||||||||||||||||||||||||| >>>>>>>>
52432732 tc.cagagcttgagttggttcacttctttctgcctcttcaaaatccatag 52432780

00000401 atcggggtaggttgataaatatttttacatattaagggccctgacctata 00000450
>>>>>>>> ||||||||||||||||||| ||||||||||||| |||||||||||||||| >>>>>>>>
52432781 atcggggtaggttgataaaaatttttacatatttagggccctgacctata 52432830

00000451 caatggtagaataaaataaaatgtataaaaacctcatatatactatactc 00000500
>>>>>>>> |||||||||||||||||||||||| ||||||||||| ||||||||||||| >>>>>>>>
52432831 caatggtagaataaaataaaatgtttaaaaacctcaaatatactatactc 52432880

00000501 cctttaccac 00000510
>>>>>>>> |||||||||| >>>>>>>>
52432881 cctttaccac 52432890
```

Alignment of YourSeq and chr12:112829446-112829770

Clone 33 YourSeq

```
TTGTTCATTT GTTTACTGCC CCTACTGCCA ACCACCATTA GAACATAAGA  50
GCCACGAGGG CAGGAACTTT GCCTTGTTCA CAGCCACCTT CCCAGTGCCG  100
AGCAATGCAC CTGGTACAGA GAGAGGCTCC ATCAACGCCA GAGGAATGCC  150
AGGGGAATAA ATCTGCATCT CCCGTGGTGC AGAGGAGACA GGAGGCACCC  200
CATGCACACT GAAGGAATGA AAGCACGCAT TCATTCCGTC CTTTCAAACC  250
ACAGCgAAGT AGGAAGAGTG GGAATATCCT GATAGGAAAA CAGGGCAGAG  300
TGGGGAAATG ACCCGGTCCT CGCGG
```

Genomic chr12 :

```
atggcagcca cagaaactgg tactggtctt attctcatcc ctcctaactc  112829395
ttgctttaac tgtcttcaga acgctcatgc caccatgtca cggaatttat  112829445
TTGTTCATTT GTTTACTGCC CCTACTGCCA ACCACCATTA GAACATAAGA  112829495
GCCACGAGGG CAGGAACTTT GCCTTGTTCA CAGCCACCTT CCCAGTGCCG  112829545
AGCAATGCAC CTGGTACAGA GAGAGGCTCC ATCAACGCCA GAGGAATGCC  112829595
AGGGGAATAA ATCTGCATCT CCCGTGGTGC AGAGGAGACA GGAGGCACCC  112829645
CATGCACACT GAAGGAATGA AAGCACGCAT TCATTCCGTC CTTTCAAACC  112829695
ACAGCaAAGT AGGAAGAGTG GGAATATCCT GATAGGAAAA CAGGGCAGAG  112829745
TGGGGAAATG ACCCGGTCCT CGCGGccagc cctccgaggg ggtcaacagg  112829795
acttagcttt atttgtaacc cctgtatgtt ttataagggg aggaaaaaaa  112829845
taccttatct gtgtaactga aacta
```

Side by Side Alignment

```
000000001 ttgttcatttgtttactgcccctactgccaaccaccattagaacataaga 000000050
>>>>>>>>> |||||||||||||||||||||||||||||||||||||||||||||||||| >>>>>>>>>
112829446 ttgttcatttgtttactgcccctactgccaaccaccattagaacataaga 112829495

000000051 gccacgagggcaggaactttgccttgttcacagccaccttcccagtgccg 000000100
>>>>>>>>> |||||||||||||||||||||||||||||||||||||||||||||||||| >>>>>>>>>
112829496 gccacgagggcaggaactttgccttgttcacagccaccttcccagtgccg 112829545

000000101 agcaatgcacctggtacagagagaggctccatcaacgccagaggaatgcc 000000150
>>>>>>>>> |||||||||||||||||||||||||||||||||||||||||||||||||| >>>>>>>>>
112829546 agcaatgcacctggtacagagagaggctccatcaacgccagaggaatgcc 112829595

000000151 aggggaataaatctgcatctcccgtggtgcagaggagacaggaggcaccc 000000200
>>>>>>>>> |||||||||||||||||||||||||||||||||||||||||||||||||| >>>>>>>>>
112829596 aggggaataaatctgcatctcccgtggtgcagaggagacaggaggcaccc 112829645

000000201 catgcacactgaaggaatgaaagcacgcattcattccgtcctttcaaacc 000000250
>>>>>>>>> |||||||||||||||||||||||||||||||||||||||||||||||||| >>>>>>>>>
112829646 catgcacactgaaggaatgaaagcacgcattcattccgtcctttcaaacc 112829695

000000251 acagcgaagtaggaagagtgggaatatcctgataggaaaacagggcagag 000000300
>>>>>>>>> ||||| |||||||||||||||||||||||||||||||||||||||||||| >>>>>>>>>
112829696 acagcaaagtaggaagagtgggaatatcctgataggaaaacagggcagag 112829745

000000301 tggggaaatgacccggtcctcgcgg 000000325
>>>>>>>>> ||||||||||||||||||||||||| >>>>>>>>>
112829746 tggggaaatgacccggtcctcgcgg 112829770
```

Alignment of YourSeq and chr3:123217290-123217355

Clone 34 YourSeq

```
GGACTAATAC ACAGTCTACA ATCAGATTGG GCTTATTTAT CGCCGAAATA  50
TTTCCAGAAT CTCTCT
```

Genomic chr3 :

```
tcctgaggcc tccccagccc tgcagaactg taagtgaatt aaacctcttt  123217239
cctttataaa ttatgcaatc tcaggcagtt ctttatagca gcatgagaac  123217289
GGACTAATAC ACAGTCTACA ATCAGATTGG GCTTATTTAT CGCCGAAATA  123217339
TTTCCAGAAT CTCTCTctct ctccaatttc actactatcc tagcacaagc  123217389
tactattatg tccagcctcc atatcacctg tgcttaccta gtactgtact  123217439
catgcatgct ctggtt
```

Side by Side Alignment

```
000000001 ggactaatacacagtctacaatcagattgggcttatttatcgccgaaata 000000050
>>>>>>>>> |||||||||||||||||||||||||||||||||||||||||||||||||| >>>>>>>>>
123217290 ggactaatacacagtctacaatcagattgggcttatttatcgccgaaata 123217339

000000051 tttccagaatctctct 000000066
>>>>>>>>> |||||||||||||||| >>>>>>>>>
123217340 tttccagaatctctct 123217355
```

Alignment of YourSeq and chr4:34957924-34958031

Clone 35 YourSeq

```
tcgcccgccg tgacccggga gatctgaatt cagtcatgtg gggccaaagt  50
ggcctgcacg gcttcagcac ctgtaggtac gaaggatagg tggtgagata  100
tagacaaaga ttatggtgga accattagaa cccgtagcga caggcggcaa  150
agattttatg ggatgtggtg tccagaaaga tctagttagg agaagaagga  200
catcgagtgg gtgatccaga aaatatgatg gttgaggact ttcaccaaat  250
cgccttcgtt attcccgtaa atcaagtccg gaagaggagg tagAAATAAA  300
AAGATAATAG CCCATGG
```

Genomic chr4 :

```
gctctgggcc aacaaagaag aatacaacct taaatctttt ttttatttta  34957873
tttaattatt ttttacaaca gagctctcaa tctactagag tgaagatgcc  34957923
AAATAAAAAG ATAATAGCaa tacagggtat ttagcgttat aatgatagag  34957973
agaggtgcat gtaatatata gaagaggata gcttaattta aactggcttg  34958023
ggCCATGGaa aactcctaag agaagagaat gcatgtatta aatcttaaag  34958073
aattcaaaac tttaggtgaa aaacaaggta aaaaatgagg tattgaatgt  34958123
caagagcc
```

Side by Side Alignment

```
00000294 aaataaaaagataatagc 00000311
>>>>>>>> |||||||||||||||||| >>>>>>>>
34957924 aaataaaaagataatagc 34957941
```

```
00000312 ccatgg 00000317
>>>>>>>> |||||| >>>>>>>>
34958026 ccatgg 34958031
```

Alignment of YourSeq and chr14:30621914-30622069

Clone 36 YourSeq

```
CCATGACTTT AGGTAACTGA GCTGAGTTAC CTTACATCTA AAACAGGAAG  50
AGAGTCTTAC AAGACTAGAT ATTGAGAGAG TTAACGTGAA TCAGAGGTGA  100
AGGGATATGT ATTATAGTTG GATGAGTTGT GTTGATGTGT CGTATATTTA  150
CTAGAT
```

Genomic chr14 :

```
ctgcactcca gcctgggcga cagagcgaga ctccgtctca aaaaaaaaaa  30621863
aaaagactag aagagcttta acacctgatc agccttaata gttgatgttg  30621913
CCATGACTTT AGGTAACTGA GCTGAGTTAC CTTACATCTA AAACAGGAAG  30621963
AGAGTCTTAC AAGACTAGAT ATTGAGAGAG TTAACGTGAA TCAGAGGTGA  30622013
AGGGATATGT ATTATAGTTG GATGAGTTGT GTTGATGTGT CGTATATTTA  30622063
CTAGATgttt agtagaaata aagatgggta tacttgtctt tgtagccttc  30622113
tggtgtgtag tataggtctg tgtgatgagc tccagcattc ttgtttgaat  30622163
taaaat
```

Side by Side Alignment

```
00000001 ccatgactttaggtaactgagctgagttaccttacatctaaaacaggaag 00000050
>>>>>>>> |||||||||||||||||||||||||||||||||||||||||||||||||| >>>>>>>>
30621914 ccatgactttaggtaactgagctgagttaccttacatctaaaacaggaag 30621963

00000051 agagtcttacaagactagatattgagagagttaacgtgaatcagaggtga 00000100
>>>>>>>> |||||||||||||||||||||||||||||||||||||||||||||||||| >>>>>>>>
30621964 agagtcttacaagactagatattgagagagttaacgtgaatcagaggtga 30622013

00000101 agggatatgtattatagttggatgagttgtgttgatgtgtcgtatattta 00000150
>>>>>>>> |||||||||||||||||||||||||||||||||||||||||||||||||| >>>>>>>>
30622014 agggatatgtattatagttggatgagttgtgttgatgtgtcgtatattta 30622063

00000151 ctagat 00000156
>>>>>>>> |||||| >>>>>>>>
30622064 ctagat 30622069
```

Alignment of YourSeq and chr2:142605321-142605568

Clone 37 YourSeq

```
GGgCTGAGCC AGCCAGGCGC CTACtATGCT CCGCAGTTCA CAGCCAGgTG  50
AGAACCCTCA GCCGAGCACA GGGCTGCTTC TGCAGGCAAA ACAAAtCTTC  100
TCCCTTCGTT CCTCGGAAGC TTCCTTCCCG GGAAGGAAGG ACAgGCCAgG  150
GAACAGAGCT TCTCTTAtGG AAATGGTTTC CTCGGCTCTA CAgAGAATCC  200
CAGTACTTCC TGAATTGCAC TgTTAACTTT TATGCTGAGC AGCACCTG
```

Genomic chr2 :

```
gccacaaatc acaattattt taatcccctt gaagggaaag taactcgaag  142605270
ctggttctgt ggggatttgg gtctttggag aagggagaga cttctgcatt  142605320
GGcCTGAGCC AGCCAGGCGC CTACaATGCT CCGCAGTTCA CAGCCAGcTG  142605370
AGAACCCTCA GCCGAGCACA GGGCTGCTTC TGCAGGCAAA ACAAAgCTTC  142605420
TCCCTTCGTT CCTCGGAAGC TTCCTTCCCG GGAAGGAAGG ACAaGCCAaG  142605470
GAACAGAGCT TCTCTTAgGG AAATGGTTTC CTCGGCTCTA CAcAGAATCC  142605520
CAGTACTTCC TGAATTGCAC TtTTAACTTT TATGCTGAGC AGCACCTGgg  142605570
gacaaagagg caaaatttag tgactcagca gaaacagaaa gcaaaacaac  142605620
ccccaaagtc attaaccgtt gcgtaagaag ggaaggttaa tggaaaag
```

Side by Side Alignment

```
000000001 gggctgagccagccaggcgcctactatgctccgcagttcacagccaggtg 000000050
>>>>>>>>> || |||||||||||||||||||| ||||||||||||||||||||| || >>>>>>>>>
142605321 ggcctgagccagccaggcgcctacaatgctccgcagttcacagccagctg 142605370

000000051 agaaccctcagccgagcacagggctgcttctgcaggcaaaacaaatcttc 000000100
>>>>>>>>> ||||||||||||||||||||||||||||||||||||||||||||| |||| >>>>>>>>>
142605371 agaaccctcagccgagcacagggctgcttctgcaggcaaaacaaagcttc 142605420

000000101 tcccttcgttcctcggaagcttccttcccgggaaggaaggacaggccagg 000000150
>>>>>>>>> ||||||||||||||||||||||||||||||||||||||||||| |||| | >>>>>>>>>
142605421 tcccttcgttcctcggaagcttccttcccgggaaggaaggacaagccaag 142605470

000000151 gaacagagcttctcttatggaaatggtttcctcggctctacagagaatcc 000000200
>>>>>>>>> ||||||||||||||||| |||||||||||||||||||||||| ||||||| >>>>>>>>>
142605471 gaacagagcttctcttagggaaatggtttcctcggctctacacagaatcc 142605520

000000201 cagtacttcctgaattgcactgttaacttttatgctgagcagcacctg 000000248
>>>>>>>>> ||||||||||||||||||||| |||||||||||||||||||||||||| >>>>>>>>>
142605521 cagtacttcctgaattgcacttttaacttttatgctgagcagcacctg 142605568
```

Alignment of YourSeq and chr3:111497276-111497704

Clone 38 YourSeq

```
GTTTAATTGG CTCACAGTTC CACATGGCTG GGGAGGTTTC AGGAAACTTA  50
TAATCATGGT GGAAAGGGAA GCAGGCACAT CTTACCAGGC AAGATAGAGA  100
GAGCGTGTGA GTGAAGAAGT AAGATGCCAA ACACTTAAAA AACCATCAGA  150
CCTCATGAGA ACTCACTCAC TATGATGAGA ACAGAATGGG AAAAACCACC  200
CCCgTGATGT AGTCACCTCC CACgAGGTTC CTCCCCCAAC ACCTGAGGAT  250
TACAATTTAA GATGAGATTT GAGTGGGGAC ATCAAGCCTA ACCATATCAT  300
AGGTCCTCTA AAAATAAATT CAGGAATCAA TCAGTCCACA TGGAGAGAAA  350
AGCCTAAGCT TAAAGACAAA ATGAAAGAAC ACGATGTAAT TAAAAGGGAT  400
GCTAGCCTAA ATAAGAAACA ATATCACCG
```

Genomic chr3 :

```
agtgccctac tacctcagta ctaaattatt gtattgtatt agtcgttttc  111497225
acacttctat aaagatctgc ctaggactgg gtactttata aaggaaagag  111497275
GTTTAATTGG CTCACAGTTC CACATGGCTG GGGAGGTTTC AGGAAACTTA  111497325
TAATCATGGT GGAAAGGGAA GCAGGCACAT CTTACCAGGC AAGATAGAGA  111497375
GAGCGTGTGA GTGAAGAAGT AAGATGCCAA ACACTTAAAA AACCATCAGA  111497425
CCTCATGAGA ACTCACTCAC TATGATGAGA ACAGAATGGG AAAAACCACC  111497475
CCCaTGATGT AGTCACCTCC CACcAGGTTC CTCCCCCAAC ACCTGAGGAT  111497525
TACAATTTAA GATGAGATTT GAGTGGGGAC ATCAAGCCTA ACCATATCAT  111497575
AGGTCCTCTA AAAATAAATT CAGGAATCAA TCAGTCCACA TGGAGAGAAA  111497625
AGCCTAAGCT TAAAGACAAA ATGAAAGAAC ACGATGTAAT TAAAAGGGAT  111497675
GCTAGCCTAA ATAAGAAACA ATATCACCGg tggcatctac cttctactta  111497725
agatgtagaa atcggaaaat aatattcttt ccatgtaaca gcatgaataa  111497775
tgtgtataat ctacaaaacc acacatttc
```

Side by Side Alignment

```
000000001 gtttaattggctcacagttccacatggctggggaggtttcaggaaactta 000000050
>>>>>>>>> |||||||||||||||||||||||||||||||||||||||||||||||||| >>>>>>>>>
111497276 gtttaattggctcacagttccacatggctggggaggtttcaggaaactta 111497325

000000051 taatcatggtggaaagggaagcaggcacatcttaccaggcaagatagaga 000000100
>>>>>>>>> |||||||||||||||||||||||||||||||||||||||||||||||||| >>>>>>>>>
111497326 taatcatggtggaaagggaagcaggcacatcttaccaggcaagatagaga 111497375

000000101 gagcgtgtgagtgaagaagtaagatgccaaacacttaaaaaaccatcaga 000000150
>>>>>>>>> |||||||||||||||||||||||||||||||||||||||||||||||||| >>>>>>>>>
111497376 gagcgtgtgagtgaagaagtaagatgccaaacacttaaaaaaccatcaga 111497425

000000151 cctcatgagaactcactcactatgatgagaacagaatgggaaaaaccacc 000000200
>>>>>>>>> |||||||||||||||||||||||||||||||||||||||||||||||||| >>>>>>>>>
111497426 cctcatgagaactcactcactatgatgagaacagaatgggaaaaaccacc 111497475

000000201 cccgtgatgtagtcacctcccacgaggttcctcccccaacacctgaggat 000000250
>>>>>>>>> ||| |||||||||||||||||||| |||||||||||||||||||||||||| >>>>>>>>>
111497476 cccatgatgtagtcacctcccaccaggttcctcccccaacacctgaggat 111497525

000000251 tacaatttaagatgagatttgagtggggacatcaagcctaaccatatcat 000000300
>>>>>>>>> |||||||||||||||||||||||||||||||||||||||||||||||||| >>>>>>>>>
111497526 tacaatttaagatgagatttgagtggggacatcaagcctaaccatatcat 111497575

000000301 aggtcctctaaaaataaattcaggaatcaatcagtccacatggagagaaa 000000350
>>>>>>>>> |||||||||||||||||||||||||||||||||||||||||||||||||| >>>>>>>>>
111497576 aggtcctctaaaaataaattcaggaatcaatcagtccacatggagagaaa 111497625

000000351 agcctaagcttaaagacaaaatgaaagaacacgatgtaattaaaagggat 000000400
>>>>>>>>> |||||||||||||||||||||||||||||||||||||||||||||||||| >>>>>>>>>
111497626 agcctaagcttaaagacaaaatgaaagaacacgatgtaattaaaagggat 111497675
```

```
000000401 gctagcctaaataagaaacaatatcaccg 000000429
>>>>>>>>> ||||||||||||||||||||||||||||| >>>>>>>>>
111497676 gctagcctaaataagaaacaatatcaccg 111497704
```

Alignment of YourSeq and chr7:63778799-63779130

Clone 39 YourSeq

```
CACAACAGTC ATGGAGGCTA GGGAGACAGG TCCCTGAAAG GAGGGCAGGG  50
TGGAGTGAGT GGCCCCCATA TCGATCAAAA AGGGGATGGA CTTACCCTCC  100
ACCTTAAGAG TTACCCGAGG TCCATGATGG TCCATGGGGC CTCCGAGGTG  150
TTCGGGCAGT GTCATTCCTC AGCCGCCAAG CCGAGGAGAT CAGAGAAGGA  200
GTCAGCCAGG GGGCTGTGGG GTTAAGCTCC AGGAGCTCTA GGGATGGCAG  250
CTTGAGTTGG ACAGTCTGAC TTCCAGTGAG GGCCTGCACA AACAGGGCAT  300
GGCTTAGGAG GAACCCCAGG CTGCAGGCAT TC
```

Genomic chr7 (reverse strand):

```
ggacaagtgg ggatgactaa aaggagtgca aaaaggagtg ctgatctagt  63779181
tgacacacaa gtggagtctg gagaggtctg gaggctttgc cgtcaattcc  63779131
CACAACAGTC ATGGAGGCTA GGGAGACAGG TCCCTGAAAG GAGGGCAGGG  63779081
TGGAGTGAGT GGCCCCCATA TCGATCAAAA AGGGGATGGA CTTACCCTCC  63779031
ACCTTAAGAG TTACCCGAGG TCCATGATGG TCCATGGGGC CTCCGAGGTG  63778981
TTCGGGCAGT GTCATTCCTC AGCCGCCAAG CCGAGGAGAT CAGAGAAGGA  63778931
GTCAGCCAGG GGGCTGTGGG GTTAAGCTCC AGGAGCTCTA GGGATGGCAG  63778881
CTTGAGTTGG ACAGTCTGAC TTCCAGTGAG GGCCTGCACA AACAGGGCAT  63778831
GGCTTAGGAG GAACCCCAGG CTGCAGGCAT TCcttggccc agtgactatt  63778781
tttatggcac ttgaagcatg gccctggagg ggctgctggg cgctgcggtc  63778731
tggatgcctt gaaatcccta caggccattg gt
```

Side by Side Alignment

```
00000001 cacaacagtcatggaggctagggagacaggtccctgaaaggagggcaggg 00000050
<<<<<<<< |||||||||||||||||||||||||||||||||||||||||||||||||| <<<<<<<<
63779130 cacaacagtcatggaggctagggagacaggtccctgaaaggagggcaggg 63779081

00000051 tggagtgagtggcccccatatcgatcaaaaaggggatggacttaccctcc 00000100
<<<<<<<< |||||||||||||||||||||||||||||||||||||||||||||||||| <<<<<<<<
63779080 tggagtgagtggcccccatatcgatcaaaaaggggatggacttaccctcc 63779031

00000101 accttaagagttacccgaggtccatgatggtccatggggcctccgaggtg 00000150
<<<<<<<< |||||||||||||||||||||||||||||||||||||||||||||||||| <<<<<<<<
63779030 accttaagagttacccgaggtccatgatggtccatggggcctccgaggtg 63778981

00000151 ttcgggcagtgtcattcctcagccgccaagccgaggagatcagagaagga 00000200
<<<<<<<< |||||||||||||||||||||||||||||||||||||||||||||||||| <<<<<<<<
63778980 ttcgggcagtgtcattcctcagccgccaagccgaggagatcagagaagga 63778931

00000201 gtcagccagggggctgtggggttaagctccaggagctctagggatggcag 00000250
<<<<<<<< |||||||||||||||||||||||||||||||||||||||||||||||||| <<<<<<<<
63778930 gtcagccagggggctgtggggttaagctccaggagctctagggatggcag 63778881

00000251 cttgagttggacagtctgacttccagtgagggcctgcacaaacagggcat 00000300
<<<<<<<< |||||||||||||||||||||||||||||||||||||||||||||||||| <<<<<<<<
63778880 cttgagttggacagtctgacttccagtgagggcctgcacaaacagggcat 63778831

00000301 ggcttaggaggaaccccaggctgcaggcattc 00000332
<<<<<<<< |||||||||||||||||||||||||||||||| <<<<<<<<
63778830 ggcttaggaggaaccccaggctgcaggcattc 63778799
```

Alignment of YourSeq and chr12:60895257-60895391

Clone 40 YourSeq

```
ATTTTGGGTT ATTGGTAAGT CAATTTTTCA CAAATTGTTT ATTAATTGAA  50
TGCAGATGTG CAGTCACCAA AAGCAGCCCT GTGTGTCTTA TCTCTAAATC  100
ATTTGAGTTT TCCCTTCTCA GTAGTTGCTC CAACA
```

Genomic chr12 :

```
gctatctctg tatatcctca taagagacca aaactttcca agacaatcat  60895206
acctccaggt tttggaattg aagaacaaaa gtcaatgtct acttgttcac  60895256
ATTTTGGGTT ATTGGTAAGT CAATTTTTCA CAAATTGTTT ATTAATTGAA  60895306
TGCAGATGTG CAGTCACCAA AAGCAGCCCT GTGTGTCTTA TCTCTAAATC  60895356
ATTTGAGTTT TCCCTTCTCA GTAGTTGCTC CAACAgtagg aataacctga  60895406
aatgggaaaa ggcaaggtaa taggatacaa gaataacaaa ttttccagaa  60895456
catctcatag cctggggaag tactgacatt ttcac
```

Side by Side Alignment

```
00000001 attttgggttattggtaagtcaatttttcacaaattgtttattaattgaa 00000050
>>>>>>>> |||||||||||||||||||||||||||||||||||||||||||||||||| >>>>>>>>
60895257 attttgggttattggtaagtcaatttttcacaaattgtttattaattgaa 60895306

00000051 tgcagatgtgcagtcaccaaaagcagccctgtgtgtcttatctctaaatc 00000100
>>>>>>>> |||||||||||||||||||||||||||||||||||||||||||||||||| >>>>>>>>
60895307 tgcagatgtgcagtcaccaaaagcagccctgtgtgtcttatctctaaatc 60895356

00000101 atttgagttttcccttctcagtagttgctccaaca 00000135
>>>>>>>> ||||||||||||||||||||||||||||||||||| >>>>>>>>
60895357 atttgagttttcccttctcagtagttgctccaaca 60895391
```

Alignment of YourSeq and chr14:102157843-102158116

Clone 41 YourSeq

```
aaatgggaga gggccacaaa ctcgcccatg aaacggttat ggccctgatg  50
ctggcaagcg ggcaacaagc tctctctaga gtgatcacaa gatggagttg  100
ctaacgcggt ggccgccgct gagcgaggcg gtgccccggg cgatagcagt  150
tcgtccgagg gcagaggatc tgaaatgcaa ggggggggatg agcgcgcAAA  200
ACAGTCaTGc TTGGtGAAGG cATATGGGGA ATAaGGGAAA GAGAGGGCCT  250
gcTTgCcCCg TCtccCgaTG AgACAGAgAG ATCTCAGTTG GTCTCaGATa  300
aTGTgaGTAG GGAcTcTGca GGAgGcAccC cAGGTGGGAc GGTCTTCgGA  350
TTGggCCAAT aTAGCgGGCg gaGCTgACCA AggGTTGGAA TCTCTGAcAg  400
TAAAcGATCa TGAAATTCCT CCCCCTCTCC CCGCAAAGAG AAATcCGAAG  450
GCAGACAACa TGGGTAAAgT GTC
```

Genomic chr14 :

```
agggctcttt gtgtgtagac attgctcagt acatagttgg cacgaaataa  102157792
aatcacttaa atgaatgaat gagtgaaata caaaggcgga atttgactgt  102157842
AAAACAGTCt TGTTGGaGAA GGtATATGGG GAATAgGGGA AAGAGAGGGC  102157892
CTctTTtCgC CcTCctaCat TGAtACAGAt AGATCTCAGT TGGTCTCtGA  102157942
TctTGTttGT AGGGAtTtTG ttGGAtGaAt gCaAGGTGGG AtGGTCTTCa  102157992
GATTGtaCCA ATtTAGCtGG CattGCTaAC CAActGTTGG AATCTCTGAa  102158042
AaTAAAaGAT CtTGAAATTC CTCCCCCTCT CCCCGCAAAG AGAAATaCGA  102158092
AGGCAGACAA CTGGGTAAAt TGTCctaccc ctaactgcca tccacagaaa  102158142
agtaacattt tgttttattt gattaactgt gcttgacaac attagaaaca  102158192
gtggaaaatc atctgggact tggg
```

Side by Side Alignment

```
000000198 aaaacagtcatgcttggtgaaggcatatggggaataagggaaagagaggg 000000247
>>>>>>>>> ||||||||| || |||| ||||| |||||||||||| ||||||||||||| >>>>>>>>>
102157843 aaaacagtcttg.ttggagaaggtatatggggaataggggaaagagaggg 102157891

000000248 cctgcttgccccgtctcccgatgagacagagagatctcagttggtctcag 000000297
>>>>>>>>> |||  || | || ||   |  ||| ||||| ||||||||||||||||| | >>>>>>>>>
102157892 cctcttttcgccctcctacattgatacagatagatctcagttggtctctg 102157941

000000298 ataatgtgagtagggactctgcaggaggcaccccaggtgggacggtcttc 000000347
>>>>>>>>> ||  |||  ||||||| | ||  ||| | |  | |||||||| ||||||| >>>>>>>>>
102157942 atcttgtttgtagggattttgttggatgaatgcaaggtgggatggtcttc 102157991

000000348 ggattgggccaatatagcgggcggagctgaccaagggttggaatctctga 000000397
>>>>>>>>>  |||||  ||||| |||| |||   ||| |||||  |||||||||||||| >>>>>>>>>
102157992 agattgtaccaatttagctggcattgctaaccaactgttggaatctctga 102158041

000000398 cagtaaacgatcatgaaattcctccccctctccccgcaaagagaaatccg 000000447
>>>>>>>>>  | |||| |||| |||||||||||||||||||||||||||||||||| || >>>>>>>>>
102158042 aaataaaagatcttgaaattcctccccctctccccgcaaagagaaatacg 102158091

000000448 aaggcagacaacatgggtaaagtgtc 000000473
>>>>>>>>> |||||||||||| |||||||| |||| >>>>>>>>>
102158092 aaggcagacaac.tgggtaaattgtc 102158116
```

Alignment of YourSeq and chr1:167637003-167637200

Clone 42 YourSeq

```
TCCTTTCTCA GATCTCAAAC TCCGTGCTGG GAAAAGCACT ACTCTCTTCA  50
AAGCTGTCAG ACTGGGACGT TTAAGTCTGC AGAAGTTTCT GCTGCCTTTT  100
GTTCAGCTGT GTCTTGCCCC CAGAGGTAGA GTCTACAGAG GCAGGCAGGC  150
CTCCTTGAGC TGTGGTCAGC TCCACCCAGT TGGAGCTTCC CAGCTGCT
```

Genomic chr1 (reverse strand):

```
aggggcaccc ggctgtatga ggtgtcagtt ggcccctact gggaggtgtc  167637251
tcccagttag cctacttggg cgtcagggac ccacttgagg aggcagtctg  167637201
TCCTTTCTCA GATCTCAAAC TCCGTGCTGG GAAAAGCACT ACTCTCTTCA  167637151
AAGCTGTCAG ACTGGGACGT TTAAGTCTGC AGAAGTTTCT GCTGCCTTTT  167637101
GTTCAGCTGT GTCTTGCCCC CAGAGGTAGA GTCTACAGAG GCAGGCAGGC  167637051
CTCCTTGAGC TGTGGTCAGC TCCACCCAGT TGGAGCTTCC CAGCTGCTtt  167637001
gtttacctac ttaagcctca gcaatggcag acatctctcc cctagccttg  167636951
ctgtggcatt gcagtttgat ctcagactgc tgtgttagca gtgagtga
```

Side by Side Alignment

```
000000001 tcctttctcagatctcaaactccgtgctgggaaaagcactactctcttca 000000050
<<<<<<<<< |||||||||||||||||||||||||||||||||||||||||||||||||| <<<<<<<<<
167637200 tcctttctcagatctcaaactccgtgctgggaaaagcactactctcttca 167637151

000000051 aagctgtcagactgggacgtttaagtctgcagaagtttctgctgcctttt 000000100
<<<<<<<<< |||||||||||||||||||||||||||||||||||||||||||||||||| <<<<<<<<<
167637150 aagctgtcagactgggacgtttaagtctgcagaagtttctgctgcctttt 167637101

000000101 gttcagctgtgtcttgcccccagaggtagagtctacagaggcaggcaggc 000000150
<<<<<<<<< |||||||||||||||||||||||||||||||||||||||||||||||||| <<<<<<<<<
167637100 gttcagctgtgtcttgcccccagaggtagagtctacagaggcaggcaggc 167637051

000000151 ctccttgagctgtggtcagctccacccagttggagcttcccagctgct 000000198
<<<<<<<<< |||||||||||||||||||||||||||||||||||||||||||||||| <<<<<<<<<
167637050 ctccttgagctgtggtcagctccacccagttggagcttcccagctgct 167637003
```

Alignment of YourSeq and chrX:36370495-36370707

Clone 43 YourSeq

```
ATTTAGCCAC AGGAAGTTTT CAGGTTCAAC CTACTTTCCT TAGCACTCCA  50
TGCATCACAG ACACTGGTTT TCTATTTAGC AAACCCATTT CCTTTTCTTT  100
TATTTCTTTT TGAGACAGAG TCTCACTCTT ATATGAGGCT GGCATGAGGT  150
GGCACAGTCA CAGATCACTG TAACGTTGAC CACCTGGGCT CAAGTGATTC  200
TCCCACCTCA GCT
```

Genomic chrX :

```
tcaaaataag taaataaata aatgaataaa tggaaccatt acaagctggg  36370444
gtttgtctgt atagccatta atccctaaac catagcctgg tacttgagtt  36370494
ATTTAGCCAC AGGAAGTTTT CAGGTTCAAC CTACTTTCCT TAGCACTCCA  36370544
TGCATCACAG ACACTGGTTT TCTATTTAGC AAACCCATTT CCTTTTCTTT  36370594
TATTTCTTTT TGAGACAGAG TCTCACTCTT ATATGAGGCT GGCATGAGGT  36370644
GGCACAGTCA CAGATCACTG TAACGTTGAC CACCTGGGCT CAAGTGATTC  36370694
TCCCACCTCA GCTtcccaag tagctgctac aacagacaca caccaccatg  36370744
cttggctaat ttgtttatta tttgtagaga caagatcttg ctctgctgcc  36370794
caaaatggtc cca
```

Side by Side Alignment

```
00000001 atttagccacaggaagttttcaggttcaacctactttccttagcactcca 00000050
>>>>>>>> |||||||||||||||||||||||||||||||||||||||||||||||||| >>>>>>>>
36370495 atttagccacaggaagttttcaggttcaacctactttccttagcactcca 36370544

00000051 tgcatcacagacactggttttctatttagcaaacccatttccttttcttt 00000100
>>>>>>>> |||||||||||||||||||||||||||||||||||||||||||||||||| >>>>>>>>
36370545 tgcatcacagacactggttttctatttagcaaacccatttccttttcttt 36370594

00000101 tatttctttttgagacagagtctcactcttatatgaggctggcatgaggt 00000150
>>>>>>>> |||||||||||||||||||||||||||||||||||||||||||||||||| >>>>>>>>
36370595 tatttctttttgagacagagtctcactcttatatgaggctggcatgaggt 36370644

00000151 ggcacagtcacagatcactgtaacgttgaccacctgggctcaagtgattc 00000200
>>>>>>>> |||||||||||||||||||||||||||||||||||||||||||||||||| >>>>>>>>
36370645 ggcacagtcacagatcactgtaacgttgaccacctgggctcaagtgattc 36370694

00000201 tcccacctcagct 00000213
>>>>>>>> ||||||||||||| >>>>>>>>
36370695 tcccacctcagct 36370707
```

Alignment of YourSeq and chr1:121186462-121186687

Clone 44 YourSeq

```
TTGTGATGTG TTcGTTCAAC TCACAGAGTT TAACCTTTCT GTTCATAGAG  50
CAGTTAGGAA ACaCTCTGTT TGTAAAGTCT GTAAGTGGAT ATTCTGACAT  100
tTTGTGGCCT TCGTTGGAAA CGGGATTTCT TCaTATTCTG CTAGACAGAa  150
GAATTCTCAG TAACTTCCTT GTGTTGTGTG TATTCAACTC ACAGAGTTGA  200
ACGATCCTTT ACACAGAGCA GACTTG
```

Genomic chr1 :

```
gtgcaatttg caagtgtaga tttcaagcgc tttaaggtca atggcagaaa  121186411
aggaaatatc ttcgtttcaa aactagacag aatcattccc acaaactgcg  121186461
TTGTGATGTG TTtGTTCAAC TCACAGAGTT TAACCTTTCT GTTCATAGAG  121186511
CAGTTAGGAA ACgCTCTGTT TGTAAAGTCT GTAAGTGGAT ATTCTGACAT  121186561
cTTGTGGCCT TCGTTGGAAA CGGGATTTCT TCcTATTCTG CTAGACAGAt  121186611
GAATTCTCAG TAACTTCCTT GTGTTGTGTG TATTCAACTC ACAGAGTTGA  121186661
ACGATCCTTT ACACAGAGCA GACTTGaaac actctttttg tggaatttgc  121186711
aagtggagat ttcagccgct ttgaggtcaa tggtagaaaa ggaaatatct  121186761
tcgtatagaa acaagacaga atgatt
```

Side by Side Alignment

```
000000001 ttgtgatgtgttcgttcaactcacagagtttaacctttctgttcatagag 000000050
>>>>>>>>> |||||||||||| ||||||||||||||||||||||||||||||||||||| >>>>>>>>>
121186462 ttgtgatgtgtttgttcaactcacagagtttaacctttctgttcatagag 121186511

000000051 cagttaggaaacactctgtttgtaaagtctgtaagtggatattctgacat 000000100
>>>>>>>>> |||||||||||| ||||||||||||||||||||||||||||||||||||| >>>>>>>>>
121186512 cagttaggaaacgctctgtttgtaaagtctgtaagtggatattctgacat 121186561

000000101 tttgtggccttcgttggaaacgggatttcttcatattctgctagacagaa 000000150
>>>>>>>>>  |||||||||||||||||||||||||||||| ||||||||||||||||  >>>>>>>>>
121186562 cttgtggccttcgttggaaacgggatttcttcctattctgctagacagat 121186611

000000151 gaattctcagtaacttccttgtgttgtgtgtattcaactcacagagttga 000000200
>>>>>>>>> |||||||||||||||||||||||||||||||||||||||||||||||||| >>>>>>>>>
121186612 gaattctcagtaacttccttgtgttgtgtgtattcaactcacagagttga 121186661

000000201 acgatcctttacacagagcagacttg 000000226
>>>>>>>>> |||||||||||||||||||||||||| >>>>>>>>>
121186662 acgatcctttacacagagcagacttg 121186687
```

Alignment of YourSeq and chr14:60618889-60619062

Clone 45 YourSeq

```
CTCTGTTTTA GTCAAGTGAA GGGCACACTC CTTTTCATTG GCCACCTCTT  50
ACTTTTCCTC CAAGAGGAGT ACTCACTCCC AGAACCCTTT GCTGGGCTCT  100
CATGCCGAGC TTAGGTGCCC CTTCAGTGTG CTTCTGCAGC ACTCATTACC  150
CCGTAGTAGC ATTGTAGTTT AATT
```

Genomic chr14 :

```
gtctttagtc ctgttgactg ctttgatttt gttttttaac ttgtaccaag  60618838
acctgttgcc atgatgggtc tctgcaaatg ggctttcttg ttccatgagt  60618888
CTCTGTTTTA GTCAAGTGAA GGGCACACTC CTTTTCATTG GCCACCTCTT  60618938
ACTTTTCCTC CAAGAGGAGT ACTCACTCCC AGAACCCTTT GCTGGGCTCT  60618988
CATGCCGAGC TTAGGTGCCC CTTCAGTGTG CTTCTGCAGC ACTCATTACC  60619038
CCGTAGTAGC ATTGTAGTTT AATTcctgtt tgcttgactg aagcagagac  60619088
tcacctgcag acgcctgtag gtgccttcca cgttgctcag atgaacagta  60619138
gagaagggtc aggcctgccc tagg
```

Side by Side Alignment

```
00000001 ctctgttttagtcaagtgaagggcacactccttttcattggccacctctt 00000050
>>>>>>>> |||||||||||||||||||||||||||||||||||||||||||||||||| >>>>>>>>
60618889 ctctgttttagtcaagtgaagggcacactccttttcattggccacctctt 60618938

00000051 acttttcctccaagaggagtactcactcccagaaccctttgctgggctct 00000100
>>>>>>>> |||||||||||||||||||||||||||||||||||||||||||||||||| >>>>>>>>
60618939 acttttcctccaagaggagtactcactcccagaaccctttgctgggctct 60618988

00000101 catgccgagcttaggtgccccttcagtgtgcttctgcagcactcattacc 00000150
>>>>>>>> |||||||||||||||||||||||||||||||||||||||||||||||||| >>>>>>>>
60618989 catgccgagcttaggtgccccttcagtgtgcttctgcagcactcattacc 60619038

00000151 ccgtagtagcattgtagtttaatt 00000174
>>>>>>>> |||||||||||||||||||||||| >>>>>>>>
60619039 ccgtagtagcattgtagtttaatt 60619062
```

Alignment of YourSeq and chr5:2179167-2179189

Clone 46 YourSeq

```
cggtgacccg ggagatctga attccagcgg acaacaacag cgacagcacg  50
atcaagcgca tggggcggCC CATCCTGGGA AAGTGCCgAG CAtaacgcca  100
aggccaaggc gtcggccgat tcggcatacc tgcacgtacg ctacggcgct  150
gcgcgatgct tgaactgtat cgggaaatga ctgcaatcgc gtctgctaac  200
attcatcgtt tcctacaagg agttgggata tgggggcttc gaattcagat  250
ctcccgggtc accg
```

Genomic chr5 (reverse strand):

```
cacagtctcc agccatgtcc catcccacag cctcacactg ggcttggagt  2179240
ccatggtcca tccctgcgag caccttcaca cacgcctcca tgcctttccc  2179190
CCCATCCTGG GAAAGTGCCA GCAgggcagg ccgtcttctt cctcttctga  2179140
gcctgtgcct taagcagcaa agctctgaag atagaaaggt cccccgccca  2179090
gtctccccac ctgtatccat cac
```

Side by Side Alignment

```
0000069 cccatcctgggaaagtgccgagca 0000092
<<<<<<< |||||||||||||||||| |||| <<<<<<<
2179189 cccatcctgggaaagtgcc.agca 2179167
```

Alignment of YourSeq and chr10:132035356-132035475

Clone 47 YourSeq

```
ctatgaacta tctccctctc cacgctcttt gccggaatca ttaacgcagt  50
tggcttcccc ggtttctcag ctacatatcg gtcagtcagg aaaacccccct  100
ttaacgtgag tcaggtcAAC TTTtTaCAAG CAGcAAGCGG GACAATCATA  150
GGGCTTGTTT CgtATTTTga ttgtaagtgg gatagggttt taatttgtct  200
catgccccAT GTaTTGTATA gCATTGTTTC ATACGTTcag ttagccttct  250
tttattgtta gaatgattca agcaggc
```

Genomic chr10 :

```
ctatgatact ctgccctgtg aattctagct gccttggttt ccccaggctc  132035305
tcagctacat ctcctcaatt caggaaaacc accttgccct gagacctgaa  132035355
AACTTTcTcC AAGCAGtAAG CGGGACAATC ATAGGGCTTG TTTCATTTTc  132035405
ttattgtctg tgggttattg ttttttgttg tctgataccc aATGTcTTGT  132035455
ATAcCATTGT TTCATACGTT ttgtctgttt tttttttttt ttagttgatt  132035505
caagcaggtg agtaaagcta ggccctttta ttcattttga gtagaagtag  132035555
aatcttctgt taaagaattt
```

Side by Side Alignment

```
000000118 aacttttacaagcagcaagcgggacaatcatagggcttgtttcgtattt 000000167
>>>>>>>>> |||||| | ||||||| ||||||||||||||||||||||||||  |||| >>>>>>>>>
132035356 aactttctccaagcagtaagcgggacaatcatagggcttgtttc..attt 132035403

000000168 t 000000168
>>>>>>>>> | >>>>>>>>>
132035404 t 132035404
```

```
000000209 atgtattgtatagcattgtttcatacgtt 000000237
>>>>>>>>> |||| ||||||| ||||||||||||||| >>>>>>>>>
132035447 atgtcttgtataccattgtttcatacgtt 132035475
```

Alignment of YourSeq and chr6:50550759-50551092

Clone 48 YourSeq

```
AAACtTACAG tCTGAGCAAT CCTCCAACAA AATAAGCCCC CCAAAAAACC  50
ATACCAAGAC ACATgTTAGT CAAAATTCTC GtAACTAAAG ACAAAAGAAA  100
ATAATCTTGA AAGCAGCAAG AGAAAAAgGA CACTTTAACT TTAGGTGAAA  150
AGCTACTTAA ATtcCAGTGG ATTTCTAATT AGATATCATG AAGTCCAGAG  200
GAAGaGAAAC AATATTcTTA AATcaTGAAA GAAAAcaatc gcgaactata  250
cctttATTTT ATGAgAAGAA TATCTGTCAG GAAcGAAGaT cAATTAAGTC  300
ATAgTCAcAT GAAGGcAAAA AAACACCCTA ATAG
```

Genomic chr6 :

```
acatttatgt catatggagt acaaaagagg gcaagaagac agggggagaa  50550708
gtacttgaag gaataatgac tgtaaacctc caaaatttga cagaagtcat  50550758
AAACcTACAG aCTGAGCAAT CCTCCAACAA AATAAGCCCC CCAAAAAACC  50550808
ATACCAAGAC ACATcTTAGT CAAAATTCTC GaAACTAAAG ACAAAAGAAA  50550858
ATAATCTTGA AAGCAGCAAG AGAAAAAtGA CACTTTAACT TTAGGTGAAA  50550908
AGCTACTTAA ATggCAGTGG ATTTCTAATT AGATATCATG AAGTCCAGAG  50550958
GAAGtGAAAC AATATTtTTA AATgcTGAAA GAAAAgaatt gtgaactaaa  50551008
ATTTTAttat cTGAaAAGAA TATCTGTCAG GAAtGAAGcT aAATTAAGTC  50551058
ATAcTCAgAT GAAGGaAAAA AAACACCCTA ATAGaagttc tctaagcaga  50551108
aagaaaatga taaaatcttg gagcattaga aaggaaataa gaacaatcaa  50551158
aagagtaaaa atgtaataca ttttatttct cctc
```

Side by Side Alignment

```
00000001 aaacttacagtctgagcaatcctccaacaaaataagccccccaaaaaacc 00000050
>>>>>>>> |||| ||||| |||||||||||||||||||||||||||||||||||||||| >>>>>>>>
50550759 aaacctacagactgagcaatcctccaacaaaataagccccccaaaaaacc 50550808

00000051 ataccaagacacatgttagtcaaaattctcgtaactaaagacaaaagaaa 00000100
>>>>>>>> |||||||||||||| |||||||||||||||| ||||||||||||||||||| >>>>>>>>
50550809 ataccaagacacatcttagtcaaaattctcgaaactaaagacaaaagaaa 50550858

00000101 ataatcttgaaagcagcaagagaaaaaggacactttaactttaggtgaaa 00000150
>>>>>>>> ||||||||||||||||||||||||||| |||||||||||||||||||||| >>>>>>>>
50550859 ataatcttgaaagcagcaagagaaaaatgacactttaactttaggtgaaa 50550908

00000151 agctacttaaattccagtggatttctaattagatatcatgaagtccagag 00000200
>>>>>>>> ||||||||||||  |||||||||||||||||||||||||||||||||||||| >>>>>>>>
50550909 agctacttaaatggcagtggatttctaattagatatcatgaagtccagag 50550958

00000201 gaagagaaacaatattcttaaatcatgaaagaaaa 00000235
>>>>>>>> |||| ||||||||||| ||||||  |||||||||| >>>>>>>>
50550959 gaagtgaaacaatatttttaaatgctgaaagaaaa 50550993
```

```
00000256 atttta.....tgagaagaatatctgtcaggaacgaagatcaattaagtc 00000300
>>>>>>>> ||||||     ||| ||||||||||||||||| |||| | ||||||||| >>>>>>>>
50551009 attttattatctgaaaagaatatctgtcaggaatgaagctaaattaagtc 50551058

00000301 atagtcacatgaaggcaaaaaaacaccctaatag 00000334
>>>>>>>> ||| ||| ||||||| ||||||||||||||||| >>>>>>>>
50551059 atactcagatgaaggaaaaaaaacaccctaatag 50551092
```

Alignment of YourSeq and chr2:236765821-236765847

Clone 49 YourSeq

```
ccttccaata tggtcagcat acagtcacgc tggaaactgg agaaattgca  50
cgccaggctt ctggtgctgt actggtgtcg attgacgata ccgttgtatt  100
ggcaaccgtT GTTGCTAAGA AagatGCTAA GCCTGGTcag gatttcttcc  150
ccctgactgt tgattacatg gaaaaaagct atgcggctgg ccgtattcct  200
ggcggttttt tcaagcgcga aggacgtcct tcggagaaag aaacgctgac  250
gtcacgcctg atcgatcgtc cgatccgccc gttatttcct gagggatatc  300
tgaacgaagt acaggtgatc gtgcatgttt tgtcggtgaa tcc
```

Genomic chr2 :

```
tgagaaagag tgggatataa gaggtggttg aggtgtgggc tctgggctcc  236765770
agattggttg gctaacattg gaaaagcatg ctttgtgtgg aattgtttac  236765820
TGTTGCTAAG AAccaGCTAA GCCTGGTggg tggggggcag tcagttcctc  236765870
ctgggtcagc aaggccccag atgtcaaagc atctgaatag agaaagtaag  236765920
ctgggggagg gggctcactc ctgcaat
```

Side by Side Alignment

```
000000110 tgttgctaagaa 000000121
>>>>>>>>> |||||||||||| >>>>>>>>>
236765821 tgttgctaagaa 236765832
```

```
000000126 gctaagcctggt 000000137
>>>>>>>>> |||||||||||| >>>>>>>>>
236765836 gctaagcctggt 236765847
```

Alignment of YourSeq and chr6:78817330-78817484

Clone 50 YourSeq

```
ggaaccaggc agatgttcca ataattgaag ataagcaaga gcactcagtg  50
ggaaagggag taaccccggt ggttagccga atcatgaata gagataacag  100
gacaggatcc tgaGTGGGAA GGGGGAAGTA gggctaaaca atgaaagtgg  150
gataagcaat caaaatgctc ACACTTAGCA ACcaattagc cattccggat  200
ggttgccatg gggaatgtag aaccgagaac gatttcactc ttgtaaccgt  250
tgggaccggg aataggactt gcctgcaatt tttcctcaac aacgggaacg  300
aaagccggag atctaccgga acggaggacg gtttcgcccc cgatcacccg  350
tgagatctga attagaaatt ccccagggga aataaggtaa tgagacatac  400
agtgcagcta gaagaccagc aatccatagg actggtattt aaccgagatg  450
tatagattga tagaattacg gaacaccgtc aattacattc ctccagggat  500
aaagaccggt cgccacagcc tcgtatccga ggtccaaccc acgatctaac  550
agggcggcga acttaatcct ctcgttgcag tgcagacagg gatttggggt  600
gcgaccggcc cgtat
```

Genomic chr6 :

```
tctagcaaca atgcttcaag atacatgaag cattgttttt tattgaagta  78817279
tcattcatac atattaaatt aatctatttt aagaggagag cctgggggct  78817329
GTGGGAAGGG GGAAGTAagt agttataatc aatgggcaga tttcctataa  78817379
tcaataggtg gttataatca agtttcagtt aagcaagatg aataaactct  78817429
agacatttgc tgaataacac tgcattatag tcaacaataa tgtACACTTA  78817479
GCAACatgtt aagaggtaga tctcatgttt gtttttatca caataaaatg  78817529
agattttaaa aagaacatag ttgaatttta ataattatat ataaccgtgt  78817579
accca
```

Side by Side Alignment

```
00000114 gtgggaagggggaagta 00000130
>>>>>>>> ||||||||||||||||| >>>>>>>>
78817330 gtgggaagggggaagta 78817346
```

```
00000171 acacttagcaac 00000182
>>>>>>>> |||||||||||| >>>>>>>>
78817473 acacttagcaac 78817484
```

Alignment of YourSeq and chr6:55345780-55345966

Clone 51 YourSeq

```
CAAACACTAA CACACCCAAA CgCCCACTAT AAACCTACAT ATAGTATCAG  50
GGACAGCAAA ACCTCAAAAA AAAAAAAAAC TTCTATTCAA AATCAGGAAA  100
AATGGGAGAA CAATAACACA CAGTGAGATC CTATACTATA GGCATTGTGC  150
AGCCTCTCTG GCAGTGTGGA TTTCAGTAAG GAGAAC
```

Genomic chr6 :

```
cgcgaactca aagccctgaa tgttaaaaga tgtgcaagct aaatgttgtg  55345729
gcaccttgta atagacagga aacccactgc catcttcccg acacagacac  55345779
CAAACACTAA CACACCCAAA CcCCCACTAT AAACCTACAT ATAGTATCAG  55345829
GGACAGCAAA ACCTCAAAAA AAAAAAAAAa CTTCTATTCA AAATCAGGAA  55345879
AAATGGGAGA ACAATAACAC ACAGTGAGAT CCTATACTAT AGGCATTGTG  55345929
CAGCCTCTCT GGCAGTGTGG ATTTCAGTAA GGAGAACctg aatcagatat  55345979
ctgtgaagaa ccaccttctg tggctcctat tttatcctct gaaaggttct  55346029
tccttgaccg tatttttcca cggccatatc tgaaata
```

Side by Side Alignment

```
00000001 caaacactaacacacccaaacgcccactataaacctacatatagtatcag 00000050
>>>>>>>> |||||||||||||||||||| |||||||||||||||||||||||||||| >>>>>>>>
55345780 caaacactaacacacccaaacccccactataaacctacatatagtatcag 55345829

00000051 ggacagcaaaacctcaaaaaaaaaaaaaa.cttctattcaaaatcaggaa 00000099
>>>>>>>> |||||||||||||||||||||||||||| |||||||||||||||||||| >>>>>>>>
55345830 ggacagcaaaacctcaaaaaaaaaaaaaaacttctattcaaaatcaggaa 55345879

00000100 aaatgggagaacaataacacacagtgagatcctatactataggcattgtg 00000149
>>>>>>>> |||||||||||||||||||||||||||||||||||||||||||||||||| >>>>>>>>
55345880 aaatgggagaacaataacacacagtgagatcctatactataggcattgtg 55345929

00000150 cagcctctctggcagtgtggatttcagtaaggagaac 00000186
>>>>>>>> ||||||||||||||||||||||||||||||||||||| >>>>>>>>
55345930 cagcctctctggcagtgtggatttcagtaaggagaac 55345966
```

Alignment of YourSeq and chr17:40282696-40283217

Clone 52 YourSeq

```
GAGAAAAGAA ATAATGCCCC TTGGCATCAA ACCCTTCACC CGTCCTCTGG  50
CTaTCAGGCG TCCcAGGGGT TCACCTCTAG CTGCTCCCTT GAGAGTCCAG  100
CTTCCCGCTC ATCTTTCTGC CCCAGGTCCC AACAGCTGtT TCAAGCATCT  150
GACCACACAA CCTGGGGAAA CCACTTATCT GACAGTTCAC GAAACACTTT  200
CACATGTATT GTGTATCTGA GAAACCTCAC AGCAGCCTGC AAGGTAGGCA  250
GGCCAGGGGT GGTTATTGTC ATGTTACACA TCAGGAAACT GAGGCTCAGT  300
GAGAAAGATT TGTCCACTGT CACACAGTTA GCAAGTGGCT GTCTGCTTTT  350
TAATCTCAAG AGGAAGAGAA ATTCAACATC TGTTCTGTTG CATGATTTTT  400
aTCACCCgTA GGGCAAACTG TCATGAGACA CTGTCCCCAC AGGCCTGGGA  450
CAGGAGCtTA AACAGGGAAG ATGGCTCAGT ATAGGGGaTT CACCAAGCAT  500
GTGATGTGGA GCCCAGAACA aG
```

Genomic chr17 (reverse strand):

```
tggacagccc cggctcctat aggagtccta cttctctcca gcatcctgtg  40283268
ccatcctctt gacgtaatcg ctgtacattg tgtacacagc acctgtgtga  40283218
GAGAAAAGAA ATAATGCCCC TTGGCATCAA ACCCTTCACC CGTCCTCTGG  40283168
CTcTCAGGCG TCCtAGGGGT TCACCTCTAG CTGCTCCCTT GAGAGTCCAG  40283118
CTTCCCGCTC ATCTTTCTGC CCCAGGTCCC AACAGCTGcT TCAAGCATCT  40283068
GACCACACAA CCTGGGGAAA CCACTTATCT GACAGTTCAC GAAACACTTT  40283018
CACATGTATT GTGTATCTGA GAAACCTCAC AGCAGCCTGC AAGGTAGGCA  40282968
GGCCAGGGGT GGTTATTGTC ATGTTACACA TCAGGAAACT GAGGCTCAGT  40282918
GAGAAAGATT TGTCCACTGT CACACAGTTA GCAAGTGGCT GTCTGCTTTT  40282868
TAATCTCAAG AGGAAGAGAA ATTCAACATC TGTTCTGTTG CATGATTTTT  40282818
cTCACCCcTA GGGCAAACTG TCATGAGACA CTGTCCCCAC AGGCCTGGGA  40282768
CAGGAGCcTA AACAGGGAAG ATGGCTCAGT ATAGGGGcTT CACCAAGCAT  40282718
GTGATGTGGA GCCCAGAACA gGagaactta attcacagtc cttttttttt  40282668
tttttttttt tttttgagac agagtctcac tctgtcaccc gggctggagt  40282618
gcagtggcgg atctcagccc ac
```

Side by Side Alignment

```
00000001 gagaaaagaaataatgccccttggcatcaaacccttcacccgtcctctgg 00000050
<<<<<<<< |||||||||||||||||||||||||||||||||||||||||||||||||| <<<<<<<<
40283217 gagaaaagaaataatgccccttggcatcaaacccttcacccgtcctctgg 40283168

00000051 ctatcaggcgtcccaggggttcacctctagctgctcccttgagagtccag 00000100
<<<<<<<< || |||||||||| |||||||||||||||||||||||||||||||||||| <<<<<<<<
40283167 ctctcaggcgtcctaggggttcacctctagctgctcccttgagagtccag 40283118

00000101 cttcccgctcatctttctgccccaggtcccaacagctgtttcaagcatct 00000150
<<<<<<<< ||||||||||||||||||||||||||||||||||||||| ||||||||||| <<<<<<<<
40283117 cttcccgctcatctttctgccccaggtcccaacagctgcttcaagcatct 40283068

00000151 gaccacacaacctggggaaaccacttatctgacagttcacgaaacacttt 00000200
<<<<<<<< |||||||||||||||||||||||||||||||||||||||||||||||||| <<<<<<<<
40283067 gaccacacaacctggggaaaccacttatctgacagttcacgaaacacttt 40283018

00000201 cacatgtattgtgtatctgagaaacctcacagcagcctgcaaggtaggca 00000250
<<<<<<<< |||||||||||||||||||||||||||||||||||||||||||||||||| <<<<<<<<
40283017 cacatgtattgtgtatctgagaaacctcacagcagcctgcaaggtaggca 40282968

00000251 ggccaggggtggttattgtcatgttacacatcaggaaactgaggctcagt 00000300
<<<<<<<< |||||||||||||||||||||||||||||||||||||||||||||||||| <<<<<<<<
40282967 ggccaggggtggttattgtcatgttacacatcaggaaactgaggctcagt 40282918

00000301 gagaaagatttgtccactgtcacacagttagcaagtggctgtctgctttt 00000350
<<<<<<<< |||||||||||||||||||||||||||||||||||||||||||||||||| <<<<<<<<
40282917 gagaaagatttgtccactgtcacacagttagcaagtggctgtctgctttt 40282868
```

```
00000351 taatctcaagaggaagagaaattcaacatctgttctgttgcatgattttt 00000400
<<<<<<<< |||||||||||||||||||||||||||||||||||||||||||||||||| <<<<<<<<
40282867 taatctcaagaggaagagaaattcaacatctgttctgttgcatgattttt 40282818

00000401 atcacccgtagggcaaactgtcatgagacactgtccccacaggcctggga 00000450
<<<<<<<<  |||||| |||||||||||||||||||||||||||||||||||||||||| <<<<<<<<
40282817 ctcacccctagggcaaactgtcatgagacactgtccccacaggcctggga 40282768

00000451 caggagcttaaacagggaagatggctcagtataggggattcaccaagcat 00000500
<<<<<<<< ||||||| ||||||||||||||||||||||||||||| |||||||||||| <<<<<<<<
40282767 caggagcctaaacagggaagatggctcagtataggggcttcaccaagcat 40282718

00000501 gtgatgtggagcccagaacaag 00000522
<<<<<<<< |||||||||||||||||||| | <<<<<<<<
40282717 gtgatgtggagcccagaacagg 40282696
```

Alignment of YourSeq and chr15:53425196-53425231

Clone 53 YourSeq

```
cancagggcc ggatctcgct cgccaagacc ggtgatgtcg atcaccccgc  50
agacccggcc gtcgagatcg tgcagtggct cgctcaggca gaacaccggc  100
gcgaaccgct gcaggtagtg ttcgttgccg cgcaccagtg ccggcacgtc  150
GTCAgCCAGG CTGaccgccg gcgcgGTGGT GCCGATCTCG Gtctcgccca  200
gctgtcgccc gacct
```

Genomic chr15 (reverse strand):

```
ctgggaaaga gagactttga actactaacc ataatgaggg tactgggcac  53425282
agatgaaggt atcaagaaga attttttttt tttagacaga atctcgctct  53425232
GTCAcCCAGG CTGgagtgca GTGGTGCCGA TCTCGGctca atgcaacctc  53425182
cacctccagg gttcaagtga ttctcctgtc tcagcctccc aagtagctgg  53425132
cattacaggt gcgtgccacc acacccagct aatttt
```

Side by Side Alignment

```
00000151 gtcagccaggctg 00000163
<<<<<<<< |||| |||||||| <<<<<<<<
53425231 gtcacccaggctg 53425219
```

```
00000176 gtggtgccgatctcgg 00000191
<<<<<<<< |||||||||||||||| <<<<<<<<
53425211 gtggtgccgatctcgg 53425196
```

Alignment of YourSeq and chr5:2179167-2179189

Clone 54 YourSeq

```
gaagccccca tatcccaact ccttgtagga aacgatgaat gttagcagac  50
gcgattgcag tcatttcccg atacagttca agcatcgcgc agcgccgtag  100
cgtacgtgca ggtatgccga atcggccgac gccttggcct tggcgttaTG  150
CTcGGCACTT TCCCAGGATG GGccgcccca tgcgcttgat cgtgctgtcg  200
ctgttgttgt ccgctg
```

Genomic chr5 :

```
gtgatggata caggtgggga gactgggcgg gggacctttc tatcttcaga  2179116
gctttgctgc ttaaggcaca ggctcagaag aggaagaaga cggcctgccc  2179166
TGCTGGCACT TTCCCAGGAT GGGgggaaag gcatggaggc gtgtgtgaag  2179216
gtgctcgcag ggatggacca tggactccaa gcccagtgtg aggctgtggg  2179266
atgggacatg gctggagact gtg
```

Side by Side Alignment

```
0000149 tgctcggcactttcccaggatggg 0000172
>>>>>>> |||| ||||||||||||||||||| >>>>>>>
2179167 tgct.ggcactttcccaggatggg 2179189
```

Alignment of YourSeq and chr12:121363540-121363770

Clone 55 YourSeq

```
CCACCAGTCA TCAGGGGCAG GGGCTTTTTC AACCTACTCC CCATACCTCC  50
TGTGCCAGCT TCCATGGAAC GCTACCAGCG TGTCTCCCAC TGTTCCTCCC  100
TCCCGTTAAC AGTCCCAGCC ATATTGTGCC AAGTCACTGA CAGGAAGATG  150
TGATTCCCTC AGGTCTGTTG GGGCAGAAAA ATAGTAGAAA ATCCAAAGCA  200
GCCCAGGCTC TTTCTGCCCT GGGATCCGGT C
```

Genomic chr12 :

```
aagactaagg aatagcaaag tgactgcttt gttttgagaa gcactgttcc  121363489
cattacacag tcctcttacc gcagaaggcc ctggaaatct gaccacaaag  121363539
CCACCAGTCA TCAGGGGCAG GGGCTTTTTC AACCTACTCC CCATACCTCC  121363589
TGTGCCAGCT TCCATGGAAC GCTACCAGCG TGTCTCCCAC TGTTCCTCCC  121363639
TCCCGTTAAC AGTCCCAGCC ATATTGTGCC AAGTCACTGA CAGGAAGATG  121363689
TGATTCCCTC AGGTCTGTTG GGGCAGAAAA ATAGTAGAAA ATCCAAAGCA  121363739
GCCCAGGCTC TTTCTGCCCT GGGATCCGGT Ccctccccac ctgaatgtgt  121363789
tcagggatca gaggtctggg gagtacagca ccaggaacag ctcagcacag  121363839
gctggggtta gcgtgacagc agctggagcc a
```

Side by Side Alignment

```
000000001 ccaccagtcatcaggggcaggggctttttcaacctactccccatacctcc 000000050
>>>>>>>>> |||||||||||||||||||||||||||||||||||||||||||||||||| >>>>>>>>>
121363540 ccaccagtcatcaggggcaggggctttttcaacctactccccatacctcc 121363589

000000051 tgtgccagcttccatggaacgctaccagcgtgtctcccactgttcctccc 000000100
>>>>>>>>> |||||||||||||||||||||||||||||||||||||||||||||||||| >>>>>>>>>
121363590 tgtgccagcttccatggaacgctaccagcgtgtctcccactgttcctccc 121363639

000000101 tcccgttaacagtcccagccatattgtgccaagtcactgacaggaagatg 000000150
>>>>>>>>> |||||||||||||||||||||||||||||||||||||||||||||||||| >>>>>>>>>
121363640 tcccgttaacagtcccagccatattgtgccaagtcactgacaggaagatg 121363689

000000151 tgattccctcaggtctgttggggcagaaaaatagtagaaaatccaaagca 000000200
>>>>>>>>> |||||||||||||||||||||||||||||||||||||||||||||||||| >>>>>>>>>
121363690 tgattccctcaggtctgttggggcagaaaaatagtagaaaatccaaagca 121363739

000000201 gcccaggctctttctgccctgggatccggtc 000000231
>>>>>>>>> ||||||||||||||||||||||||||||||| >>>>>>>>>
121363740 gcccaggctctttctgccctgggatccggtc 121363770
```

Alignment of YourSeq and chr12:49427254-49427530

Clone 56 YourSeq

```
GCCAAATGTG ACTAGTGGCT ACCATATTAG ACAGTGCAGA TCTATACTCA  50
TTCCTTCAAA TACATTACTC TGTTAATCTC AAAGTGTGGC CAACTCCCAT  100
TTACTTAGAG GCTGATTATC TAGTTTTGTA GTTCCTCAGG CCTCTAATTT  150
ATTTTTTGCT TTaTGCTTTA TTGATCTTTT GCATGTCCCC CAAAGCCAGA  200
ATCTGCTCAT GGAGGAAGGA AGGCTAAGAA CTGTCCTTGA GGAACTTCAT  250
CAGTAACTCT GGTAAAGCAT CCTGTGG
```

Genomic chr12 :

```
ttaatgagtt attttacttt ttttcatatt aggcctttga aattcagtgt  49427203
gtattaaaca catctcaatt caaaccatca ccatatttca agtctttgta  49427253
GCCAAATGTG ACTAGTGGCT ACCATATTAG ACAGTGCAGA TCTATACTCA  49427303
TTCCTTCAAA TACATTACTC TGTTAATCTC AAAGTGTGGC CAACTCCCAT  49427353
TTACTTAGAG GCTGATTATC TAGTTTTGTA GTTCCTCAGG CCTCTAATTT  49427403
ATTTTTTGCT TTcTGCTTTA TTGATCTTTT GCATGTCCCC CAAAGCCAGA  49427453
ATCTGCTCAT GGAGGAAGGA AGGCTAAGAA CTGTCCTTGA GGAACTTCAT  49427503
CAGTAACTCT GGTAAAGCAT CCTGTGGagg gagaggagag cccagtcatt  49427553
tgcttagatg gtgtttgtgg gatcacctgc agatttaaag cctactctgc  49427603
cacagactcg agtaagaaaa tgttctc
```

Side by Side Alignment

```
00000001 gccaaatgtgactagtggctaccatattagacagtgcagatctatactca 00000050
>>>>>>>> |||||||||||||||||||||||||||||||||||||||||||||||||| >>>>>>>>
49427254 gccaaatgtgactagtggctaccatattagacagtgcagatctatactca 49427303

00000051 ttccttcaaatacattactctgttaatctcaaagtgtggccaactcccat 00000100
>>>>>>>> |||||||||||||||||||||||||||||||||||||||||||||||||| >>>>>>>>
49427304 ttccttcaaatacattactctgttaatctcaaagtgtggccaactcccat 49427353

00000101 ttacttagaggctgattatctagttttgtagttcctcaggcctctaattt 00000150
>>>>>>>> |||||||||||||||||||||||||||||||||||||||||||||||||| >>>>>>>>
49427354 ttacttagaggctgattatctagttttgtagttcctcaggcctctaattt 49427403

00000151 attttttgctttatgctttattgatcttttgcatgtcccccaaagccaga 00000200
>>>>>>>> |||||||||||| ||||||||||||||||||||||||||||||||||||| >>>>>>>>
49427404 attttttgctttctgctttattgatcttttgcatgtcccccaaagccaga 49427453

00000201 atctgctcatggaggaaggaaggctaagaactgtccttgaggaacttcat 00000250
>>>>>>>> |||||||||||||||||||||||||||||||||||||||||||||||||| >>>>>>>>
49427454 atctgctcatggaggaaggaaggctaagaactgtccttgaggaacttcat 49427503

00000251 cagtaactctggtaaagcatcctgtgg 00000277
>>>>>>>> ||||||||||||||||||||||||||| >>>>>>>>
49427504 cagtaactctggtaaagcatcctgtgg 49427530
```

Alignment of YourSeq and chr16:44962360-44962766

Clone 57 YourSeq

```
ATTCGATTCT AtTCGAAAAT GATTCCATTT GAATCCATTT GATGATGATT  50
CCATTAGATT CCATTTGATG ATTCCATTCg ATTCCATTtG ACGATGATTC  100
CATTCGATTC CaTTTGATGA TTCCATTCTA TTCTATTCGA TGATGATTCC  150
ATTCGATTCC ATTTGATGAT GATTGGATTC GATTCCATTT GATGATTTCA  200
TTTGATTCCA TTCGTTAATG ATTTCGATCA AcTCCATTCG ATGATTCCAT  250
TtGATTCCAT TCGATAATGA TTACATCCGA GTCCATTCGA TGATTCCATT  300
TGAGCCcGTT CGATAATTCC ATTTGAGTCC AATCAATGAT TCCATTCATT  350
TCCATTCAAT GATTCCATTC GAGTCCATTT GATCATTCtG GTAGGGTCCg  400
TTCGATG
```

Genomic chr16 :

```
ttccatgcga tgattctatt cgattccatt caatgataat tccattcaat  44962309
tcagtcattc ctctggactc catttgatga tgattccatt caatgattcc  44962359
ATTCGATTCT AcTCGAAAAT GATTCCATTT GAATCCATTT GATGATGATT  44962409
CCATTAGATT CCATTTGATG ATTCCATTCa ATTCCATTcG ACGATGATTC  44962459
CATTCGATTC CgTTTGATGA TTCCATTCTA TTCTATTCGA TGATGATTCC  44962509
ATTCGATTCC ATTTGATGAT GATTGGATTC GATTCCATTT GATGATTTCA  44962559
TTTGATTCCA TTCGTTAATG ATTTCGATCA AtTCCATTCG ATGATTCCAT  44962609
TcGATTCCAT TCGATAATGA TTACATCCGA GTCCATTCGA TGATTCCATT  44962659
TGAGCCtGTT CGATAATTCC ATTTGAGTCC AATCAATGAT TCCATTCATT  44962709
TCCATTCAAT GATTCCATTC GAGTCCATTT GATCATTCcG GTAGGGTCCa  44962759
TTCGATGttg attccattgg agtgcattcg atgattccat tcgagtccat  44962809
tttaggattt gattcgagtc ctttcgatga ttccattcga ttccactcga  44962859
tgatgat
```

Side by Side Alignment

```
00000001 attcgattctattcgaaaatgattccatttgaatccatttgatgatgatt 00000050
>>>>>>>> ||||||||||| |||||||||||||||||||||||||||||||||||||| >>>>>>>>
44962360 attcgattctactcgaaaatgattccatttgaatccatttgatgatgatt 44962409

00000051 ccattagattccatttgatgattccattcgattccatttgacgatgattc 00000100
>>>>>>>> |||||||||||||||||||||||||||| |||||||| ||||||||||| >>>>>>>>
44962410 ccattagattccatttgatgattccattcaattccattcgacgatgattc 44962459

00000101 cattcgattccatttgatgattccattctattctattcgatgatgattcc 00000150
>>>>>>>> ||||||||||| |||||||||||||||||||||||||||||||||||||| >>>>>>>>
44962460 cattcgattccgtttgatgattccattctattctattcgatgatgattcc 44962509

00000151 attcgattccatttgatgatgattggattcgattccatttgatgatttca 00000200
>>>>>>>> |||||||||||||||||||||||||||||||||||||||||||||||||| >>>>>>>>
44962510 attcgattccatttgatgatgattggattcgattccatttgatgatttca 44962559

00000201 tttgattccattcgttaatgatttcgatcaactccattcgatgattccat 00000250
>>>>>>>> ||||||||||||||||||||||||||||||| |||||||||||||||||| >>>>>>>>
44962560 tttgattccattcgttaatgatttcgatcaattccattcgatgattccat 44962609

00000251 ttgattccattcgataatgattacatccgagtccattcgatgattccatt 00000300
>>>>>>>> | |||||||||||||||||||||||||||||||||||||||||||||||| >>>>>>>>
44962610 tcgattccattcgataatgattacatccgagtccattcgatgattccatt 44962659

00000301 tgagcccgttcgataattccatttgagtccaatcaatgattccattcatt 00000350
>>>>>>>> |||||| ||||||||||||||||||||||||||||||||||||||||||| >>>>>>>>
44962660 tgagcctgttcgataattccatttgagtccaatcaatgattccattcatt 44962709

00000351 tccattcaatgattccattcgagtccatttgatcattctggtagggtccg 00000400
>>>>>>>> ||||||||||||||||||||||||||||||||||||||| ||||||||||  >>>>>>>>
44962710 tccattcaatgattccattcgagtccatttgatcattccggtagggtcca 44962759
```

```
00000401 ttcgatg 00000407
>>>>>>>> ||||||| >>>>>>>>
44962760 ttcgatg 44962766
```

Alignment of YourSeq and chrX:135058016-135058039

Clone 58 YourSeq

```
aggacgtatg gtcagcgttc taccatcact tgactccact agggtgtgac  50
accgccggta ggtcatcagg gctgaagact tggatttcag ggggttaggt  100
caggccgtgc ggttAGGTCT CCCATCTCCC CCCggcTCCA Ccaaatccaa  150
aaaagaagac gccctagggc gtc
```

Genomic chrX (reverse strand):

```
caaccacccc acaagcatcc cagtcaagaa aaccgaggcg ggccccctca  135058090
cgtctactcc tcgtcgccac ccccgccatc ctcgttgagc gaagcaaggc  135058040
AGGTCTCCCA TCTCCCCCCT CCACgatccc cgccatcctt gatgggaaaa  135057990
ccaagggagt cgacccaccg cttcatcctc acccgctccc cacagcccct  135057940
gctagctatc ctggctgaaa gaac
```

Side by Side Alignment

```
000000115 aggtctcccatctccccccggctccac 000000141
<<<<<<<<< |||||||||||||||||||   ||||| <<<<<<<<<
135058039 aggtctcccatctcccccc...tccac 135058016
```

Alignment of YourSeq and chr20:30059491-30059513

Clone 59 YourSeq

```
ggcagcaCCG GCAGGATGCA GGGGctgAGC AGGgtcaatg cgccccccag  50
gtaagcaagc agcaacagca gcatggtcag gctccggaaa caggagaaga  100
accgaccggc acccgccagc cgtcggcggt gccatccgtg gcgccgggaa  150
cgaacaccat cgccgcaccg ttcatgcagt agcgcagccc ggtcgggcgc  200
gggccgtcgt tgaacacatg gccgaggtgg ccaccgcagc ggcggcagtg  250
cacctctacc cgcagcatcc cgaaggtgac gtcgcg
```

Genomic chr20 :

```
acatccagag cagagtcagg aattggctag cacaccctgg cctcttcctg  30059440
ttcgcctggc aggctgagtg gagatctcac ttcctgagct gggggtgac  30059490
CCGGCAGGAT GCAGGGGAGC AGGagggagt ggctgtgaga gccagcacta  30059540
agcaacctgg gccatttccc ctccaaggga ggggaaggaa gaggaagggc  30059590
tagtggctag gagtctcctg tag
```

Side by Side Alignment

```
00000008 ccggcaggatgcaggggctgagcagg 00000033
>>>>>>>> ||||||||||||||||   |||||| >>>>>>>>
30059491 ccggcaggatgcagggg...agcagg 30059513
```

Alignment of YourSeq and chr11:85215148-85215513

Clone 60 YourSeq

```
TGTGAGCAAA GGTTGAATTT CAGACCAGGA GCCTGGTCTT GAGGCAATGT  50
CCAGAGGTCA AGGAGGCAAG AAATCAAGTC CAGGGGAGTC AGGTGAGAGA  100
GAAGCTAAAA CAGGCTGTGC TGGTCTAGGG AGCCAGCGTC TGGAGGACAA  150
GGGGCTGGGG AGAAACAGAA AGCAAACAAT TCTGCTGATA CTTGCTGAGC  200
CTCTAACGGT ATGTCAGAGA GAGAGCTCTC ACTTTAGGAG AAGTTGCTAC  250
CCATGCTCGT TTAACTCCCT TCAGCCTGGA GGTGGATGTG CTATAAAGCT  300
CTAGTTCACA GTCGGCAGGG ACTCAGCATC ACAACATATT CACTCCTCGT  350
GAAGGCAGGT ATGAAT
```

Genomic chr11 :

```
ctgtctgtga gggtttgggc agatggccag gctgctctgc atcagaggag  85215097
ccaggccctc aatccatctg tgcagagaca gtctgcagat tccatgtgta  85215147
TGTGAGCAAA GGTTGAATTT CAGACCAGGA GCCTGGTCTT GAGGCAATGT  85215197
CCAGAGGTCA AGGAGGCAAG AAATCAAGTC CAGGGGAGTC AGGTGAGAGA  85215247
GAAGCTAAAA CAGGCTGTGC TGGTCTAGGG AGCCAGCGTC TGGAGGACAA  85215297
GGGGCTGGGG AGAAACAGAA AGCAAACAAT TCTGCTGATA CTTGCTGAGC  85215347
CTCTAACGGT ATGTCAGAGA GAGAGCTCTC ACTTTAGGAG AAGTTGCTAC  85215397
CCATGCTCGT TTAACTCCCT TCAGCCTGGA GGTGGATGTG CTATAAAGCT  85215447
CTAGTTCACA GTCGGCAGGG ACTCAGCATC ACAACATATT CACTCCTCGT  85215497
GAAGGCAGGT ATGAATcctg ccgaaaggtc ttctctcctg ctgacatgaa  85215547
gagatgaaca gtgaaagtgg gaaagtgaaa tttcagggac catttctgaa  85215597
agttctgtga aaaaca
```

Side by Side Alignment

```
00000001 tgtgagcaaaggttgaatttcagaccaggagcctggtcttgaggcaatgt 00000050
>>>>>>>> |||||||||||||||||||||||||||||||||||||||||||||||||| >>>>>>>>
85215148 tgtgagcaaaggttgaatttcagaccaggagcctggtcttgaggcaatgt 85215197

00000051 ccagaggtcaaggaggcaagaaatcaagtccaggggagtcaggtgagaga 00000100
>>>>>>>> |||||||||||||||||||||||||||||||||||||||||||||||||| >>>>>>>>
85215198 ccagaggtcaaggaggcaagaaatcaagtccaggggagtcaggtgagaga 85215247

00000101 gaagctaaaacaggctgtgctggtctagggagccagcgtctggaggacaa 00000150
>>>>>>>> |||||||||||||||||||||||||||||||||||||||||||||||||| >>>>>>>>
85215248 gaagctaaaacaggctgtgctggtctagggagccagcgtctggaggacaa 85215297

00000151 ggggctggggagaaacagaaagcaaacaattctgctgatacttgctgagc 00000200
>>>>>>>> |||||||||||||||||||||||||||||||||||||||||||||||||| >>>>>>>>
85215298 ggggctggggagaaacagaaagcaaacaattctgctgatacttgctgagc 85215347

00000201 ctctaacggtatgtcagagagagagctctcactttaggagaagttgctac 00000250
>>>>>>>> |||||||||||||||||||||||||||||||||||||||||||||||||| >>>>>>>>
85215348 ctctaacggtatgtcagagagagagctctcactttaggagaagttgctac 85215397

00000251 ccatgctcgtttaactcccttcagcctggaggtggatgtgctataaagct 00000300
>>>>>>>> |||||||||||||||||||||||||||||||||||||||||||||||||| >>>>>>>>
85215398 ccatgctcgtttaactcccttcagcctggaggtggatgtgctataaagct 85215447

00000301 ctagttcacagtcggcagggactcagcatcacaacatattcactcctcgt 00000350
>>>>>>>> |||||||||||||||||||||||||||||||||||||||||||||||||| >>>>>>>>
85215448 ctagttcacagtcggcagggactcagcatcacaacatattcactcctcgt 85215497

00000351 gaaggcaggtatgaat 00000366
>>>>>>>> |||||||||||||||| >>>>>>>>
85215498 gaaggcaggtatgaat 85215513
```

Alignment of YourSeq and chr6:17281926-17281947

Clone 61 YourSeq

```
tgtcctccca agcggtggtc gtcatggccg accgttcggg ttcgagggct  50
cccaaagctt cgaagtcgtc gtcggcagcc aggatcacct tgcggaacat  100
cgcattgcgc accatgacgg tgaAGGCTCg tTGGTTGGAC GTGAACGtcc  150
gcgccgggcg gacctcctcg ggttcctcgt cgaggggatg ggtgaggtcc  200
tcccattcgt cgatgaggga ggaatcggtg gag
```

Genomic chr6 (reverse strand):

```
tcattagatt tacagcctac cctaaatcca ggataattta atctcgaggt  17281998
tctagcaaat gcatctgcaa agaccctatt tccaaacaag ggcacttctg  17281948
AGGCTCTGGT TGGACGTGAA CGgggggtgg gacatgattc aacccactat  17281898
caggaggacc tgggaagtgg tgctgaatgg gggatggaac atgccatcaa  17281848
gttaaaggtt ttctctagaa ct
```

Side by Side Alignment

```
00000124 aggctcgttggttggacgtgaacg 00000147
<<<<<<<< ||||||  |||||||||||||||| <<<<<<<<
17281947 aggctc..tggttggacgtgaacg 17281926
```

Alignment of YourSeq and chr18:52432453-52432884

Clone 62 YourSeq

```
ATATTgAAAA ATaAATCTGT CCTTCCTCTT TAGGGTACtT AAATTAAAAA  50
GAAcAAAATT TcTCCCATaA AACATTTTAC TCATTTCAgA CTGAGTGAAT  100
AAtTcCCACT TTAAAGAAcC ACTTAGACTT ACCTAGGATA CTGAAAATAG  150
aATTGTTACT CACAAACCTA TcAAATACAT ATaAACGAAA TACTACTTgC  200
AGTTACAtgT GTTAACATTC TGAAACTTAA CATAACGAAG TGGAACAgTG  250
CCATCTTCTT TAATATCATC CTCTGTCAaT TCCAGAGgTT GAGTTGGTTC  300
ACTcTCTTTC TGCCTCTTCA AAATCCATAG ATCGGGGggt GTTGATAAAA  350
ATTTTTACAT ATTTAGGGCC CTGACCTATA CAATGGTAGA ATAAAATAAA  400
ATGTTTAAAA ACCaCAtATA TACTATtCTC CCTT
```

Genomic chr18 :

```
gaataaagca caaatatctt cagttttagt ataaagttgt agaataaact  52432402
tccagcaaaa cttaccatat acatataaaa taactgacaa aaccccacaa  52432452
ATATTcAAAA ATcAATCTGT CCTTCCTCTT TAGGGTACaT AAATTAAAAA  52432502
GAAgAAAATT TaTCCCATtA AACATTTTAC TCATTTCAaA CTGAGTGAAT  52432552
AAaTaCCACT TTAAAGAAtC ACTTAGACTT ACCTAGGATA CTGAAAATAG  52432602
tATTGTTACT CACAAACCTA TtAAATACAT ATgAACGAAA TACTACTTaC  52432652
AGTTACAcTG TTAACATTCT GAAACTTAAC ATAACGAAGT GGAACAaTGC  52432702
CATCTTCTTT AATATCATCC TCTGTCAgTT CCAGAGcTTG AGTTGGTTCA  52432752
CTTCTTTCTG CCTCTTCAAA ATCCATAGAT CGGGGtagGT TGATAAAAAT  52432802
TTTTACATAT TTAGGGCCCT GACCTATACA ATGGTAGAAT AAAATAAAAT  52432852
GTTTAAAAAC CtCAaATATA CTATaCTCCC TTtaccacta cactcacact  52432902
atacacagac acacacaaat caatacacat atacttcaaa taactccatt  52432952
attagaactt gtacaaaagt ctgagtattc ag
```

Side by Side Alignment

```
00000001 atattgaaaaataaatctgtccttcctctttagggtacttaaattaaaaa 00000050
>>>>>>>> ||||| |||||| ||||||||||||||||||||||||| ||||||||||| >>>>>>>>
52432453 atattcaaaaatcaatctgtccttcctctttagggtacataaattaaaaa 52432502

00000051 gaacaaaatttctcccataaaacattttactcatttcagactgagtgaat 00000100
>>>>>>>> ||| ||||||| |||||| ||||||||||||||||||| ||||||||||| >>>>>>>>
52432503 gaagaaaatttatcccattaaacattttactcatttcaaactgagtgaat 52432552

00000101 aattcccactttaaagaaccacttagacttacctaggatactgaaaatag 00000150
>>>>>>>> || | ||||||||||||| ||||||||||||||||||||||||||||||| >>>>>>>>
52432553 aaataccactttaaagaatcacttagacttacctaggatactgaaaatag 52432602

00000151 aattgttactcacaaacctatcaaatacatataaacgaaatactacttgc 00000200
>>>>>>>>  |||||||||||||||||||| |||||||||| ||||||||||||||| | >>>>>>>>
52432603 tattgttactcacaaacctattaaatacatatgaacgaaatactacttac 52432652

00000201 agttaca 00000207
>>>>>>>> ||||||| >>>>>>>>
52432653 agttaca 52432659
```

```
00000210 tgttaacattctgaaacttaacataacgaagtggaacagtgccatcttct 00000259
>>>>>>>> |||||||||||||||||||||||||||||||||||||| ||||||||||| >>>>>>>>
52432661 tgttaacattctgaaacttaacataacgaagtggaacaatgccatcttct 52432710

00000260 ttaatatcatcctctgtcaattccagaggttgagttggttcactctcttt 00000309
>>>>>>>> ||||||||||||||||||| |||||||| ||||||||||||||| ||||| >>>>>>>>
52432711 ttaatatcatcctctgtcagttccagagcttgagttggttcact.tcttt 52432759

00000310 ctgcctcttcaaaatccatagatcgggggggtgttgataaaaattttaca 00000359
>>>>>>>> ||||||||||||||||||||||||||||   ||||||||||||||||||| >>>>>>>>
```

```
52432760 ctgcctcttcaaaatccatagatcggggtaggttgataaaaatttttaca 52432809

00000360 tatttagggccctgacctatacaatggtagaataaaataaaatgtttaaa 00000409
>>>>>>>> |||||||||||||||||||||||||||||||||||||||||||||||||| >>>>>>>>
52432810 tatttagggccctgacctatacaatggtagaataaaataaaatgtttaaa 52432859

00000410 aaccacatatatactattctccctt 00000434
>>>>>>>> |||| || ||||||||| ||||||| >>>>>>>>
52432860 aacctcaaatatactatactccctt 52432884
```

Alignment of YourSeq and chr6:36919097-36919116

Clone 63 YourSeq

```
cggtgacccg ggagatctga attccagctc ggcgtggcgc caagttccac  50
caaTGAGCCA ATGACTGGCA GAAcgcttta gggctaggat tgcctcaggg  100
gcgtagtagg agttggtgtc agtctgcgtc cgaattcaga tctcccgggt  150
caccgcgggc ggatgcgg
```

Genomic chr6 (reverse strand):

```
aaggctttct ctggcttctg gaacctgcat tggcctctct gtgctggtta  36919167
ggccagaagt gccaggaaat taataccctc caaaagcagc cctcagccac  36919117
TGAGCCAATG ACTGGCAGAA gttggtataa acatatccca gcttcctcac  36919067
ccctctggca gcctaagttt gaaatgcgtt ctacagtctc ccagagttct  36919017
gcagcaggac tataccctag
```

Side by Side Alignment

```
00000054 tgagccaatgactggcagaa 00000073
<<<<<<<< |||||||||||||||||||| <<<<<<<<
36919116 tgagccaatgactggcagaa 36919097
```

Alignment of YourSeq and chr12:106065163-106065182

Clone 64 YourSeq

```
cggtgacccg ggagatctga attcgaatcg ctctattcgc cgaccaacat  50
tatgctgatg catcacgtca ccgcggctct gcgcgcccat gcgctgttta  100
cgcgcgacgt agattacatt gtgaaggacg gcgaagtggt tatcgtcgac  150
gagcacaccg gccgtaccat gcagggccgc cgctggtcgg atggtctgca  200
ccaggcgatt gaagccaaag agggtgtgga gatccagaac gagaaccaga  250
cgcTGGCTTC TATCACCTTC CAGaactact tccgtctcta cgataagctg  300
gccggtatga ccggtacggc ggacaccgag gccttcgagt tcagctctat  350
ttgaattcag atctcccggg tcaccg
```

Genomic chr12 (reverse strand):

```
gttcttcatt cctcccttat ctcagagagg tgcacctaca tttagctcac  106065233
tctgaataaa atgtctgcca ggtcagagga gtaaatcatt gcaagctcaa  106065183
TGGCTTCTAT CACCTTCCAG ccatggcagc agctagaaac aaataatatt  106065133
accatagaag gcagtaattc tgcttctcag ccagtcaggt catctaagct  106065083
taaggtccta ggttcaagcc
```

Side by Side Alignment

```
000000254 tggcttctatcaccttccag 000000273
<<<<<<<<< |||||||||||||||||||| <<<<<<<<<
106065182 tggcttctatcaccttccag 106065163
```

Alignment of YourSeq and chr11:121068026-121068045

Clone 65 YourSeq

```
gcggccgctc tagcccgcgg tgacCCGGGA GATCTGAATT CAGCgtagag  50
aactcgcgct gacgaatata gtaatctgtg atgccaatcc cgagcggcgt  100
taaacggtaa atggcgttgc cttcagcctg ctcgctggta aagcggttca  150
gcagacgttg acgcaccatg tcgttgatcg cgttgttggc gcggacgccg  200
atggtttcgc tggtttgctc aaacgcatca ctcacatggc ggaacgcatc  250
gaattcagat ctcccgggtc accg
```

Genomic chr11 (reverse strand):

```
tttggagcca aacagaggaa actaaggaaa ccttgtctgc tggggttggc  121068096
ttccctagag aaaggtgata tttgtggcaa aagaatacta gggtagggag  121068046
CCGGGAGATC TGAATTCAGC tcttggttgg accacagtct ctatgataat  121067996
gtccctgaag cacagtttcc tcatctataa aactaaagag tagaaaagag  121067946
tctctagggt ctcttctctg
```

Side by Side Alignment

```
000000025 ccgggagatctgaattcagc 000000044
<<<<<<<<< |||||||||||||||||||| <<<<<<<<<
121068045 ccgggagatctgaattcagc 121068026
```

Alignment of YourSeq and chr6_random:936738-936766

Clone 66 YourSeq

```
cggtgacccg ggagatctga attccTGCAG CAGGCTTCCT GTCTCACTTC  50
gtagagaact accatgaagg ctggctgcac attgactgct ccgcaacgta  100
ccgtaaagcg gcggttgagc agtggtctgc gggcgcgacc ggtctgggcg  150
tacgtaccgt tgcgaacctg ctgacggctg agtaatcgtc attgccctcg  200
aattcagatc tcccgggtca ccg
```

Genomic chr6_random (reverse strand):

```
aaaattaatt gaggggctgt gattctctgt ttttataatt caaattagtc  936817
ttttgtccat tcgacctaga agttttctcc ttatataaat taagtaggaa  936767
TGCAGCAGGC TTCCTGTCaa ttTCACTTCt aggaacacca ttattaatta  936717
gccaatgcca gagctctaca tgagtcagac tattctgatt gcagctttcc  936667
ctctgctggc cattacagta gtagctgtg
```

Side by Side Alignment

```
000026 tgcagcaggcttcctgtc....tcacttc 000050
<<<<<< ||||||||||||||||||    ||||||| <<<<<<
936766 tgcagcaggcttcctgtcaatttcacttc 936738
```

Alignment of YourSeq and chr7:10534236-10534257

Clone 67 YourSeq

```
cggtGACCcG GGAGATCTGA ATTCTGatcg tgacccgcaa cggccatcac  50
cgcacctgta ccgtattcca tcagcacgaa gtttgccacc cagacaggga  100
tcgcttcgcc tgtcagcggg tgtttggcat agaagcctgt tgcaacgcct  150
tttttctcca tcgttgccat atcggcttcg gccactttgg tgctgcggca  200
ttcgtcgatg aaggaggcca gttccggatt gttctcggct gctttctgcg  250
ccagcgggtg acctgcggcc acggccaggt agggaattca gatctcccgg  300
gtcaccg
```

Genomic chr7 (reverse strand):

```
cactcatctg ggctacctgg attccccata actaccagga ggagaggcca  10534308
agtctgctgg tccacagaga ctgcagccac ccttcccccct aggggctcag  10534258
GACCaGGGAG ATCTGAATTC TGtccctgag cctctggctg tcgttagcct  10534208
cttccctgta gagatcctgc agggaagccc cacccactga tgggtgatgg  10534158
acaagggtta gacctgaaga gg
```

Side by Side Alignment

```
00000005 gacccgggagatctgaattctg 00000026
<<<<<<<< |||| ||||||||||||||||| <<<<<<<<
10534257 gaccagggagatctgaattctg 10534236
```

Alignment of YourSeq and chr10:135350611-135351584

Clone 68 YourSeq

```
cggtgacccg ggagatctga attcCTGATC ACtCAGGaGA TGTAACTCTT  50
GTCTAGGCTC gGCCTACAtG GGCTTTGTGA CATATTTCTG cACTGATCAC  100
CCAGGTGATG TAACTCTTGT CTAGGCtCCA CCTACGGGGG TATTGTGACG  150
TATCTCaGCA AgGATCACCC AGGTGATGTA gaattcagat ctcccgggtc  200
acaga
```

Genomic chr10 (reverse strand):

```
tgcctatggg ggctttgtga catatctctg cactgatcac cttggtgatg  135351635
ggacttttgt ctacgctctg cctattgggg cattgtgaca tatctctaca  135351585
CTGATCACcC AGGtGATGTA Actcttgtgt tggatctgcc tatgggggca  135351535
ttgcgacata tttctgcact gatcacccag gggatgggac tgttgtctag  135351485
gctctgtgta tgggggcctt gtcacatatc tctgcactga tcacccaggc  135351435
gatggaacac ttgtctaggc tctgcctaca tgggcattgt gacacatctc  135351385
tgaactgatc aacaaagtga tgtaactctt gtctaggctc tgcctacagg  135351335
ggctttgtga catatctctg cactgatcac ccaggtgatg ggacttttgt  135351285
caaggatatg gctacaggga cattgtgaca tatctctgca ctgatcaccc  135351235
aggtgatgta acccttgtct aggctctgcc aaaaaggggc attgtgacat  135351185
aactctgcac tgatcaccca ggtgatggga ctcttgtcta ggctctgcct  135351135
acaggggcat tgtgatatat ctctgcagtg atcacgcagg tgatgtaact  135351085
cttgtctata tctgcctact ggcggcattg tggcatattt ctgcactgat  135351035
cacccaggtg atggactctt gtcttggatc tgcctatggg ggcatagtga  135350985
cataactctg caatgatcac tcatgtgatg taacgcttgt ctaagctgtg  135350935
cctaaagggg aattgtgaca catatctcca ctgatcaccc aggtgatgta  135350885
acaattttct gggatttgtc tacagggggc tttgtgacat atctttgcac  135350835
tgatcaccca ggagatgtaa ctcttgtcta gactcgacct acaggggctt  135350785
tgtgacatat ttctgcactg ataacccagg tgatgtaaCT CTTGTCTAGG  135350735
CTCtGCCTAC AgGGGCTTTG TGACATATTT CTGtACTGAT CACCCAGGTG  135350685
ATGTAACTCT TGTCTAGGCc CCACCTACaG GGGGTATTGT GACGTATCTC  135350635
tGCAAtGATC ACCCAGGTGA TGTAacactc atctaggctc tgcctacagg  135350585
ggcgttttga catagctctg cacagatcat ctaggtgatg taactcttgt  135350535
ccactctctg cctacagggg gcat
```

Side by Side Alignment

```
000000025 ctgatcactcaggagatgtaa 000000045
<<<<<<<<< |||||||| |||| ||||||| <<<<<<<<<
135351584 ctgatcacccaggtgatgtaa 135351564
```

```
000000046 ctcttgtctaggctcggcctacatgggctttgtgacatatttctgcactg 000000095
<<<<<<<<< ||||||||||||||| ||||||| ||||||||||||||||||||| |||| <<<<<<<<<
135350746 ctcttgtctaggctctgcctacaggggctttgtgacatatttctgtactg 135350697

000000096 atcacccaggtgatgtaactcttgtctaggctccacctac.gggggtatt 000000144
<<<<<<<<< ||||||||||||||||||||||||||||||| ||||||| ||||||||| <<<<<<<<<
135350696 atcacccaggtgatgtaactcttgtctaggccccacctacagggggtatt 135350647

000000145 gtgacgtatctcagcaaggatcacccaggtgatgta 000000180
<<<<<<<<< |||||||||||| |||| |||||||||||||||||| <<<<<<<<<
135350646 gtgacgtatctctgcaatgatcacccaggtgatgta 135350611
```

Alignment of YourSeq and chr3:162080146-162080517

Clone 69 YourSeq

```
gcggccgctc tagcccgcgg tgacccggga gatctgAATT CCTTTAGGAC  50
ATgaaatatg gcgaggacaa cggaaaaagg tggaaaaTTT AgAAATGTCC  100
ACTgtaggac atggaatatg gtaagaaaac tgaaaatcac ggaaaatgag  150
aaatatccaa ttgatgactt gaaaaatgac gaaatcactg cgaattcaga  200
tctcccgggt caccg
```

Genomic chr3 :

```
aaaggatcct gtgaattagt gagaatatat acacatgaaa atttttagta  162080095
ataatttggg tgacatatgg ttaaaagcca gaataaattg gcagataaca  162080145
AATTCCTTTA GGACATtggt caatcaaatc agaatcccag ggcatgcctg  162080195
ttaaaacaca gatttctatg tcccacacct agagtttctg attttgtaag  162080245
tctagggtgt gtgcagcttg ataatttgga tttctaataa gttttcagct  162080295
ggtgcttatg ctgctgatct gtccttttct ccagtctctc acagtgatac  162080345
aaattgagaa ctgctcctgt aggagaccag agactagaga ggtctgcagt  162080395
atgattgccg tgtgttgaga tgatgcttcc tttcatcatt cacctgctgc  162080445
agttacccat gtttctcttt tctggccctc attttatgac aacagctggt  162080495
agcactcTTT AAAATGTCCA CTtctgtaaa aagtgctacc tggtagcact  162080545
tctgatgtca taagcagaga gcatgccctt ttctataatt aggtaattct  162080595
ctacagagag aggaaattat ac
```

Side by Side Alignment

```
000000037 aattcctttaggacat 000000052
>>>>>>>>> |||||||||||||||| >>>>>>>>>
162080146 aattcctttaggacat 162080161
```

```
000000088 tttagaaatgtccact 000000103
>>>>>>>>> |||| ||||||||||| >>>>>>>>>
162080503 ttta.aaatgtccact 162080517
```

Alignment of YourSeq and chr2:100335541-100335561

Clone 70 YourSeq

```
cggtgacccg ggagatctga attccgagcc gatgatgccg cccagcaccg  50
acgacaacag gatgattggc aggatgccct tcagtccgca ccaggcgccc  100
agggccgcca gcagcttgaa atcgccGTGG CCCATGCCTT CCTTGCCggt  150
cagctgcttg aacagccacc agaccgtcca cagcgacagg tagcccaccg  200
ccgcgcctac gaattcagat ctcccgggtc acag
```

Genomic chr2 (reverse strand):

```
atggtattta ttaacttgca catcgttatg ctttagcacc tctcatactg  100335612
gtgctccagt gaggggaggg tttagtgttt tattaatgcc tcattcattt  100335562
GTGGCCCATG CCTTCCTTGC Cctcactgct gttacgttat attttgtcac  100335512
tgaatattca tttggcttag cctgcaggtt tatagaatct gctatttaaa  100335462
aggcctcttt cgtcatcaaa a
```

Side by Side Alignment

```
000000127 gtggcccatgccttccttgcc 000000147
<<<<<<<<< ||||||||||||||||||||| <<<<<<<<<
100335561 gtggcccatgccttccttgcc 100335541
```

Alignment of YourSeq and chr17:71582696-71582719

Clone 71 YourSeq

```
cggtgacccg ggagatctga attcgtggtc gaaggcatac agcatccact  50
gcacgtactc ggattcactc acccggccgt cgccatcgct gtcgatccgc  100
tgcaggtagc tggtggtatc ggtgacctgg gccagCGCCG CacccgGCAG  150
CAGCACCGCC AGCAgaactc cgtaccctgg cctcacgcgc ccaccaggct  200
gtagcccttc agccgtgcgg cgtaggcctg caacgccgcg ataccgcttt  250
cctcgggaat tcagatctcc cgggtcaccg
```

Genomic chr17 (reverse strand):

```
gtgtagatgg tggcctggaa gggcacgcag cacaggatga aacacaggtc  71582770
ggccacgccc aggttaagga tgaacaggtt ggtagtgctg accgcctggc  71582720
CGCCGCGCAG CAGCACCGCC AGCAccagcg tgttgcccac ggtgcccacg  71582670
aggaagatga gcgcgaagag caggggcacg atgaccgcct cggggtgcca  71582620
gcctccccg ccgcccgcct ggct
```

Side by Side Alignment

```
00000136 cgccgcacccggcagcagcaccgccagca 00000164
<<<<<<<< ||||||     |||||||||||||||||| <<<<<<<<
71582719 cgccgc.....gcagcagcaccgccagca 71582696
```

Alignment of YourSeq and chr6:163940565-163940587

Clone 72 YourSeq

```
gcggccgctc tagcccgcgg tgacccggga gatctgaatt ctccagcatt  50
tcttgcctcg ccgcgctaag cgccACAGAA GCAGTGGCCG TagttccTCC  100
ATCggatacc attaatctcg ccgcatcaat accttctttc tcatagacac  150
ttttcatgtc cattactttt actttatagt cttcaaaggc gcggatggtt  200
tttttaatta attcaccatc atgttggtct gaaatcagat ttgactggta  250
ttcatttaat gaattcagat ctcccgggtc accg
```

Genomic chr6 (reverse strand):

```
tttctacaag gatggattcc cctccttttt tttcttagca agacgtcatt  163940638
tggaaaggtt ttgagcaatg ggcatcgtct tatcacctgc tgtttggggt  163940588
ACAGAAGCAG TGGCCGTTCC ATCtggtctg gcccccaaat ctctatactc  163940538
cctttgctcc actccacctc ttcctactca ccaggccatt cttttctgag  163940488
ctccctcttt cttttaatat ctt
```

Side by Side Alignment

```
000000075 acagaagcagtggccgtagttcctccatc 000000103
<<<<<<<<< ||||||||||||||||      |||||| <<<<<<<<<
163940587 acagaagcagtggccgt......tccatc 163940565
```

Alignment of YourSeq and chr9:86226366-86226670

Clone 73 YourSeq

```
cggtgacccg ggagatctga attcggatcg gcaggatgta gtACGGGTCC  50
TGTGcagaca ggtcatggat ccacagtgcg aacggcgcgt ggcgcagctc  100
aacgGAACCC ATCAGATGAT ctgccccaag gaagccat
```

Genomic chr9 (reverse strand):

```
ccagttgctc ctatagataa catcactatt gtagaaccta cgattggtct  86226721
tttgagatat tttcagactg acccaacctg gattcatgat tcatgactca  86226671
ACGGGTCCTG TGgtacccac cccccaacca ccccaaggca acctcagtgc  86226621
acaagggcca ttttccacac ccctatgatt gcattcccca gccaatcagc  86226571
agcacccatt ccctagtccc atgcccacca aatcatccct gaaaaatcct  86226521
aactttcaat ccttcaggga gactgatttg agtgataatg ccagttttcc  86226471
acatggtcaa cttcaatgtc aattaatctc ttctttactt cactaccaca  86226421
gtctcagtga attggttttg tctgtgcacc tgtcaggacG AACCCATCAG  86226371
ATGATtacaa gagttgagca catctgggaa attcaaatca acttcctcag  86226321
tccaaagaac agtgggaaca cacagtcagg catggggatc acttcccaca  86226271
agaat
```

Side by Side Alignment

```
00000043 acgggtcctgtg 00000054
<<<<<<<< |||||||||||| <<<<<<<<
86226670 acgggtcctgtg 86226659
```

```
00000105 gaacccatcagatgat 00000120
<<<<<<<< |||||||||||||||| <<<<<<<<
86226381 gaacccatcagatgat 86226366
```

Alignment of YourSeq and chr9:22969261-22969299

Clone 74 YourSeq

```
cggtgacccg ggagatctga attctcttga ggtaaacgta tgtcttaccc  50
gttcctgtct ccatttcgat agtaaaatcg gggaaatcta atttttcgga  100
cacatctaac ccttgctcca tttgaacagt acgcaaatta tcaaggattt  150
tgccttccga tatagttagc ctattgcctt cgccaatgat tgtagcactg  200
tcatcATGTT GAAAGAATTC AGATCTCCCg ggtcaccg
```

Genomic chr9 :

```
gaaaatgact gatttcaggg atgggcaggg aaagtgtgag atgtacctgg  22969210
aaattatttt ggtgccagaa agagaggaga tgctcaaaac tgattaaaat  22969260
ATGTTGAAAG AATTCAGgag acaataggaa aaATCTCCCa ggtgacacat  22969310
atgggacaac ttgagcacaa tttaaaaaat gagggcaacg gattaaaccc  22969360
atagactaag ataaacatct atgtcttcct aataataaa
```

Side by Side Alignment

```
00000206 atgttgaaagaattcag 00000222
>>>>>>>> ||||||||||||||||| >>>>>>>>
22969261 atgttgaaagaattcag 22969277
```

```
00000223 atctccc 00000229
>>>>>>>> ||||||| >>>>>>>>
22969293 atctccc 22969299
```

Alignment of YourSeq and chr1:243752902-243752933

Clone 75 YourSeq

```
cggtgacccg ggagatctga attcgggtct gttcattgca gcgcacccgg  50
atccagccca tgctaaatgc gacggtccat ccgcgctgcc gctggcgaaa  100
ctgGAACAGT TCCTGACGCA GAtTAAAgcc attgatgACC TGGTgaaaag  150
cttcgacgag ctggatactg aaaactaaga gcgttcaaca ggccggggga  200
tattcctccg gaattcagat ctcccgggtc accg
```

Genomic chr1 (reverse strand):

```
ttccttaact ttcctgtgcc acagtttccc cagctgtaaa acaggatggt  243752984
aagagtagct agcctcatgg aattgttgca aagttcaaat aagattatca  243752934
GAACAGTTCC TGACGCAGAg TAAAcACCTG GTaggtgttt gctcccatta  243752884
ttgttaaaat aaaacattaa tatgaaaact atatcctaag tgtctccgtt  243752834
tagacatttt ttcttagcag cttcttagaa ta
```

Side by Side Alignment

```
000000104 gaacagttcctgacgcagattaaa 000000127
<<<<<<<<< |||||||||||||||||| |||| <<<<<<<<<
243752933 gaacagttcctgacgcagagtaaa 243752910
```

```
000000138 acctggt 000000144
<<<<<<<<< ||||||| <<<<<<<<<
243752908 acctggt 243752902
```

Alignment of YourSeq and chr3:72579076-72579098

Clone 76 YourSeq

```
gcggccgctc tagcccgcgg tgacccggga gatctgaatt cggacatgtg  50
ccggaggatg aggggatgat cgaggcggcg atcgccaaag atccggcgcg  100
cttcccgctg atggcgattt gcgccgcaag cggcaagccc gcgcgatctc  150
gctatcgtgt cgttgagcgc ttttatcagG CGGGGGAAGG CGGGGTGGCG  200
atgccgttaa cgcgcgtcgc gctgacgcca gagaccgggc gcacccatca  250
gctgcgcatt cactgccagc tgatggggca tgctatgaat tcagatctcc  300
cgggtcaccg
```

Genomic chr3 :

```
cgccgagtga ctgacgaggg gcggggcctg gcgccgcgcg tcacgcgccg  72579025
tgcggccaat gggagggcgg cggccggcgc cgcgagggcg gaggcggggc  72579075
GCGGGGGAAG GCGGGGcgTG GCGgacgtgg gccgggcggt cggtgtgcgt  72579125
gcccgggagt gtgtgcgcgc tgggggcgcg cgtgcctgtc ggtggccccg  72579175
cggcccggcg cggcggagcg tgc
```

Side by Side Alignment

```
00000180 gcgggggaaggcgggg..tggcg 00000200
>>>>>>>> ||||||||||||||||  ||||| >>>>>>>>
72579076 gcgggggaaggcggggcgtggcg 72579098
```

Alignment of YourSeq and chr2:10927023-10927052

Clone 77 YourSeq

```
cggtgacccg ggagatctga attcgatctg aattctgtcc GCAGACTACA  50
GAGCTCTTct tggCCCCTGA Gcccgcctta gagtgtctcc tttgaccata  100
gttgcactgt cttctatgaa aggagcagtt tgtgggatag ggaccagatg  150
gcagaattca gatctcccgg gtcaccg
```

Genomic chr2 (reverse strand):

```
cataaataag attccacccc aaacctgcct tcttccaaat ctggatgaca  10927103
cttacaccca atctggctgt ctcatatcac ccagcccacc cgtgcattct  10927053
GCAGACTACA GAGCTCTTaa caCCCCTGAG aagggcaaat gcatgccctt  10927003
gtctcccagc aagtctctgg caaagctaag gccagaagtt gcatctccca  10926953
agccccggtc caaagcacgt tcgatcactg
```

Side by Side Alignment

```
00000041 gcagactacagagctctt 00000058
<<<<<<<< |||||||||||||||||| <<<<<<<<
10927052 gcagactacagagctctt 10927035
```

```
00000064 cccctgag 00000071
<<<<<<<< |||||||| <<<<<<<<
10927030 cccctgag 10927023
```

Alignment of YourSeq and chr15:23784105-23784506

Clone 78 YourSeq

```
gcggccgctc tagcccgcgg tgacccggga gatctgaatt cagtttcaat  50
gacgatgagt tgaaattctt aaagcgaaga cattcttcaa aaagagcgct  100
cgatgttgtg aatacatgta aatcgttagg ttataataat atcagtatcg  150
acctgatgta tgGTCTTCCC AACCAGACTA TGGATATTTG gcaacgtaat  200
ttAGATAAgG CCATTTCTct agatagaatt cagatctccc gggtcaccg
```

Genomic chr15 :

```
ctgcagggaa attcagcttc cagagtttct gtcactgaag tgcagcatgg  23784054
cccactcagc catcgaactc ctacctgttt gtttcatttg cattcgcttt  23784104
GTCTTCCCAA CCAGACTgaa aggtcagcca cacacaccca gctcctccca  23784154
cccaaccctc ctggggccca ccccaaagct gagtgtgctt cagttcagta  23784204
catgcagttt ggcataagcA TGGATATTTG cagaatggta tgctcaaacg  23784254
tccttgaaac atcccagtga gaaaggtgtg tgctcccttc actcattaaa  23784304
gtgatacgta atgcttgagg acctagctta gacatggtcc tgtttgtgaa  23784354
agaaatagac attttgtgat ataattatca aaacacaaca ttcggggcta  23784404
gaagtggctt tcaaaatcat ttaaacgaat tttgtaattt cacagataag  23784454
ttcacagtta gctcatggta tttggatata ttacAGATAA tgtGCCATTT  23784504
CTaacagctg ggatctgggg cagccaaacc tgacaacttt ttgcagtgac  23784554
cacataaacc agtctgctgg ccagtggcct acccatgtcc atggagccca  23784604
gg
```

Side by Side Alignment

```
00000163 gtcttcccaaccagact 00000179
>>>>>>>> ||||||||||||||||| >>>>>>>>
23784105 gtcttcccaaccagact 23784121
```

```
00000180 atggatatttg 00000190
>>>>>>>> ||||||||||| >>>>>>>>
23784224 atggatatttg 23784234
```

```
00000203 agataa 00000208
>>>>>>>> |||||| >>>>>>>>
23784489 agataa 23784494
```

```
00000210 gccatttct 00000218
>>>>>>>> ||||||||| >>>>>>>>
23784498 gccatttct 23784506
```

Alignment of YourSeq and chr6_random:936738-936766

Clone 79 YourSeq

```
cggtgacccg ggagatctga attccTGCAG CAGGCTTCCT GTCTCACTTC  50
gtagagaact accatgaagg ctggctgcac attgactgct ccgcaacgta  100
ccgtaaagcg gcggttgagc agtggtctgc gggcgcgacc ggtctgggcg  150
tacgtaccgt tgcgaacctg ctgacggctg agtaatcgtc attgccctcg  200
aattcagatc tcccgggtca ccg
```

Genomic chr6_random (reverse strand):

```
aaaattaatt gaggggctgt gattctctgt ttttataatt caaattagtc  936817
ttttgtccat tcgacctaga agttttctcc ttatataaat taagtaggaa  936767
TGCAGCAGGC TTCCTGTCaa ttTCACTTCt aggaacacca ttattaatta  936717
gccaatgcca gagctctaca tgagtcagac tattctgatt gcagctttcc  936667
ctctgctggc cattacagta gtagctgtg
```

Side by Side Alignment

```
000026 tgcagcaggcttcctgtc....tcacttc 000050
<<<<<< ||||||||||||||||||    ||||||| <<<<<<
936766 tgcagcaggcttcctgtcaatttcacttc 936738
```

Alignment of YourSeq and chr13:83330613-83330637

Clone 80 YourSeq

```
cggtgacccg ggagatctga attcaccgtc tttggtgcac gttcaaacgc  50
cgcggcaatc gcctgatgca attcattctg ggcgataggt ttatgcaggt  100
agttctggat cccgatccgC TTCAGcaTaT TGATATCAAA ATGTCGgctc  150
atggagctca gcatgatgat gtggctctca gcaacctgcg gcagaattca  200
gatctcccgg gtcaccg
```

Genomic chr13 :

```
gcttctgtgt catctggatg atttcttcta taacaataat tttattcaaa  83330562
atattctgta attcctaaat ctatattttt aattcaagcc tctcccctgc  83330612
CTTCAGTcTT GATATCAAAA TGTCGatagt attccaagaa gatttatcac  83330662
ttagcatgca taaaaatgga ctcatcttca cccacaaacc agtgatccat  83330712
ttggtatttt ttgtccaaat tgttg
```

Side by Side Alignment

```
00000120 cttcagcatattgatatcaaaatgtcg 00000146
>>>>>>>> ||||||  | ||||||||||||||||| >>>>>>>>
83330613 cttcag..tcttgatatcaaaatgtcg 83330637
```

Alignment of YourSeq and chr1:243752902-243752933

Clone 81 YourSeq

```
cggccgctct agcccgcggt gacccgggag atctgaattc gggtctgttc  50
attgaagcgc acccggatcc agcccatgct aaatgcgacg gtccatccgc  100
gctgccgctg gcgaaactgG AACAGTTCCT GACGCAGAtT AAAgccattg  150
atgACCTGGT gaaaagcttc gacgagctgg atactgaaaa ctaagagcgt  200
tcaacaggcc gggggatatt cctccggaat tcagatctcc cgggtcaccg  250
```

Genomic chr1 (reverse strand):

```
ttccttaact ttcctgtgcc acagtttccc cagctgtaaa acaggatggt  243752984
aagagtagct agcctcatgg aattgttgca aagttcaaat aagattatca  243752934
GAACAGTTCC TGACGCAGAg TAAAcACCTG GTaggtgttt gctcccatta  243752884
ttgttaaaat aaaacattaa tatgaaaact atatcctaag tgtctccgtt  243752834
tagacatttt ttcttagcag cttcttagaa ta
```

Side by Side Alignment

```
000000120 gaacagttcctgacgcagattaaa 000000143
<<<<<<<<< |||||||||||||||||| |||| <<<<<<<<<
243752933 gaacagttcctgacgcagagtaaa 243752910
```

```
000000154 acctggt 000000160
<<<<<<<<< ||||||| <<<<<<<<<
243752908 acctggt 243752902
```

Alignment of YourSeq and chr22:19678441-19678482

Clone 82 YourSeq

```
cggtgacccg ggagatctga attctggaag gcggcctggc cgcGCTGCTG  50
GccgaaccGG GCCCGGCaCT GCTGCACGTG GCCATCgatg cacgcgccaa  100
cgtgtggccg ctggtgccgc cgaacaccgc caacagcacg atgctggaaa  150
gcaaccccgc ccatgcccgc caggagaccc ccaatgcaat accggcttga  200
cctggtgctg gaattcagat ctcccgggtc accg
```

Genomic chr22 :

```
cagccatgcc tggtgtccac tggggtgtcc ttgagctccc ttctccccac  19678390
agaagctgga gcaggaggcc gccccagttc ccagggaggc ccccggcgtg  19678440
GCTGCTGGtg GGGCCCGGCc gcccCTGCTG CACGTGGCCA TCcgggaggc  19678490
cgaggcccgg cccttcgagg tgctcatgca gttcctctac accgacaaga  19678540
tcaaataccc acggaaaggt ccgcctgggt gggggtggag ca
```

Side by Side Alignment

```
00000044 gctgctgg 00000051
>>>>>>>> |||||||| >>>>>>>>
19678441 gctgctgg 19678448
```

```
00000059 gggcccggc 00000067
>>>>>>>> ||||||||| >>>>>>>>
19678451 gggcccggc 19678459
```

```
00000069 ctgctgcacgtggccatc 00000086
>>>>>>>> |||||||||||||||||| >>>>>>>>
19678465 ctgctgcacgtggccatc 19678482
```

Alignment of YourSeq and chr1:112991110-112991132

Clone 83 YourSeq

```
cggtgacccg ggagATCTGA ATTCGTCTTT CAcAAACtcc aggttgtcca  50
gatcaaaacg gtgcatcacg tagaagtagc cgtttttcgc catccactcg  100
cagatccgct catccaccac cgtcttcatg ttggcgggca cgaccggcag  150
gcgaaaacgc cgtccgccca gctccacgcc ggcatcgcat tccgatcgga  200
attcagatct cccgggtcac cg
```

Genomic chr1 (reverse strand):

```
actgtttagg agtcttcaaa ttttcttttc ataaagcacc ccaaagctgt  112991183
tactatcaat ataggtacat tctgatttgg gaatatacgt gttttgataa  112991133
ATCTGAATTC GTCTTTCAaA AACaggataa tagtttttga ccaaatacat  112991083
tacattcaca aaaagagaaa gctatgatct tgattatgaa catttaagca  112991033
aaactaaata aaatattagc aaa
```

Side by Side Alignment

```
000000015 atctgaattcgtctttcacaaac 000000037
<<<<<<<<< ||||||||||||||||| |||| <<<<<<<<<
112991132 atctgaattcgtctttcaaaaac 112991110
```

Alignment of YourSeq and chr11:1184967-1185236

Clone 84 YourSeq

```
gcggccgctc tagcccgcgg tgacccggga gatctgaaTT CTTCCTGAAC  50
CCTGccgact gccatgaccg ccgctaccga tccGCTGATC TCCCTttcgc  100
actattacct gccggtctac aagccccgcc aggtggtact ggagcgaggc  150
cagggcgccc gcgtgtggga cagccagggc cgtgagttca tcgacctggc  200
cgccggcatc gccggaattc agatctcccg ggtcaccgcg ggc
```

Genomic chr11 (reverse strand):

```
cgggtgccgg gagcaaggcc atagtgcctc ttggctctgg ggacctaagg  1185287
cacatcctac caccagggtg tggcctggtg acctccctgc ccccaggggc  1185237
TTCTTCCTGA ACCCTGtgag ctggagccag cctgccaggg cccctcccag  1185187
caggacgtgg attctcagtt ttcactaaat tctgaaccat cctttcgtgg  1185137
gagggtagaa gcttgagagc catcgaccgg ggacatcagc ttgggggccc  1185087
ttccctggca ccctcctgcc agcccccgcc caggcatctg aggcctccag  1185037
ggtgggtgct gggcgaggca gcctgagccc ccggggcctg caggggtggt  1184987
gcatcaggGC TGATCTCCCT ctgggtggga cagtcagagg agcagccccc  1184937
caaccctgcc cgggtgggaa tgagcctctg gctcagccca gccagagagg  1184887
agcagtggga cgctggcctg
```

Side by Side Alignment

```
0000039 ttcttcctgaaccctg 0000054
<<<<<<< |||||||||||||||| <<<<<<<
1185236 ttcttcctgaaccctg 1185221
```

```
0000084 gctgatctccct 0000095
<<<<<<< |||||||||||| <<<<<<<
1184978 gctgatctccct 1184967
```

Alignment of YourSeq and chr2:23362288-23362514

Clone 85 YourSeq

```
cggtgacccg ggagatctga attccctccg tattaccgcg gctgctggca  50
cggagttagc cggtgcttct tctgcgggta acgtcaattg ctgtggttat  100
taaccacaac accttcctcc ccgctgaaag tactttacaa cccgaaggcc  150
ttcttcatac acgcggcatg gctgcatcag gcttgcgccc attgtgcaat  200
attccccact gctgcctccc gtaggagtct ggaccgtgtc tcagttCCAG  250
TGTGGCTGGT CATCCtctca gaccaGCTAG GGATCGtcgc ctaggtgagc  300
cgttacccca cctactagct aatcccatct gggcacatct gatggcaaga  350
ggcccgaagg tccccctctt tggtcttgcg acattatgcg gtattagcta  400
ccgtttccag tagttatcga attcagatct cccgggtcac cg
```

Genomic chr2 :

```
ttgaaaatcg aagttactta ggaagctaga aaatggaaat gaccagaacc  23362237
tggtttggag aaaggatgga aaggtacaat caccaaagct aaggtggttg  23362287
CCAGTGTGGC TGGTCATCCc tgccagctgt ggctggaaat tgagactttc  23362337
tagaaccatc attaactccc aggggccgat gagcggatga ctaggtccta  23362387
attggacagt gactgctggc cacacctcat aatctcagga ttatgcctct  23362437
ctctagacaa gaattcgcag tgtactgcat gagcctcctc ctttattaga  23362487
ttgtcaattc cttgagGCTA GGGATCGaga tggattcttc tctttgtctc  23362537
cctcagtccc tgcagaagcc tttaccaagg atcaaagctc aggaaatgtt  23362587
ataggtagat taccacgtct gcttcat
```

Side by Side Alignment

```
00000247 ccagtgtggctggtcatcc 00000265
>>>>>>>> ||||||||||||||||||| >>>>>>>>
23362288 ccagtgtggctggtcatcc 23362306
```

```
00000276 gctagggatcg 00000286
>>>>>>>> ||||||||||| >>>>>>>>
23362504 gctagggatcg 23362514
```

Alignment of YourSeq and chr7:10534236-10534257

Clone 86 YourSeq

```
cggtGACCcG GGAGATCTGA ATTCTGatcg tgacccggaa cggccatcac  50
cgcacctgta ccgtattcca tcagcacgaa gtttgccacc cagacaggga  100
tcgcttcgcc tgtcagcggg tgtttggcat agaagcctgt tgcaacgcct  150
tttttctcca tcgttgccat atcggcttcg gccactttgg tgctgcggca  200
ttcgtcgatg aaggaggcca gttccggatt gttctcggct gctttctgcg  250
ccagcgggtg acctgcggcc acggccaggt agggaattca gatctcccgg  300
gtcaccg
```

Genomic chr7 (reverse strand):

```
cactcatctg ggctacctgg attccccata actaccagga ggagaggcca  10534308
agtctgctgg tccacagaga ctgcagccac ccttccccct aggggctcag  10534258
GACCaGGGAG ATCTGAATTC TGtccctgag cctctggctg tcgttagcct  10534208
cttccctgta gagatcctgc agggaagccc cacccactga tgggtgatgg  10534158
acaagggtta gacctgaaga gg
```

Side by Side Alignment

```
00000005 gacccgggagatctgaattctg 00000026
<<<<<<<< |||| ||||||||||||||||| <<<<<<<<
10534257 gaccagggagatctgaattctg 10534236
```

Alignment of YourSeq and chr1:218492420-218492439

Clone 87 YourSeq

```
gcggccgctc tagccccggt gacccgggag aTCTGAATTC ACTTGCCCCT  50
Tgttgtcgac agggatgtcg gtcgtatctg ctgcttgcaa ctcagcccct  100
cgcgtcgcaa aacgcctgca tccgagtgtt gcggcctgag cttcaatgag  150
attgtcctga cgacgaattc agatctcccg ggtcaccg
```

Genomic chr1 (reverse strand):

```
tcgccccctc cactccctcg tacttttctt cgttctcttt gggtcaagct  218492490
gtttgtccag tcagtcccag tgcaagaacc tgggtatctc agttgaaggt  218492440
TCTGAATTCA CTTGCCCCTT ttcatttctc tccataagct ccacagacca  218492390
cagctgcttc taatcgtcca tttcaggtct gagacttctt tcagactttc  218492340
tgtgctcagt caacacacac
```

Side by Side Alignment

```
000000032 tctgaattcacttgcccctt 000000051
<<<<<<<<< |||||||||||||||||||| <<<<<<<<<
218492439 tctgaattcacttgcccctt 218492420
```

Alignment of YourSeq and chr12:39485907-39486021

Clone 88 YourSeq

```
cggtgacccg GGAGATCTGA ATTctggttg tagattatac agatcttatt  50
tctacataca aaactcttgc tatacaGGTT GCTGTTGCTT GTgatccgct  100
ggctttggtt aaattaaaat ctccagctga aatgggtgca gattgtgctg  150
ttggtacatc tcagcgtttt ggtgttccta tgggatacgg aggtcctcat  200
gcagctttct ttacttgtaa agaggattat aagagagata ttccgggaag  250
aattattggt gtttctcagg atatgtatgg taagcctgca ttaagaatgg  300
ctttacaaac aagagagcag catattaagc gtgaaagagc gacatctaac  350
atttgtacag gaattcagat ctcccgggtc accg
```

Genomic chr12 :

```
gaatgctaaa cattgtgtat ggaacagtag aggctgaggt caatggtatt  39485856
tatacctgaa aatgagcagt ccccttctat tgggttgtta gtgtgtggaa  39485906
GGAGATCTGA ATTtttagtt ttttcttacc ctggctatct tcaatgggac  39485956
acaggcttca aatttctcta gcgtggcctg atgcttagag agaaggctgG  39486006
GTTGCTGTTG CTTGTttgtt ttctttccta ttttttaaat caatgttgta  39486056
atctgctccc agctctccgc tgactctatg tgccctttgc acagatgata  39486106
tctctctcca tgttc
```

Side by Side Alignment

```
00000011 ggagatctgaatt 00000023
>>>>>>>> ||||||||||||| >>>>>>>>
39485907 ggagatctgaatt 39485919
```

```
00000077 ggttgctgttgcttgt 00000092
>>>>>>>> |||||||||||||||| >>>>>>>>
39486006 ggttgctgttgcttgt 39486021
```

Alignment of YourSeq and chr14:20568723-20568742

Clone 89 YourSeq

```
cggtgacccg ggagatctga attcaatgcg gtttacctta tcgtgtcGAA  50
CACGATGTCC ACGTCCGccc cgccatgagc acctacgacg ccaccctgcc  100
ctacccgcgt gacctgatcg gctacggccg caacccgccg catgcgcaat  150
ggcccggcgg cgcccgcatc gcggtgcatt tcgtgctcta ttacaaggag  200
aattaagatc tcccgggtca
```

Genomic chr14 (reverse strand):

```
actgcaaatg ttccaccacg tgaacccttc cgtggccaac tacctcgcag  20568793
gttcttctca cccccacctc catatttttg gctcctcact tgactttgct  20568743
GAACACGATG TCCACGTCCG tggaggtgac tgtcttgcca tccatgatgc  20568693
cacagtcttt gcacagcttg gagaagttct tgttgttcat ttcagtgcca  20568643
ctgcttgatg attctccaaa
```

Side by Side Alignment

```
00000048 gaacacgatgtccacgtccg 00000067
<<<<<<<< |||||||||||||||||||| <<<<<<<<
20568742 gaacacgatgtccacgtccg 20568723
```

Alignment of YourSeq and chr5:8830497-8830517

Clone 90 YourSeq

```
cggtgacccg ggagatctga attccagcgc accgtatctt gacgatgcgg  50
aaattgactt cgtcaccgat cagctgggtt ctcagctgac cctgaaagca  100
cccaacgcaa aaatgcgtaa agtgtctgac gatgccccgc tgatggagcg  150
cgttgagtac ctgctgcaat cccagatcAA CCCACAGCTG GCAGGTCACg  200
gcggccgtgt ctcactgatg gaaatcactg aagatggcct ggcgatccga  250
attcagatct cccgggtcac cg
```

Genomic chr5 :

```
aactgtgcat atatggagga agagaagttc attggaagga atggcaagaa  8830446
caagaacatt taggcaggat ggagctgggc atgatttaag acagaaagga  8830496
AACCCACAGC TGGCAGGTCA Caacaggtag tgggatgctt tcaaacaatc  8830546
aggtagacag gtgaggttgc atggctcctt taagactatg caaatgcaaa  8830596
gagtgctggc ttctgtagaa g
```

Side by Side Alignment

```
0000179 aacccacagctggcaggtcac 0000199
>>>>>>> ||||||||||||||||||||| >>>>>>>
8830497 aacccacagctggcaggtcac 8830517
```

Alignment of YourSeq and chr7:20343309-20343346

Clone 91 YourSeq

```
ccgggagatc tgaattcggc cacaagagAA ATATAAAAAG TAATCCagcc  50
actaaatggt tgacctataa tgtgtcattc ttataagata tgctagaaca  100
aacatggcac agaatttctg ggagtagcca atcaatatct gatttgactt  150
aacacccagt caacaagtag gaaacaaTAC CCTTGGTATg gtaaggaaga  200
accagaaaca agacccagaa ttcagatctc ccgggtcacc g
```

Genomic chr7 :

```
caaagattaa aagctgagac ttagtactct ttaatcttat ttttcaaagt  20343258
ctgtcttgtt ttcaaacttt ctgtgacaat gatttctatt gttgataata  20343308
AAATATAAAA AGTAATCCta attcttTACC CTTGGTATca actttaaaat  20343358
tgattttaca attttacaaa attgtaatta gattacaaaa actggaattt  20343408
gtagtcattt tcaactaaat acttcaaaaa ttacaaaa
```

Side by Side Alignment

```
00000029 aaatataaaaagtaatcc 00000046
>>>>>>>> |||||||||||||||||| >>>>>>>>
20343309 aaatataaaaagtaatcc 20343326
```

```
00000178 taccccttggtat 00000189
>>>>>>>> |||||||||||| >>>>>>>>
20343335 taccccttggtat 20343346
```

Alignment of YourSeq and chr11:1184967-1185236

Clone 92 YourSeq

```
cggccgctct agcccgcggt gacccgggag atctgaaTTC TTCCTGAACC  50
CTGccgactg ccatgaccgc cgctaccgat ccGCTGATCT CCCTttcgca  100
ctattacctg ccggtctaca agccccgcca ggtggtactg gagcgaggcc  150
agggcgcccg cgtgtgggac agccagggcc gtgagttcat cgacctggcc  200
gccggcatcg ccggaattca gatctcccgg gtcaccgcgg gc
```

Genomic chr11 (reverse strand):

```
cgggtgccgg gagcaaggcc atagtgcctc ttggctctgg ggacctaagg  1185287
cacatcctac caccagggtg tggcctggtg acctccctgc ccccaggggc  1185237
TTCTTCCTGA ACCCTGtgag ctggagccag cctgccaggg cccctcccag  1185187
caggacgtgg attctcagtt ttcactaaat tctgaaccat cctttcgtgg  1185137
gagggtagaa gcttgagagc catcgaccgg ggacatcagc ttgggggccc  1185087
ttccctggca ccctcctgcc agcccccgcc caggcatctg aggcctccag  1185037
ggtgggtgct gggcgaggca gcctgagccc ccggggcctg caggggtggt  1184987
gcatcaggGC TGATCTCCCT ctgggtggga cagtcagagg agcagccccc  1184937
caaccctgcc cgggtgggaa tgagcctctg gctcagccca gccagagagg  1184887
agcagtggga cgctggcctg
```

Side by Side Alignment

```
0000038 ttcttcctgaaccctg 0000053
<<<<<<< |||||||||||||||| <<<<<<<
1185236 ttcttcctgaaccctg 1185221
```

```
0000083 gctgatctccct 0000094
<<<<<<< |||||||||||| <<<<<<<
1184978 gctgatctccct 1184967
```

Alignment of YourSeq and chr1:112991110-112991132

Clone 93 YourSeq

```
gcggccgctc tagcccgcgg tgacccggga gATCTGAATT CGTCTTTCAc  50
AAACtgcagg ttgtccagat caaaacggtg catcacgtag aagtagccgt  100
ttttcgccat ccactcgcag atccgctcat ccaccaccgt cttcatgttg  150
gcgggcacga ccggcaggcg aaaacgccgt ccgcccagct ccacgccggc  200
atcgcattcc gatcggaatt cagatctccc gggtcaccg
```

Genomic chr1 (reverse strand):

```
actgtttagg agtcttcaaa ttttcttttc ataaagcacc ccaaagctgt  112991183
tactatcaat ataggtacat tctgatttgg gaatatacgt gttttgataa  112991133
ATCTGAATTC GTCTTTCAaA AACaggataa tagtttttga ccaaatacat  112991083
tacattcaca aaaagagaaa gctatgatct tgattatgaa catttaagca  112991033
aaactaaata aaatattagc aaa
```

Side by Side Alignment

```
000000032 atctgaattcgtctttcacaaac 000000054
<<<<<<<<< ||||||||||||||||| |||| <<<<<<<<<
112991132 atctgaattcgtctttcaaaaac 112991110
```

Alignment of YourSeq and chr10:70924803-70924826

Clone 94 YourSeq

```
gcggccgctc tagcccgcgg tgacccggga gatctgaatt cggaagcaaa  50
gccatagcat cgctggtggg gttggccccg atgaaccgcg aaagtggcac  100
ctggcagggc caacggcgga tcagtggtgg gcgcgcagta gtacgcgaag  150
ccctttacat ggcggccctg acggccatcc ggtacgaGCC AGGACTGGAA  200
TTCAGaTCTC Ccgggtcacc g
```

Genomic chr10 (reverse strand):

```
cctctccaca ctcctccacc atccagcctc ttgttttaca gatgaggagc  70924877
tcgagacttg gggagggagt gaggtcacag agcaggctgg gtggggtctt  70924827
GCCAGGACTG GAATTCAGgT CTCCtgacct ccctcatggg ctgttctcct  70924777
catcactgca gcactcggtc cccctcggct agggctggga caatccctgc  70924727
cgggaggaac atggcaggcc gccc
```

Side by Side Alignment

```
00000188 gccaggactggaattcagatctcc 00000211
<<<<<<<< ||||||||||||||||| ||||| <<<<<<<<
70924826 gccaggactggaattcaggtctcc 70924803
```

Alignment of YourSeq and chr14:50668000-50668127

Clone 95 YourSeq

```
gcggccgctc tagcccgcgg tgacccGGGA GATCTGAATT CTGgccagtt  50
caacccACAG GGTTTCgtta accagacgca cctcaatcaa ctccagcgcg  100
tcatctaccg acagcggcgc aaccatcacc cctttgcgcg gaataatctg  150
cagcagcccc tcggttgaaa ggcgatgaat cgcctgattg accggcgtgc  200
ggcccatggt taaatcctcc atgaattcag atctcccggg tcaccg
```

Genomic chr14 (reverse strand):

```
atctggtctg cccagattcc tcagaactac caggaggagt ggttaagtct  50668178
gctagtctgc agagactgtg gccacccttc cccctagagg ctcaggccca  50668128
GGGAGATCTG AATTCTGtcc ctgagcctct ggctgggatt attggagatc  50668078
ctgcagggaa gccccaccca ctaaggaagg atgggtcagg gttacgcctg  50668028
aagaggcttg tagtagagAC AGGGTTTCac catgttggcc aggctggtgt  50667978
aatactccta acctcaagtg ctctgcccac ctcggactct gaaagtgctg  50667928
gattgcaggc attagccgcc actcccag
```

Side by Side Alignment

```
00000027 gggagatctgaattctg 00000043
<<<<<<<< ||||||||||||||||| <<<<<<<<
50668127 gggagatctgaattctg 50668111
```

```
00000057 acagggtttc 00000066
<<<<<<<< |||||||||| <<<<<<<<
50668009 acagggtttc 50668000
```

Alignment of YourSeq and chr6_random:936738-936766

Clone 96 YourSeq

```
cggtgacccg ggagatctga attccTGCAG CAGGCTTCCT GTCTCACTTC  50
gtagagaact accatgaagg ctggctgcac attgactgct ccgcaacgta  100
ccgtaaagcg gcggttgagc agtggtctgc gggcgcgacc ggtctgggcg  150
tacgtaccgt tgcgaacctg ctgacggctg agtaatcgtc attgccctcg  200
aattcagatc tcccgggtca ccg
```

Genomic chr6_random (reverse strand):

```
aaaattaatt gaggggctgt gattctctgt ttttataatt caaattagtc  936817
ttttgtccat tcgacctaga agttttctcc ttatataaat taagtaggaa  936767
TGCAGCAGGC TTCCTGTCaa ttTCACTTCt aggaacacca ttattaatta  936717
gccaatgcca gagctctaca tgagtcagac tattctgatt gcagctttcc  936667
ctctgctggc cattacagta gtagctgtg
```

Side by Side Alignment

```
000026 tgcagcaggcttcctgtc....tcacttc 000050
<<<<<< ||||||||||||||||||    ||||||| <<<<<<
936766 tgcagcaggcttcctgtcaatttcacttc 936738
```

Alignment of YourSeq and chr1:32245826-32245851

Clone 97 YourSeq

```
cggccgctct agcccgcggt gacccgggag atctgaattc taacttgttg  50
acatgggtga tcgttccggc gatctcttga ttagtagtaa tcggtttgat  100
cgtcacggcc gcatcttgat gcacttgcgg atagtcatat tcagaagcct  150
tcgcctcgac tgttacttcg ttgctgacaa ttTTTACAGC gggaaCTGGA  200
ATTCAGATCT CCcgggtcac cg
```

Genomic chr1 (reverse strand):

```
ctgagtattt gggaagtggc caggtggagg gagggccagg gcatttaaat  32245902
gttgggagtt cagcaaagct tggtcctgtg ccggtactcc ttcctccatg  32245852
TTTACAGCtC TGGAATTCAG ATCTCCaact caaacttctg aattccaaat  32245802
ccagaaagcc aattgcatcc ctgacatcct catctggatg tctcagaggc  32245752
tcctcaaact caacatgtcc ccagta
```

Side by Side Alignment

```
00000183 tttacagc 00000190
<<<<<<<< |||||||| <<<<<<<<
32245851 tttacagc 32245844
```

```
00000196 ctggaattcagatctcc 00000212
<<<<<<<< ||||||||||||||||| <<<<<<<<
32245842 ctggaattcagatctcc 32245826
```

Alignment of YourSeq and chr16:24411988-24412236

Clone 98 YourSeq

```
cggccgctct agcccgcggt gacccgggag atctgaattC CTTGGCATCC  50
gtcgcaatga acTGAAGGTG GAGGAGATcc cacgggatga catcatacgg  100
gcagcggcat atttcagaga cagtccggaa aaagcacgaa agcgatttgg  150
tgaagaggga tggtacgtca ataaggtgta tgacgcgccg caggccgtaa  200
tggcgctcct gtgccacgga aaaaacctcg gctgggacaa atcgctaagg  250
gaggtgctgc acgtgtttta gaattcagat ctcccgggtc accg
```

Genomic chr16 :

```
cgggcttgag ccaccatgcc cagctattta tttatttttg tggagacagg  24411937
gtctcaccat tttgcctagg ctcatcttaa acggctcaag caatctccca  24411987
CCTTGGCATC Ccaaagtgct gagattacag gcatgtccca ccacgccggc  24412037
ctctctttac ctttctaatg tggctagcag aaagtttaaa attacatgtg  24412087
tgggtcagat gacatttctc ttgggcagca cttccctgga caggcagtgt  24412137
atcccagaaa ggcagacctg ggctagccac agaatcttca accccatccc  24412187
caagacttga ggctgacaag aagtaaaaca aagTGAAGGT GGAGGAGATg  24412237
aaaaatggga tgcaaagaaa cagatgtgat atagtccatc tttctctctc  24412287
ttttttaaaa tatagactgg gtctcactgt gttgcccagg ctggtctca
```

Side by Side Alignment

```
00000040 ccttggcatcc 00000050
>>>>>>>> ||||||||||| >>>>>>>>
24411988 ccttggcatcc 24411998
```

```
00000063 tgaaggtggaggagat 00000078
>>>>>>>> |||||||||||||||| >>>>>>>>
24412221 tgaaggtggaggagat 24412236
```

Alignment of YourSeq and chr6:8509799-8509821

Clone 99 YourSeq

```
cggtgacccg ggagatctga attcccagag accgacgcga gagtctcacc  50
agaaagcccg aggagcttgg caagtttcgg ctcaaccttt gcaccaacgt  100
tcgatgctgg aagaagcccg ggtagccaag GCTTGCCtgc ctgatctaga  150
ACATGGGAGA TGTTCTgcat gctgcactcc cacgaattca ggatctcccg  200
ggtcacag
```

Genomic chr6 :

```
tacatttttt gtttaatttt ctgtcttctc aatgaagaat gaatatctat  8509748
gtcatagact tttctttatt catggctgtc tttataacac ctaggatggt  8509798
GCTTGCCACA TGGGAGATGT TCTagtagtt ttgaatgaat aaataaatga  8509848
atgagctact ttttatatct ttttgatgct ttagtctttt tatatctatt  8509898
tgtatgactt tttaatatta acc
```

Side by Side Alignment

```
0000131 gcttgcc 0000137
>>>>>>> ||||||| >>>>>>>
8509799 gcttgcc 8509805
```

```
0000151 acatgggagatgttct 0000166
>>>>>>> |||||||||||||||| >>>>>>>
8509806 acatgggagatgttct 8509821
```

Alignment of YourSeq and chr18:52432453-52432884

Clone 100 YourSeq

```
ATATTgAAAA ATaAATCTGT CCTTCCTCTT TAGGGTACtT AAATTAAAAA  50
GAAcAAAATT TcTCCCATaA AACATTTTAC TCATTTCAgA CTGAGTGAAT  100
AAtTcCCACT TTAAAGAAcC ACTTAGACTT ACCTAGGATA CTGAAAATAG  150
aATTGTTACT CACAAACCTA TcAAATACAT ATaAACGAAA TACTACTTgC  200
AGTTACAtgT GTTAACATTC TGAAACTTAA CATAACGAAG TGGAACAgTG  250
CCATCTTCTT TAATATCATC CTCTGTCAaT TCCAGAGgTT GAGTTGGTTC  300
ACTcTCTTTC TGCCTCTTCA AAATCCATAG ATCGGGGggt GTTGATAAAA  350
ATTTTTACAT ATTTAGGGCC CTGACCTATA CAATGGTAGA ATAAAATAAA  400
ATGTTTAAAA ACCaCAtATA TACTATtCTC CCTT
```

Genomic chr18 :

```
gaataaagca caaatatctt cagttttagt ataaagttgt agaataaact  52432402
tccagcaaaa cttaccatat acatataaaa taactgacaa aaccccacaa  52432452
ATATTcAAAA ATcAATCTGT CCTTCCTCTT TAGGGTACaT AAATTAAAAA  52432502
GAAgAAAATT TaTCCCATtA AACATTTTAC TCATTTCAaA CTGAGTGAAT  52432552
AAaTaCCACT TTAAAGAAtC ACTTAGACTT ACCTAGGATA CTGAAAATAG  52432602
tATTGTTACT CACAAACCTA TtAAATACAT ATgAACGAAA TACTACTTaC  52432652
AGTTACAcTG TTAACATTCT GAAACTTAAC ATAACGAAGT GGAACAaTGC  52432702
CATCTTCTTT AATATCATCC TCTGTCAgTT CCAGAGcTTG AGTTGGTTCA  52432752
CTTCTTTCTG CCTCTTCAAA ATCCATAGAT CGGGGtagGT TGATAAAAAT  52432802
TTTTACATAT TTAGGGCCCT GACCTATACA ATGGTAGAAT AAAATAAAAT  52432852
GTTTAAAAAC CtCAaATATA CTATaCTCCC TTtaccacta cactcacact  52432902
atacacagac acacacaaat caatacacat atacttcaaa taactccatt  52432952
attagaactt gtacaaaagt ctgagtattc ag
```

Side by Side Alignment

```
00000001 atattgaaaaataaatctgtccttcctctttagggtacttaaattaaaaa 00000050
>>>>>>>> ||||| |||||| ||||||||||||||||||||||||| ||||||||||| >>>>>>>>
52432453 atattcaaaaatcaatctgtccttcctctttagggtacataaattaaaaa 52432502

00000051 gaacaaaatttctcccataaaacattttactcatttcagactgagtgaat 00000100
>>>>>>>> ||| ||||||| |||||| |||||||||||||||||||| ||||||||||| >>>>>>>>
52432503 gaagaaaatttatcccattaaacattttactcatttcaaactgagtgaat 52432552

00000101 aattcccactttaaagaaccacttagacttacctaggatactgaaaatag 00000150
>>>>>>>> || | |||||||||||| ||||||||||||||||||||||||||||||| >>>>>>>>
52432553 aaataccactttaaagaatcacttagacttacctaggatactgaaaatag 52432602

00000151 aattgttactcacaaacctatcaaatacatataaacgaaatactacttgc 00000200
>>>>>>>>  |||||||||||||||||||| |||||||||| |||||||||||||||| | >>>>>>>>
52432603 tattgttactcacaaacctattaaatacatatgaacgaaatactacttac 52432652

00000201 agttaca 00000207
>>>>>>>> ||||||| >>>>>>>>
52432653 agttaca 52432659
```

```
00000210 tgttaacattctgaaacttaacataacgaagtggaacagtgccatcttct 00000259
>>>>>>>> |||||||||||||||||||||||||||||||||||||| ||||||||||| >>>>>>>>
52432661 tgttaacattctgaaacttaacataacgaagtggaacaatgccatcttct 52432710

00000260 ttaatatcatcctctgtcaattccagaggttgagttggttcactctcttt 00000309
>>>>>>>> |||||||||||||||||| ||||||||| ||||||||||||||| ||||| >>>>>>>>
52432711 ttaatatcatcctctgtcagttccagagcttgagttggttcact.tcttt 52432759

00000310 ctgcctcttcaaaatccatagatcggggggtgttgataaaaatttttaca 00000359
```

```
>>>>>>>> ||||||||||||||||||||||||||||   ||||||||||||||||||| >>>>>>>>
52432760 ctgcctcttcaaaatccatagatcggggtaggttgataaaaatttttaca 52432809

00000360 tatttagggccctgacctatacaatggtagaataaaataaaatgtttaaa 00000409
>>>>>>>> |||||||||||||||||||||||||||||||||||||||||||||||||| >>>>>>>>
52432810 tatttagggccctgacctatacaatggtagaataaaataaaatgtttaaa 52432859

00000410 aaccacatatatactattctccctt 00000434
>>>>>>>> |||| || ||||||||| ||||||| >>>>>>>>
52432860 aacctcaaatatactatactccctt 52432884
```
