# Supplementary material for: Hepatoma derived growth factor binds DNA through the N-terminal PWWP domain
Source: BMC Mol Biol. 2007 Oct 31;8:101. doi: 10.1186/1471-2199-8-101 (PMC2176068; doi:10.1186/1471-2199-8-101)
Supplement: Additional file 2 — The alignment of binding core in 5 candidate gene promoters. The data shows the location of binding core in the promoters of 5 candidate genes, and their alignment. [file 1471-2199-8-101-S2.pdf]

Additional Data 2:

The alignment of binding core in 5 candidate gene promoters: T-COFFEE program (http://www.ch.embnet.org/software/TCoffee.html) was used to align the 5 candidate sequences. T-COFFEE report is shown below.

```
=====================================================
T-COFFEE, Version_1.41(Fri Jun 28 14:24:48 MDT 2002)
Notredame, Higgins, Heringa, JMB(302)pp205-217,2000
CPU TIME:0 sec.
SCORE=62
*
 BAD AVG GOOD
*
SMYD1    :  62
ACVRL1   :  58
MCM5     :  65
VEGF     :  63
SIN3B    :  63



SMYD1    CTTGAACTCCTGACCTCAGATGATCCATGTGCCTCGG-
ACVRL1   CTCGAACTCCTGACCTCAGCTGATCCACCCAC-TCAGC
MCM5     CTTGAACTCCTGACCTCAGGTGATCCACCCGCCTGAG-
VEGF     CTCAAACTCCTGACCTCAGGTGATCCACCCGCCTCAG-
SIN3B    CTTGAACTCCTGGCCTCATGTGATCCACCCACCTCTG-

Cons     **   ******** *****  *******     * *   *

========================================================
```

The promoters of candidate genes: The sequences shown below are 5 prime sequences of 5 candidate genes. All the sequences are derived from UCSC genome browser (http://genome.ucsc.edu). The sequences include 3000 bp 5 prime ends from the first exon, cap letters indicate the first exons. Boxed regions indicate the binding cores.

SMYD1 (NM_198274) at chr2:88148497-88194015 - SET and MYND domain containing 1

```
gagtagctgggactacaggcgtgtgccactacgcccagctaattttgtatttttagtagagacgcggttt
caccatgttaggcaggatggtttcaatctcctggcctcgtgatctgcctgccttggcctctcaaagtgctg
ggattacagggaaaaaaccttttaatatttcctctcctctcctctcctttcctgaaaacccctcccctcc
ccttcacttcgctttccttcccttccctcccctcccctcccttcccttccctcccttcccctcccttcccc
tccccttccctctttttttcctgagacaagagcttgctctgctactcctcccctcccctcccctcccctccc
ctccccccctcccctcccctccccccctcccctcccctccccccctcccctcccctcccccccccccccttcc
ctcttttttttctgagacaagatcttgctctgtcacccaggctggaatgcagtggcatgatcatggctccc
tgcaacctctgcctcatgagctcaagtggtcctcccacctcagcctcctgagtagatgagactacaggcat
gcgccaccatgcctggtttatttttgtatttttggtagagacagggtttcaccatgttgcccaggctagt
ctcaaacccctgggctcaagtgattcacccgcctcggcctcccaaagtgctgggattacaggcatgagcca
ctgcgcctggccttctttctcttttttttttttttttttttttgagacggagtcttgctctgttgcccag
gctggagtgcagtggcgtgatcttggctcactgcaagctctgcctccccggtttacgccattctcctgcct
cagcctcccagtagctgggaccacaggcgccggccaccacgcccagctaatttttttgtatttttagtagag
acggagtttcaccgtgttagccaggatggtctcgatctcctgaccttgtgatccacctgcctcgcctccca
aagtgctgggattacaggcgtgagccactgtgcccggcctctttctcttttttttaactctctgttttgtag
aattgtttttccccttgggagcatgcagtgtttactttttgaggcacctaactttacatgtcttaagttat
tttgctcttcagtgtacatcagaagctgcagagatattctcttgctgtcatggctatgtattcttacctaa
aaatcaggatatatgatcagacaatgaactttcaacatgcttccaagagtcgaaggtgttttgggagattc
aattggttgaagacatgttaaaatttctggtacatcgtgatagtttagggaggataatgtgacagagtaaa
taaaatagaatagctatcacttaatgagtactttatgaagtgctaagtgctgtgttatccccttttacata
ttatctaactctcacaatccagtgaggtagaaattatcacccttgtttcacaggttccatctcaagtggta
gtagcaggcttagaattcaaactcagattcatctgactcagaaaccatacactcagaaaaatccaggaaat
gcccctgcccactgcccccacccccgccgtcacccccacccccaacccccaccaggaaatagatgcatcc
caggaactaaaaatcttacatggaagtcaagccagagctccagatctgcaagtgtatttcttttaaacctc
agtaaaagctgcctgatagccaagaacagtggcatttagttcaacggggattttagattcgtctttccagt
gtcctcactcctgagtaagaagaaggacagagcacgttggtattttcctggcagcagacacatgttaaagg
aaatttgatagaaagatagagatggggggttgctatttttatagcctgccttgtctgtatccagcctgcctc
cagcataccagcatcaccagcagtgctaagaacagggaattccatgctgtggagtagggggtgctgtaaga
gccccaggtgatactggagaggaaaaatgccccccttggagcctaaatagtactgttacgtatatgccaagg
gctcagtttagcttcttcttattatttatttatttatttatttatttatttattaatttttttgttgagacg
gggtcccactctgtcgcccaggctggagtgcaatggcgcgatctcggctcactgcaacctctgcctcccag
gttcaagcaattctcctgcctcagcctcctcagtagctgggattacaggcgcccgccaccacgcccagcta
atttttgtatttttggtagagacggggtttcaccatgttggtcaggctggtcttgaactcctgacctcaga
tgatccatgtgcctcggcctcccaaggtggggattacaggcgtgagccaccgtgcccagccctcagtttag
cttctgctgctaaacagtccacccttgggagagagactgaaaagggtggggcgagggcccatctcacttga
gaaagtgcaagcctgacagctgaagggtcccagagcagcaaatatcaccttgtttctatgggaccattcca
gctgtctgcttttgattgacagaggcatctggctgctaaaaataccctcctgaccaaatgttccctactcc
aactaaagccgaacaggattgttgcagttcagaacttagcacatacagatagcttctgcttgcaatggaac
ctctcaaaacttttcaggccctatgccatattaacccatggtcattttctgttctgtcggctttgctgggt
gatgaatgaaactcttcccagttcacttgttctcttgtctgtcccatccccacctccaggaaaagtagcag
agggggaggtgagggcccagtagctgagagcgagtagggaaggggaggtggcttgtccatttatagtcttg
ctctttgggatgctgaaggtgctgaaatagcaatgacaagagacttggctcagtgttaaataactgccgcg
ctggcctgacagtctctgAGATGACAATAGGGAGAATGGAGAACGTGGAGGTCTTCACCGCTGAGGGCAAA
GGAAGGGGTCTGAAGGCCACCAAGGAGTTCTGGGCTGCAGATATCATCTTTGCTGAGCGGGCTTATTCCGC
AGTGGTTTTTGACAG
```

ACVRL1 (NM_000020) at chr12:50587469-50601120 - activin A receptor type II-like 1

ataagtgtaacaaaacacataaaggagttaaatgctgaaaactacaagaacactgataaataaaagagacc
taaataaatggaaaaaaaatatatcattaagaattttaagatggcaatgacagcgcattaaaccaagcatg
gggccctgtgcatctgcacaggtcacactcccatgaagccagccccgcactagtatcaagagtgatgtttg
agtctgggtggggtggcttatgcctgtaatctcagcactttgggaggctgagtgggtggatcagctgaggt
caggagttcgagaccagcctgtacaacatggtgaaaccccgtctctactaaaaatacaaacattagccagg
cgtggaggcacgcatctgtaatcccagctacttgggagactgagacaggagaatcacttgaacctgggagg
cagacctgagatcacgcaactgcactccagcctgggcgacactgtgagactccatctcaaaaaaaaaagaa
tgatgcttgaacttaatactcatagctggtcctgaaaggtgaaggtgtccattgccctgccttcctgacag
actccatgtcacccaccttccacagatgagaatctgcttgaggtttcaccccacacagaatctccaggcct
ctgcttcctgcagtgttgggaaatccttctgagtatctaaatctaaatctttgtcctttgggcctgtttcc
tcttgttctgtcctcactgaatatggggaacagctggtccctctgagtaattaattcttgtagacttggac
acagtctgtaattcctttcccttttctcattgggatgactctccagggctttctccaactctggatctcc
aactctgacacctcccccaggttctctgtgtgctttggaaggcatggggttcacaagagccccaaagagca
ccagtcaggggccagagggctcagtgctctggggagatggggaagattccagcactgatgaacagactcca
gccccccctgggtcctctcttccagctcccaaaatgtactctatttttatctgtttcacgaacgctggtcc
agatagtcttccatcccccactgactgttagaagtgactctcagcttttgtccatctcgaagtttctgtgc
tcagtgtgcctctcagactaaaggcttcctttgggaagccccgactctcgcttctcaggacagagatccag
gggttgggggaggaaaaggttgaccagaagccatagcggagcagggagagagagtgtgaaagacagacccg
cggccaggctcccagttctccagctcgtagagggcccaagtggccgctataatctgaaagagcagatatcg
taatcccatagtacttcctattggctgcaggacacagttctgtcctgacactgaaatttgggtgtgtcagg
gttctgggaattcacaacgctcacaacttgtgaagcagctgtggggtgggggatggggagggtttcagcag
aggaagtgaggtcagtcaataattgatgcctgtctgagcttttagccattatctcccccagcctctattcc
tgtcaaaaggtggggcggggcaggaggaggggtccctggctcatcttgtagaatccccatattagagtaag
acaccttagaggtctactcctgcttctaatacccacgtctttccaagtgtctctgaggccacccccctcccc
agccttttcatttattcatttaattaacgaacgccttcattgagggcctcctctgagtcaggctcagccag
ccagcatctttgctatgagctgagataagcatcatttccgtctattctcacaaccaccctatgaggctggc
acggtttactatgcctatttagcagatgggggactgaagcatggagaggtgtcactagcctacggtaacac
aaccagcctgcattcctagtaggtagtttgacttcagagtctctgtggataaccaggaggctaggactaag
accagagtcctgcaggtacttagatggttggagcaaagcagggcagtgaggtcagtgctcccagcctgtgc
aggagcatcaggaagagtctgtgtccccctcccctgccggtatgaagccattctgcttccctccccagctg
ccttgtgtcagcagagttccagggaggctccattccccacctctatctaaagctccatttgctggggtggg
ggccctgcctggaaggggaaggtccaaggctgctcccagcgtgtccctccatcctgactgtccctggcggg
gcgggggtgtctttgtcacccagctgcacaacggccaggaagggctcaaaccatcctcagggctaacccaa
ggccgtcctctgggcctgtataccccctgtgctgagtgcggatcgggagaggctgctgaagacaggaggga
caaatgggggacgaaggggcccgagggaggggactgaaggatttgggccaagtcgggagttcccgagggcg
gagtcaaaacgcatctggattttgctagccccaaactctgccctcattgctgcaagcctcctagaccgagg
acccccgggctgagggtggggtaaggataggtagtgtccctccccgtcccacccccgcctgtcccttcctc
ggtggccccttcccggcgccccgattccaggcggcccctccgctgctgccagccgatccccctctacccc
acccactactccggccgccagacgttgcctacagtctcggctctgtctcccacggctgtgggtccggaccc
cacgggaccccctatgggaccccccacaggacccccacggcctgagtccaaggcccgccccctcggggaggcg
gatgtgggaggcccggcccgggtgcgggccagcgacccgggagctgcgggcggctgggaggggaggccgcc
ctgaggggctgggagcggcgcgggggtgggtcccggtcctgcagccccagcgaggggcgagcggcggccag
tcggcgagctgggcaataAGGAAACGGTTTATTAGGAGGGAGTGGTGGAGCTGGGCCAGGCAGGAAGACGC
TGGAATAAGAAACATTTTTGCTCCAGCCCCCATCCCAGTCCCGGGAGGCTGCCGCGCCAGCTGCGCCGAGC
GAGCCCCTCCCCGGCTCCAGCCCGGTCCGGGGCCGCGCCCGGACCCCAGCCCGCCGTCCAGCGCTGGCGGT
GCAACTGCGGCCGCGCGGTGGAGGGGAGGTGGCCCCGGTCCGCCGAAGGCTAGCGCCCCGCCACCCGCAGA
GCGGGCCCAGAG

MCM5 (NM_006739) at chr22:34126128-34150494 - minichromosome maintenance deficient protein 5

tggtgaaactctgtctctactaaaaatacaaaaattaaccgggcatggtggcgcgtgcctgtaatcccagc
taccaggaggctgaggcaggagaactgcttgaacttgggaaacggaggttgcagtgagctgaaattatgcc
actgcactccagcctgggtgataaagcgagaatccgtctcaaaaaaaaaaagtggtaaggacagattttaa
tcagtaatgattattgcaacagggaagagagttcagtatgaactgaactcagctttaatttgtatagaggt
atttaaagggaggttgaccagcctgggcaatatggtaaaaccccatctctacaaatgaataaataaattc
gccgggcatggtggtgaggttgaggtgggaggattgcacgagcctgggaggtcaaggctgcagtgagctgt
gattgcaccaccgcactctagcattggcaacagagaccctgtctcaaaaaaataaataaaaaaaataaagg
aagattgagggaggaaggggtgggcaagtgattagtagagtcagggaagtgaaatattacaaaaggttaag
tcagtgtaaatgcagtgaggctagctgtgtctgccagttggcaatgatcaaagttagaattttgcctccc
acagagcctgggagacagaggtcacatcctcggttgttgcctggaataaacggtaaacatttaccagcagc
cttgagttttctcaggcaggcaccttagcgggggctggctagagtcattttagagatgttagcagctgtta
gaaactgttagtgtttgtttaagtctttttttcttttcttttttttttttttgagattaagtttcactct
tgtcgcccagactggagtgtagtggcacgatctcggctcactgcaacctccgcctcccaggttcaagcgat
tctcctgcctcaggcttccaagtagctgggattacaagcgcctgccaccacgcccggctaagttttgtatt
tttagtagacacagggtttcaccatgttggccaggctggtcttgaactcccgcctcagcctcccaaaatgc
tgggattacaggtgtgagccacggcgcctggactgtcttttttttcttttttagagatgagtctcgctctgt
cgctcaggctgaagtgcagtggtgcaatcaaaactcactgcagtctcaaactcctgggctcaggccatcct
cccaccttggcctcttgagtagctaggacaacaggtgtgctccaccatgcccagctaattaaaaaaaaatt
tcaagccgggcgcagtggctcatgcctgtaatcccagcatcttggaaggctcaggcgggtggatcacctga
ggtcaggagttcaaggctggccaacatggtgaaaccctgtctctactaaaaatacaaaaaattagccaggc
gtggtggcacacacctgtaattccagctacttgggaagctgaggcaggagaattgcttgaacccaggaggt
ggaagttgcagtgagccgagatcgtgacactgcactccagcctgggcaacagagtgagactctgtctcaaa
acaaaaaaaattataaaaaaatttctagagaagcgggatcttgctatgttgcccaggctggcctcaactcc
tggactcaagggatcctctcatcttggtctcctgaagtgctaggattacaggcatgagccacagtgcctat
agatcaaggttgaggcttagaagtgaagggagcccagaggaacctggctagagtttggtcaaggagagtct
ttgtcaccatactacagaaaattaagcagctgttaaacagaatgaggccggtctgggcatggtggctcacg
cctgtaattccagcactttgggaggccgaagcgagtggatcacttgaggccagaagttcgagaccagtgtg
ggcaacatggtgaaaccctgtctccactaaaaatacaaaaattagctgagcatggtggcgcctgcctgtag
tcccagctagttgggaggctgaggcaggagaatcgcttgaaccggggaggcagaagttgcagtgagccgag
attgagccactgcactccatcctgggggacagagtgagactccctctcaacaaataaataaacaaataaaa
gcagtgtgttagggtgcgagaacccgggtcggggacgcgggtgagccgcgcggtaatcgccctgtcctgc
caccagctggaggacaggggctcctcagagcctcaggcccagagaacgggccccacgaccgggaagcgaat
actccacagggcacagtctcccaatgcaaaatgggcacctggacccaatcatttctgaacttctagttttt
tctccctatggctatgaaaagggtcatttttttttcataggactttaaaagaaggcttaagaaaagcacgca
ggcctggccgggtagctcacgccagtaatcccagtgctttgggacgtcaaggcaggcgggtcgcttgagag
tagagtttcgagacctgggcaagatggtgagactccgtctttatgaaatattttaagaaaatcacgtacac
ctgtggtccccgctacaagggaggctgaggcggaaggattgcttgagcccaagaggttgaggctacagtaa
gccgtgatccagccactgcactccatccccggcaacacaacgagaccatgcgtcaggaaaaaaaaaaaaaa
accaagaaaataaaaaagaaaccacctaaaggcgtcctccgcgcgcccgcccctccgccatctttgcgtcc
gccccgtcctgcgccgccacttccttcccagccagaagtttcgcgccaaattgttccgcacacaaaatgga
gcccggaaggaggcgaggtcatgcactttgcagccaatcatcttcggcagacagtccgggcggcggggctg
gagggctcagaacggtccaatcgctggccgcgaggctccggcgaaccaatgggagcgcagcggctgcggcg
tccgaacaccgcctcttgtTTTTCCCGCGAAACTCGGCGGCTGAGCGTGGAGGTTCTTGTCTCCCCTGGTT
TGTGAAGTGCGGAAAACCAGAG

VEGF (NM_003376) at chr6:43845931-43862199 - vascular endothelial growth factor isoform b

ggtgtggcccagaaggcagggcagagctagcttctgggttccttcagcccctgcaaggtttgggactctgt
ccacaggtccagacccagtacttattatgaactgttgggggtgcatcctggtgccagcactgaggaagtta
aacgagctttcttctccaagtccaaagaggtacccgtaccccctgaggtggggaggggagcaggaactttg
agccagtgggggtgtgcttcaaattcccaggagtagaccagcatctctggcgggtgccacatttttcagc
cctcatagggagtggcaggtgctcaatggtaggggaaaggctgtgggcaggagccaggggtcctgtctgat
gggctgggcttggtttcttgacctgcctgcctgctcttgagaaacctggaccagggcatatggtgaatgc
agggggctggcaggtggggagggcttgaacacaggaggaagcaggagtccatgctctctggccttctgagg
agtgggcaggcctgagtgagggtcactgaccaggcagggcctctgggtatgactggagccctggtgggtgg
atgtcatcagcatgggtcctggggagggacaggcaaagggagataaggggagtcctgccccaccccagctg
cacccagaccgggaccctgggaacccaagcctgcacagccgctttgtggagctgagcgaggcataccgtgt
gctcagccgtgagcagagccgccgcagctatgatgaccagctccgctcaggtagtcccccaaagtctccac
gaaccacagtccatgacaagtctgcccaccaaacacacaggtacagtaatcctgccctcagcttgccccac
ccccaggtctcttgggtagtctcatttccacaatgtcccttcctctgcctcccagcagctcctggacaccc
cccaacgcacagtactggtcccagtttcacagcgtgaggccacaggggccccagttgaggcagcagcaaca
caaacaaaacaaacaagtgctggggtactgcctcctcctcatgctggcgggcatgggcctgcactacattg
ccttcaggtgatgcctgttctccccggggtgatgggcagagggcaggagggtgggtgaattccagtgtggt
cagccagtcctccacagcacctcgtggcccgtactcttgtgtctctcaagctaagagctgcttgaagttgg
tctctggagcctctccttacttattaaaggcagcgctgcaccaagagctaacgttgtcatttacagtttt
catatttcgaatgtaaatctttcatgctggctgccaggaagggattgagccaaacaaaatcaagcacaagc
agcaaatgcagcacagctcattcatttggcagtaggcatctccctgccactctcacgcgtgccacggagct
gattctctttattctctttttttcttttttttttctttgagatggagtctcactctgttgcccaggctgca
gtgcagtggcgcaatctcggctcactgcaaccccacctctgcctcccaggttcaagcaattcttctgtctc
agcctcccgagtagctgggactacaggcgtccaccaccacacccggctagttgttttgtatttttagtaga
gatggggtttcaccatattggccaggctggtctcaaactcctgacctcaggtgatccacccgcctcagcct
cccaaagtactgggattacaggtgagagctaccgcgcctggccacaaagccgattttctatagcagatacc
taaaggccagtgcccactgctagcagcctgcctagttcagtgatggagttagacgcataccctttctagtc
ttctgggaggagcacccccatgtccacagctgctgtcacacctcaaagtgaccagggacttcggatgccat
tggaccctagacaagccagggccactctgagccctccctatctatcacaggaagggttagactgaggttcc
taaccctgtgtaagggtaaaaagctgtgaggcccctagagtggggtggaagggggcaggacttcagggaca
gcaggaattggattcccagggttaattgtgacccattgcaggaaggtgaagcagatgcaccttaacttcat
ggatgaaaaggatcggatcatcacagccttctacaacgaagcccgggcacgggccaggtctgtccctgctc
tattctgctccctgctccctgtccaggaaccacacttcgggatccctatcccaaccacccaggtgccctct
cctccagggccaacagaggcatccttcagcaggagcgacaacggctagggcagcggcagccgccaccatcc
gagccaacccaaggccccgagatcgtgccccggggcgccggcccctgaggggctcacctggatggggcctg
cagtgcgttcccgctttgcttccttccctggacggcccgctccccgaaacgcgcgcaataaagtgattcgc
agagctcgtgtccggctccctccttaaggcccgacgcccccggccccggcctcgccaaggggcagcgcccc
gccctccgggtagtggcggcggcgactggggagcccagcctcctgggcggtgcgtcccctttcccctgccg
cggcgggaggcgggagggggtgtgtggaggaggcgggccccgccgacggcctcgcccccccaccccgccgc
cccgcccccgccccacgggccggtggggagcgcgtgtctgggtcacatgagccgcctgcccgccagcccgg
gcccagcccccgccgcccccgccgtccccgccgccgctgcccgccgccaccggccgcccgcccgcccggc
tcctccggccgcctccgctgcgctgcgctgcgctgcctgcacccagggctcgggagggggccgcggaggag
tcgcccccgcgcccggcccccgcccgccgcgcccggcccgcgccatggggctctggctgtcgccgcccc
ccgcgccgccgggctaggGCGATGCGGGCGCCCCCGGCGGGCGGCCCCGGCGGGCACCATGAGCCCTCTGC
TCCGCCGCCTGCTGCTCGCCGCACTCCTGCAGCTGGCCCCCGCCCAG

SIN3B (NM_015260) at chr19:16801218-16852164 - SIN3 homolog B, transcription regulator

tgtgagcaaacattcaggtgattccagcccccagactttgagccacccagctgatgccaaggttgagttat
tcctcccaagcctgcccaaattgcagattgaagagtaaaatgaatgtctcttttctttcttttttttttt
tttcaagacagggtcttgctcttgctctgttccccaggctggagtgcagtggcgcaatctcagctcattgc
aacctccaccttctgggttcaagcgatactcatgcctcaacctcccaagtagctgggattacaggtgtgtg
ccaccacacctggctaattattgtatttttagtagagatgggatttcaccatcttgtccaggctggtcttg
aactcctggcctcatgtgatccacccacctctgcctcccaaagtgctgggattacagccatgagccacttg
ccccgtgaatgttgttgtcatttgaagcttctaagtcttgagctgcttttttatttcttttacagaaagcc
cagaccttacaatgttttatttttctctccatttttctttctttttaatgtttttgtacagatgggatc
ttgctatgttgcctgggctggtctcaaactcctggcctcaatgcatgccccccaccttggcctcccaaagtg
ctgggattgtcaagggctcatttgttacacagtcatagcaacagaaatttaggaactgttacatgtatacc
ttctctttaaagtatttgttacatgtgaaactttacatttgtgtgattactagattcctgtctatcctgag
agactaaaagctcttaacggccaaatttgcgttccttttaccattctattcctgacacttagcaaggtgc
ctggtaaatgatgcactcgatgccaatttactctcaactttcctgcacctcttcacttataaaaagagaaa
gcacttggtcattaattgaacgttcacttatttgatgaatgctgactgagagactgtgtcagggatttgta
ttagagacggacacaaaacaaatatgaaatacaagggactgaggggagacatagaaggggactgggaacac
ttcctgagggaaagggatatttgggctgcgtttagaaggatgtataggtgttttctgaataacaaagaggc
atgacagtacttctggcaaaggaaatggtgagtgccaatgcttgggaccaggaagcagccaggttggtcag
gcaattgtgtgaagttctgcacagaggcaggcagtttgcttggaaaatcctgcaggaaaaggttcctaagg
accaattctgacagcctgcaatgtaggtgcttagatttcatctcattagaaataggaagaccttggaggtt
tggaaggagtggtggaaaaagtggagtttttgtttttctgttttttttttttaagagatgagatgctct
ttaaaaaaaaaaaaaaaaaaaagcactgctatgcttcccaggctggggtgtgcagtggcaagatcatggct
cactgcagccttgaactcttgggcatgagcaatcctgcctcagcctcccgagtagctgggactaaacgtgt
gcaccaccacgcccagctaatttttcattttattttattatttaattaattaattaattaatttttgag
acagagttttgctcttgttgcccaggctggagtgcaatggcgtgatcttggctcactgcaacctccgcct
cccgggttcaagcaattctcttgcctcagcctcccgagtagctgggattacaggcatgcgccacaaagccc
ggctaatttgtatttttagtagagacggggtttctccatgttggtcaggctaatctccaactccagacct
caggtgatctgcccgcctcggcgtcccaaagtgctgggattacaggcgtgagccacggcgcccagccaatt
ttttcatttcttttcttttttcaaggcccggctaatttttgtatttttagtagagacggggttgcac
cgtgttagccaggatggtctctatctcctgacctcgtgatccgtccgcctcggcctcccaaagtgctggga
ttacaggcgtgagccaccatccccagcctagagttcttgatagtatcagggctgagaaagtaccagagtta
gagccaggtggggtggcactaaaagtacaaaaaattagcagggcgcagtggcgggcgcctgtagtcccagc
tactcaggaggctgaggcaggagaatggtgtgaactcgggaggcggagcttgcagtgagcggaaatcgcgc
cactgcactccagcctgggcgacagagcgagactctgtctcaaaaaaaaaaaaaaaaaaaaaaaaaaaaa
agaaagtaccagagttaggaggctcctaaactggtccagatgggaagtgacggggccaaggatagggaagt
gaagatgggaggagtaggactaacttaaaggccatttagcaaaaattcaccacgcaaggccctatattagg
gcgctgggggctcatacctcatcgtcccgtcaactctataaagcaggggccaacaaactttttccataaaa
ggccagatcgtaaatacttcagggtctgcagatctgtaggatctttagcaactacctactctgttgtaagg
cgaaagcaggcagagaagccaggtaaacgcgtgggcgtgtctgtgtgctaataaaactttattcagggacc
ctcaaatgtgaatttcacaaacttttcacaggtcatagactattcttctttggatttttcccccggctatg
taaacatgtaaaaataattctgagcttgcggggccggacaaagtaggcggcgggtcttggctcgcctaccc
ctgctatataaatcattaattatgataccaatcttaaggagaaggtcttcagaagagtccctcaaagagca
ccgcccctcaacgtcatcaaaccccgcctccaaaggcggaggctgcgcatgcgcggcaggacctcgggcgg
gggcggggcggggcgcagCTCCGACTTCGGACATGGCGCACGCTGGCGGTGGCAGCGGTGGCAGCGGTGCC
GGCGGCCCCGCGGGCCGGGGGCTGAGCGGCGCCCGCTGGGGTCGCTCGGGCTCCGCAGGCCACGAGAAGCT
GCCGGTGCAC
